# New approach for accurate discrimination and location of power transformers with different internal winding faults

**DOI:** 10.1371/journal.pone.0309926

**Published:** 2024-10-11

**Authors:** Mohammed Youssef, El-Said Abdelaziz, Hassan Saad, Mohammed Attia

**Affiliations:** 1 Electrical Power and Machines Department, Higher Institute of Engineering, El Shorouk Academy, Cairo, Egypt; 2 Electrical Power and Machines Department, Faculty of Engineering, Al Azhar University, Cairo, Egypt; Ghani Khan Choudhury Institute of Engineering and Technology, INDIA

## Abstract

Power transformers are essential elements in power systems and thus their protection schemes have critical importance. In this paper, a scheme is proposed for accurate discrimination and location of internal faults in power transformers using conventional measuring devices attached to the transformer. Different types of internal winding faults are intensely considered: partial discharge, inter-disk faults, series and shunt short circuit faults and axial displacement. Depending on the transformer measured output voltage, input voltage and the input current, the construction of a locus diagram (ΔV-I_in_) serves as an indicator for any physical modification to the winding. Using five suggested features extracted from the developed locus, an artificial neural network (ANN) technique is applied to accurately distinguish any deviation from the transformer healthy condition. The exact location of each fault inside the windings of power transformer is then determined. The obtained results validate the usefulness of the proposed scheme for different internal faults. The superiority of the proposed scheme is extensively examined by comparing its results with some published schemes.

## I. Introduction

Power transformers consider as a vital component in many zones in the power system networks. They are very expensive and subjected to various types of internal and external faults. According to Ref. [1], 38% of the faults are due to the transformer windings internal faults.

Most internal faults are discovered via differential protection [2, 3]. This classical method depends on measuring and comparing the phase currents in primary and secondary sides. The sensitivity of this technique depends mainly on the precision of current transformer (CT) measurement [4]. Moreover, phase terminals on Δ–connected transformer are not accessible for phase current measurement [4, 5]. The main concern with differential protection is that the exact location within the winding can’t be decided.

One of the earliest tools for internal faults diagnoses in oil transformers is the dissolved gas analysis as the electrical and thermal faults degenerate the liquid insulation and accelerate deterioration of the solid insulation. However, this method is rather expensive and has some ambiguities in its analysis [6]. Meanwhile, it is only valid for oil transformers and also considered as an offline technique for transformer faults diagnoses [2, 7]. On the other hand, frequency response analysis (FRA) is a diagnoses method which can detect the mechanical deformation for all transformer types [8–12]. Accurately interpreting FRA signatures leads to a trustworthy transformer diagnosis [9]. polarization and depolarization current (PDC) technique is present in [13] for oil transformer condition monitoring. FRA is an offline tool [14]. Moreover, it depends on a complicated graphical analysis [2].

On the contrary, to ensure supply continuity by keeping the transformer in service, an adequate online monitoring system should be provided instead of the offline scheduled maintenance [10, 15, 16].

Generally, online monitoring tools are developed using types of controllers which observe the operating conditions of the connected transformer [16, 17]. Recently, online diagnosis techniques were proposed to detect transformer winding deformation. Novel method based on RSLVQ technique for power transformer differential protection was introduced to discriminate between internal fault and inrush currents in the presence of superconductor fault current limiter [18]. AI-based methods for power transformer differential protection were used in [19] in the event of CT saturation state.

An online method has been proposed using synthetic aperture radar imaging and ultra-wideband transceivers [20]. It shows high feasibility to detect the presence of some modeled radial deformations. However, this method has not been simulated on a real transformer winding. Besides, the ability of this method to detect the exact location of the internal faults along the transformer winding has not been introduced.

An online technique was proposed to analyze winding deformations based on the Lissajous graphical analysis with high degree of accuracy. However, the fault discrimination and fault location possibilities were not studied in this research [16].

A new approach to the real-time estimation of GICs in power transformers is presented. An extended Kalman filter (EKF)-based strategy is used [3]. The transformer differential protection’s accessible measurements are utilized by the suggested EKF-based method. The suggested method can effectively handle loading and harmonic excitation. Moreover, quasi-direct currents present in [8] as a disruptive element that mostly arises from interactions between various system components. quasi-direct currents QDCs are present, power transformer inrush currents might significantly rise. QDSs consider as indicator for inrush current.

In this paper, an online monitoring scheme is proposed. The physical change in the winding is detected by tracing the changes in ΔV-I_in_ signature using an accurate ANN algorithm. It is anticipated that each fault type has its electrical response (ΔV- I_in_) locus (signature). The proposed technique is based on examining five-proposed features extracted from the developed locus diagram (ΔV-I_in_). The applied method can handle massive amounts of data, inconsistent data, overlapping data, or missing data.

The following factors should be considered while determining the fault’s location in addition to winding fault detection:

- In a power transformer, some types of internal faults, like a turn’s short circuit, are crucial. It must be identified early to avoid total failure [14].
- Maintenance become simpler and quicker when the type and location of failures are recognized, which eliminates the overall cost of repairs.

In this work, several mechanical and electrical winding faults in different locations applied to a 3-phase transformer winding to find which disk is faulty.

The paper is structured as follows: the transformer modeling and the parameters of the case study are discussed in Section II. The description of all examined faults and their effects are fully introduced in Section III. Section IV presents the common extracted features for identification of internal defects within the transformer, in addition to the proposed features which can be calculated from general dimensions of the ellipse. Implementing the proposed technique for faults identification and location is fully illustrated in Section V. The obtained study results for extensive simulations of tested fault conditions are shown in Section VI, while Section VII introduces a comparison of the proposed methodology with some other published research works, and Section VIII draws the conclusion.

## II. Transformer modeling

**Fig 1:**
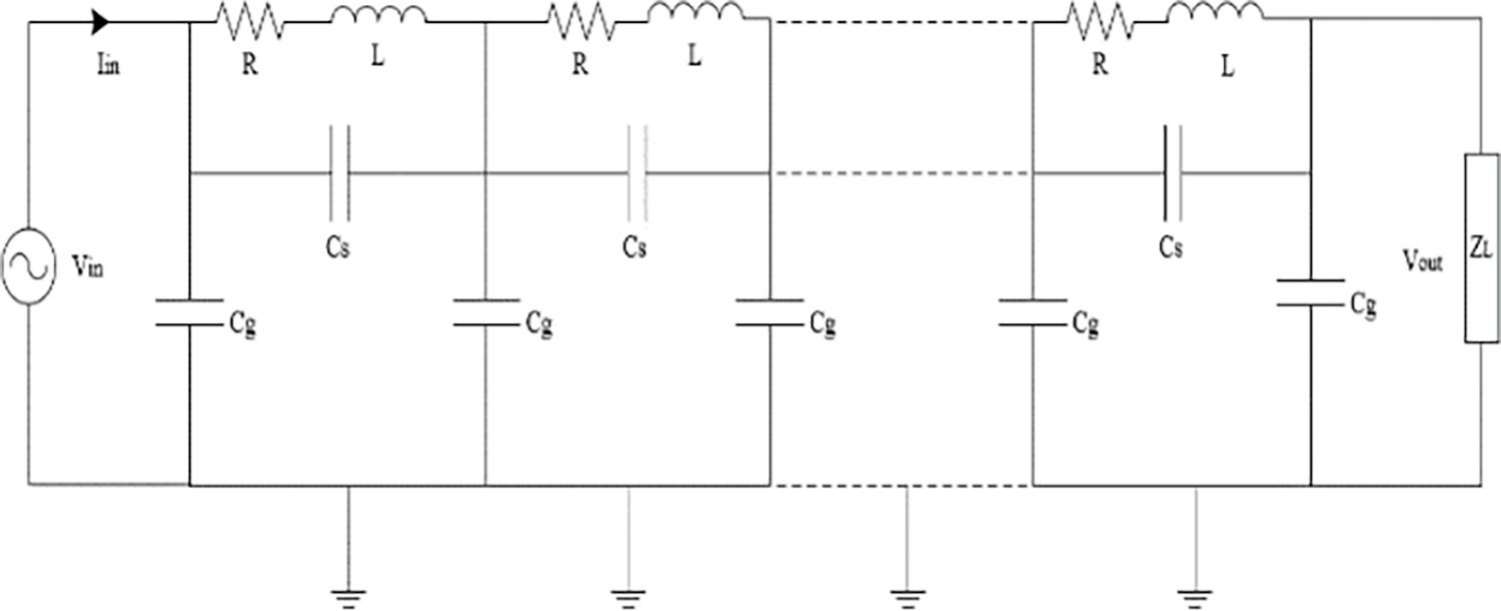
Modelling of internal winding of power transformer.

In this paper, the 33/11 kV HV side delta-connected disc-type interleaved winding, 3 MVA, 50 Hz, ON, DY11; three-phase power transformer is considered. The transformer distributed model shown in Fig 1 [21] has been applied in this paper.

**Fig 2:**
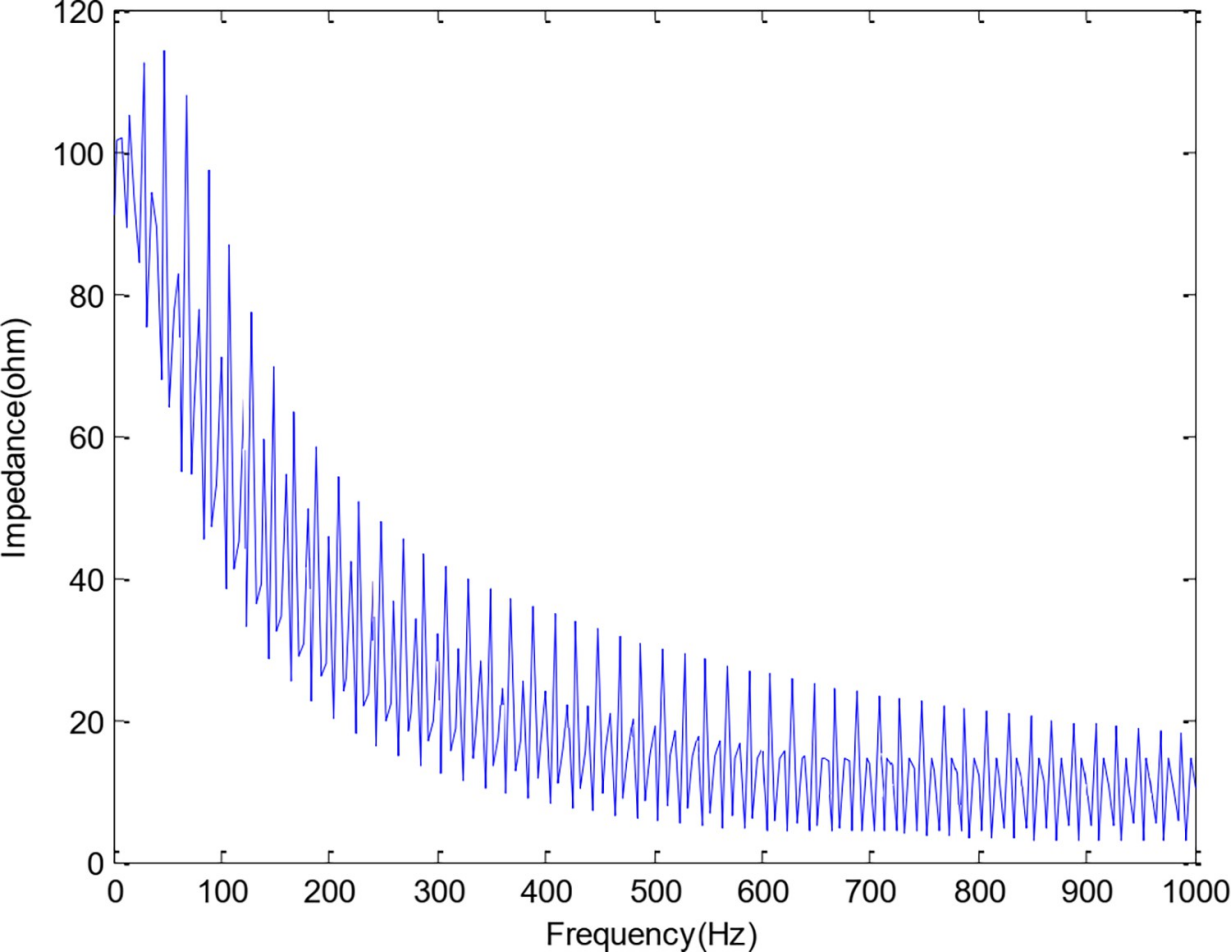
One sided amplitude spectrum for transformer terminal impedance.

The simulated model consists of 88 disks connected in series. The detailed transformer model parameters are given in Table 1 [22]. To calculate the one-sided amplitude spectrum of the impedance for transformer model shown in Fig 1, frequency response analysis is presented in Fig 2 for terminal impedance.

**Table 1:** Detailed parameters for transformer model [22].

Parameter	Value
Number of discs	88
Winding inductance (L)	0.324 mH
Winding losses (R)	0.151 Ω
Series capacitance (C_s_)	1.04 nF
Ground capacitance (C_g_)	22.13 pF

## III. Simulation studies

As mentioned earlier, power transformers have a lot of faults types, while the internal faults are considered the substantially important one. The internal faults can be divided into five main types as described in Table 2 [23].

**Table 2:** Common internal faults in power transformers.

Fault type	Description
**PDF**	Partial Discharge Fault
**IDF**	Inter Disk Fault
**SEF**	Series short circuit Fault
**SHF**	Shunt short circuit Fault
**ADF**	Axial Displacement Fault

**Fig 3:**
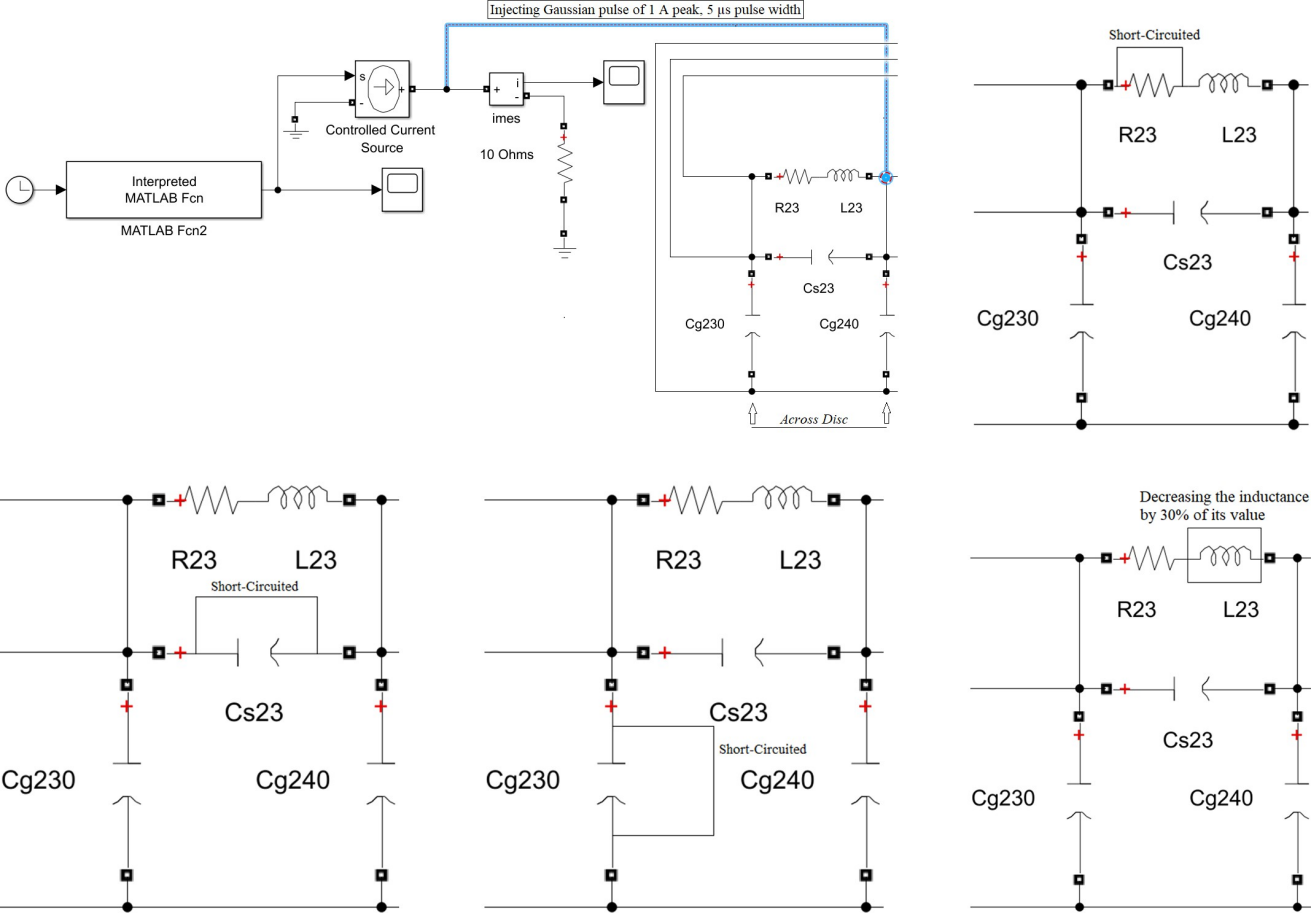
Simulation model for five internal faults in power transformers. (A) Partial Discharge Fault. (B) Inter Disk Fault. (C) Series short circuit Fault. (D) Shunt short circuit Fault. (E)Axial Displacement Fault.

**Fig 4:**
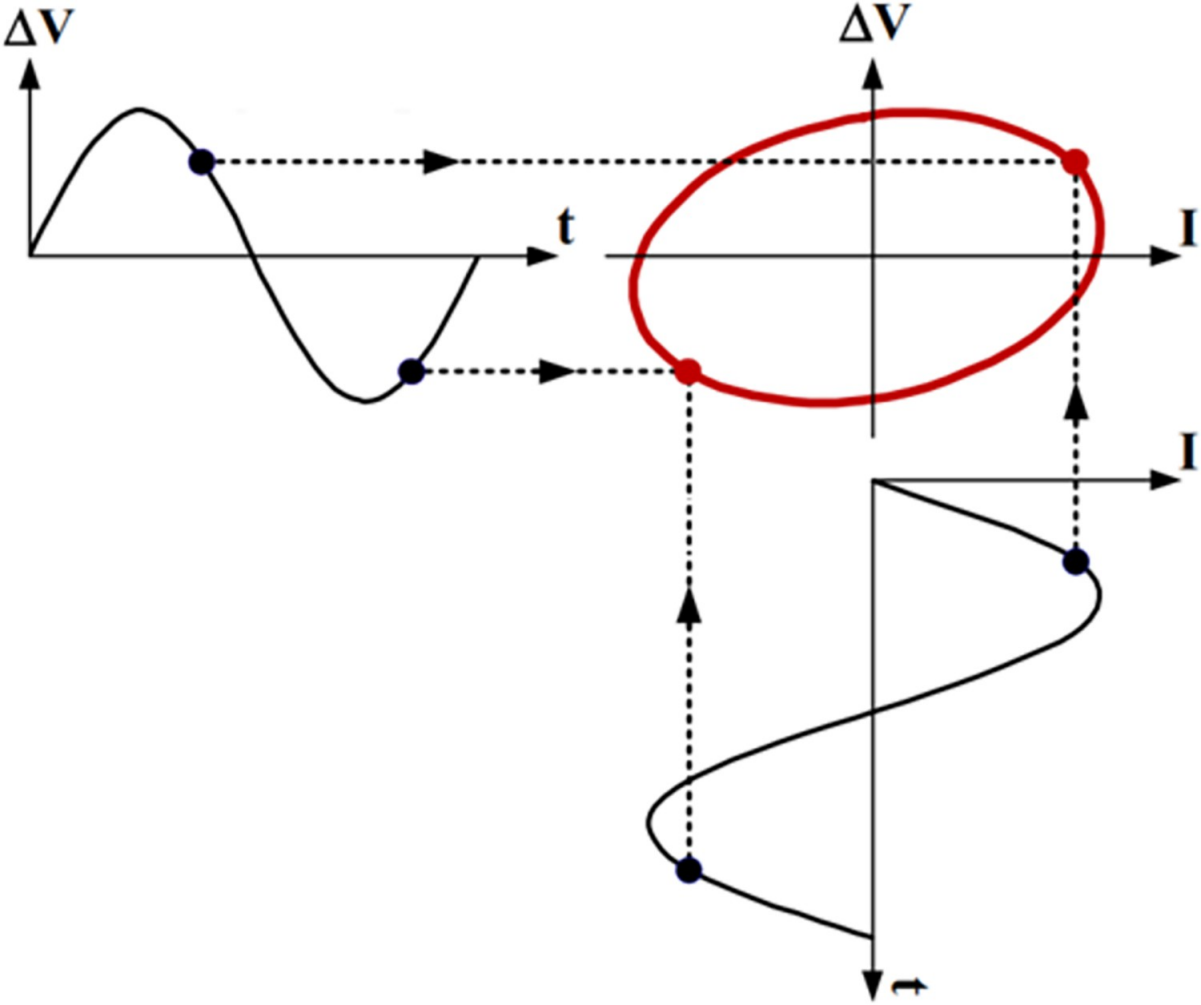
Constructed (ΔV-Iin) locus as a mark of the physical variation in transformer winding.

A MATLAB software used to simulate five internal faults along the power transformer winding. The simulation technique for these internal faults is clarified in Fig 3. The transformer model in Fig 1 is used to illustrate the various internal fault types. For each full power cycle, the input voltage (*V*_*in*_), input current (*I*_*in*_), and output voltage (*V*_*out*_) at the power frequency (50 Hz) are recorded. Constructing a locus diagram as a signature to physical changes in the transformer winding is the main target of the proposed online monitoring technique. As shown in Fig 4, this locus displays the transformer input current on the *x* axis and the difference between a phase’s input and output voltages (*V*_*in*_-*V*_*out*_) on the *y* axis.

In the following subsections, the techniques used to simulate different types of internal faults are reviewed.

### a. Partial discharge (PD)

**Fig 5:**
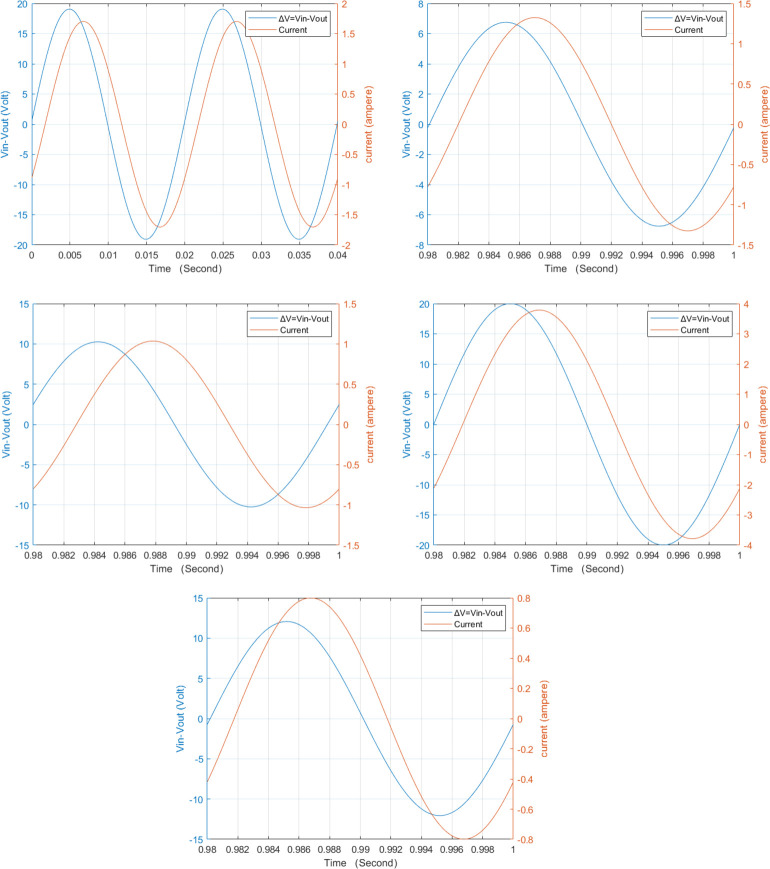
Voltage and current time domain plotting at 60 faulty disk location for five internal fault cases. (A) ΔV and Iin waveforms in case of PDF. (B) ΔV and Iin waveforms in case of IDF. (C) ΔV and Iin waveforms in case of SEF. (D) ΔV and Iin waveforms in case of SHF. (E) ΔV and Iin waveforms in case of ADF.

**Fig 6:**
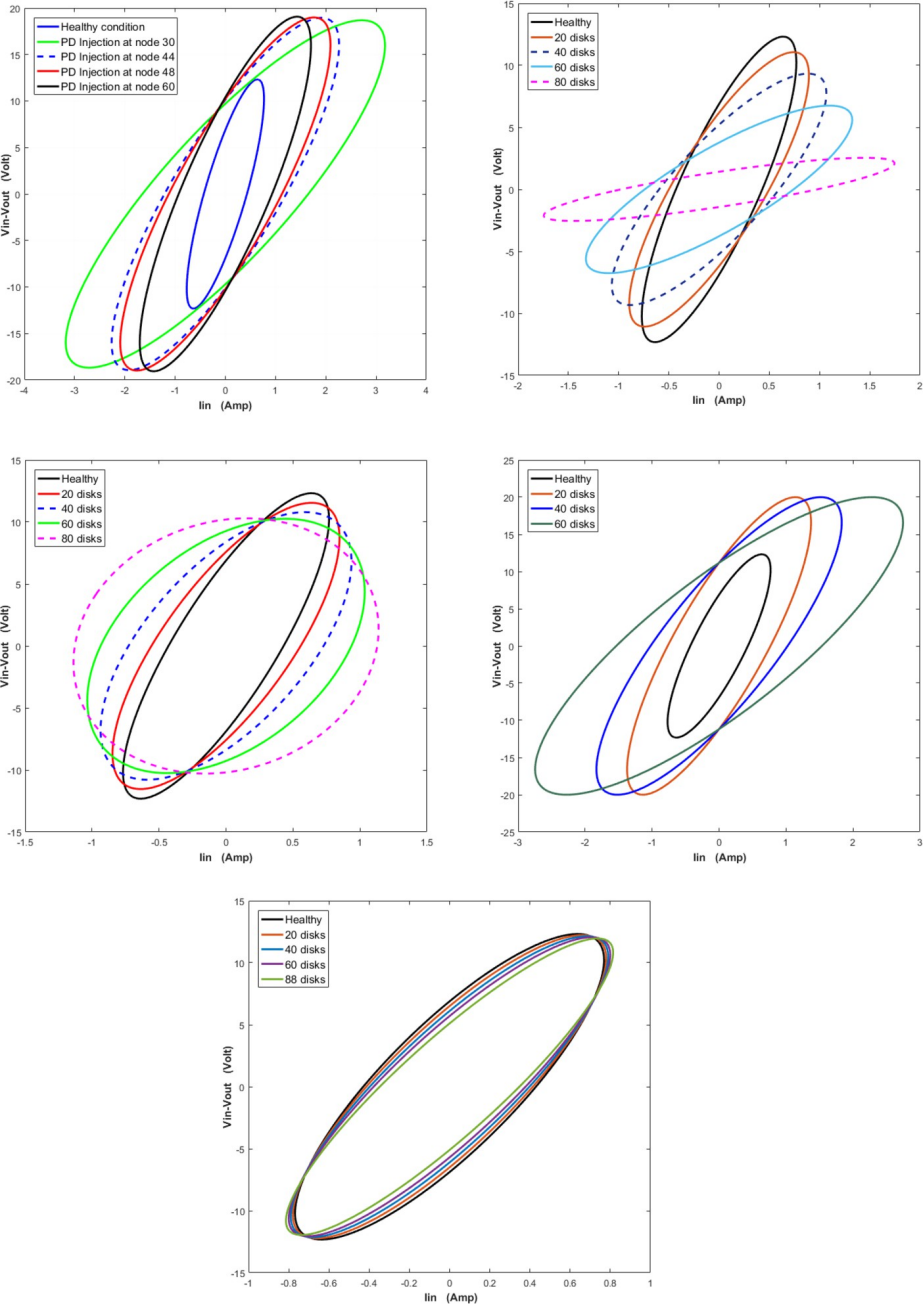
The locus (ΔV-Iin) for different fault types. (A) Effect of partial discharge fault (PDF). (B) Effect of inter-disk faults (IDF). (C) Effect of series short circuit faults (SEF). (D) Effect of shunt short circuit faults (SHF). (E) Effect of axial displacement fault (ADF).

Partial discharge (PD) is considered as a chance occurrence that is influenced by the electric field [16]. Power transformers electric insulation typically degrades when electrical charges are present [5, 21]. The PD can be simulated by injecting a current pulse which has a shape equivalent to the practical PD pulses into probable positions of the windings [6]. The PD current pulse is simulated in this paper by a Gaussian pulse of 1 A peak, 5 μs pulse width as in [24]. Current and voltage waveform is shown in Fig 5A For 60 faulty disks. Fig 6A illustrates the produced locus for 30, 44, 48 and 60 defective discs relative to the loci of the healthy condition. It can be deduced that the locus rotates clockwise and expands in size as the number of defective discs’ increases.

### b. Inter-disk faults (IDF)

Inter-disc fault or (turn to turn short circuit) consider the most widespread types of internal faults. IDF are responsible for about 34% of transformer failure [21, 24]. In this paper, IDF simulation achieved by short circuiting the series resistors of various numbers of discs [16]. Fig 5B illustrate the effect on ΔV and Iin in time domain at 60 faulty disks. To investigate the effect of IDF on the developed (ΔV- Iin) locus, different cases are simulated as shown in Fig 6B. It displays the loci for inter-disc faults at 20, 40, 60 and 80 disks compared to the locus of the healthy condition. As shown, the locus rotates clockwise and expands in area as the number of faulty discs’ increases.

### c. Series short-circuit faults (SEF)

The insulating breakdown between the discs is implied by the series fault [16]. To model a SEF, the defective disc has been short-circuited [21]. To investigate the effect of SEF on (ΔV- Iin) signals, Voltage and current signal are plotted as Fig 5C at the same 60 defective disk. To realize the effect of SEF occurrence on the (ΔV- I_in_) relation, the locus is developed when series short-circuit faults occur at 20, 40, 60 and 80 disks against the healthy locus as illustrated in Fig 6C. It is visible that the locus rotates in a clockwise manner and that its area decreases as the number of defective discs rises.

### d. Shunt short-circuit faults (SHF)

The primary causes of leakage faults or disc to ground fault inside a transformer include insulation failure, ground shielding injury, abrasion, high levels of moisture in the coils, hotspot, and aged insulation [1, 2]. Shunt faults signify a breakdown in the insulation between the earthed items (tank, core, etc.) and the winding [16, 21]. In the simulated transformer model, this type of fault is modeled by connecting the faulty disc to ground. Fig 5D show the effect of SHF on voltage/current signals waveform. Fig 6D shows the constructed loci for 20, 40, 60 and 80 faulty disks against the healthy condition one. It can be detected that the formed locus rotates in a clockwise orientation and expands in total area as the number of defective discs rises.

### e. Axial displacement (AD)

In the case of short circuit currents, both low voltage and high voltage windings are magnetically unbalanced, AD results. Generally, by modifying the mutual and self-inductances of the discs, while ignoring the change in capacitance, this type of failure can be modeled. In the simulated model, the AD is achieved by decreasing the inductance by 30% of its value as in [2, 16, 21]. To highlight the effect of axial displacement, the (ΔV- I_in_) loci that represent axial displacement at 20, 40, 60 and 88 disks are compared to the locus of the healthy condition as illustrated in Fig 6E. for 60 defective disks, ΔV and I_in_ signal are shown in Fig 5E in time domain.

## IV. Features extracted for internal faults identification

**Fig 7:**
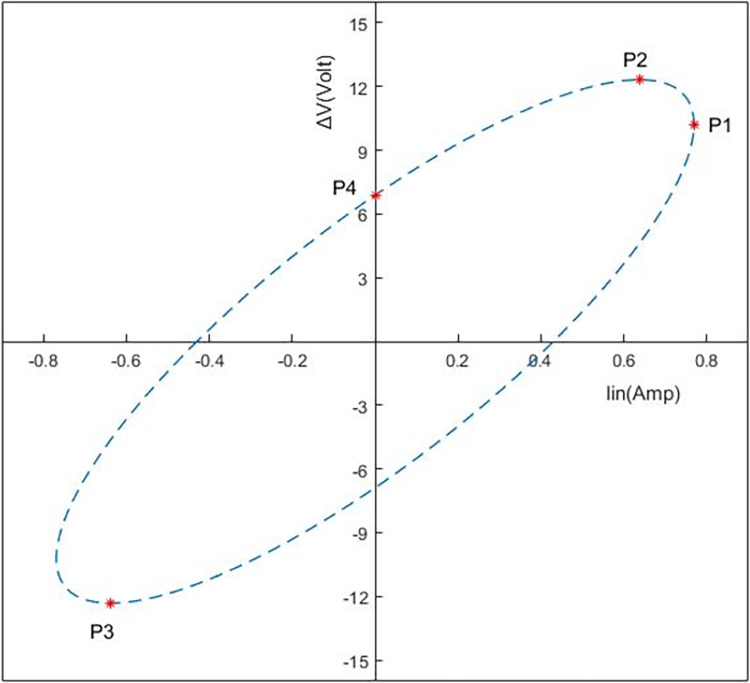
Four required points on (ΔV-Iin) locus to get the five proposed extracted features.


Identifying critical features for internal fault detection within power transformers remains an ongoing area of research. Some commonly extracted features from the developed ellipse general dimensions have been addressed in the literature [16, 25]. The semi-major A and minor axis length B (see Fig 7) are among these features, and they are derived using Eqs 1 and 2 respectively utilizing three parameters: the maximum value of x-axis (a), the maximum value of y-axis (b), and power factor (cos φ). In addition, Eq 3 provides the angle between the horizontal axis and the semi-major axis (θ). Eqs 4, 5, and 6 are used, respectively, to compute the first and second ellipse eccentricities (e), (e’), as well as the relationship between them. The ellipse focus (f) and flattering (g) can be determined using Eqs 7, and 8, whereas Eqs 9 and 10 provide the formulas for calculating the area and circumference of the ellipse respectively.

$$A=\sqrt{\frac{1}{2}(a^{2}+b^{2})+\frac{s}{2}\sqrt{(a^{2}-b^{2})+4a^{2}b^{2}{cos}^{2}\varphi }}$$ (1)

$$B=\sqrt{\frac{1}{2}(a^{2}+b^{2})-\frac{s}{2}\sqrt{(a^{2}-b^{2})+4a^{2}b^{2}{cos}^{2}\varphi }}$$ (2)

$$\theta =\frac{1}{2}\tan^{-1}\left(\frac{2AB}{A^{2}-B^{2}}cos\varphi \right)$$ (3)

$$e=\frac{\sqrt{A^{2}-B^{2}}}{A}$$ (4)

$$e`=\frac{\sqrt{A^{2}-B^{2}}}{B}$$ (5)

$$\frac{e}{e`}=\frac{B}{A}$$ (6)

$$f=\sqrt{A^{2}-B^{2}}$$ (7)

$$g=1-\frac{B}{A}$$ (8)

Aellipse=πAB (9)

$$c=\pi [3(A+B)-\sqrt{10AB+3(A^{2}{+B}^{2})}]$$ (10)

The aforementioned ten-features represent the most significant features used in the literature for identifying insulation break down [16, 26–29]. These calculated features depend on the ellipse dimensions under faulty condition as shown in S1 and S2 Tables. The dimension of ellipse may vary with very minor change which may lead to low identification accuracy.

In [30], Multi-dimensional wavelet network and artificial neural network have been used for insulation failure identification. The overall classification accuracy of characteristics identification is approximately 91% and 76.16% respectively. In [31], cross correlation and other mathematical patterns-based features are developed to train ANN classifier technique. The detection’s overall accuracy can approach 98.8% for identification for only three types of internal faults.

## V. Proposed technique for internal faults identification & location

For high level and accurate insulation failure identification, a MATLAB algorithm is developed in this paper for extracting new features (F1, F2, F3, F4 and F5) from (ΔV- I_in_) locus as shown in S2 Table. The five proposed features are extracted using the coordinates of the four points P1, P2, P3 and P4 shown in Fig 7.

The first proposed feature F1 represents the maximum value of I_in_. and is determined by the x-coordinate of point P1.

The second and third feature (F2 and F3) represent the minimum and the maximum value of ΔV. They are calculated using the y-coordinate of points P2 and P3 respectively.

The fourth feature F4 represents the maximum value at the intersection point of the ellipse with y-axis (P4). Finally, the fifth proposed feature F5 is defined as the absolute value of point P3 which is given by Eq 11, where (*x*)P3, (*y*)P3 are the x, y coordinate values of P3 respectively.


$$F5=\sqrt[{2}]{{\left[(x)P3\right]}^{2}+{\left[(y)P3\right]}^{2}}$$ (11)

As previously stated, the proposed scheme based on recording the values of (ΔV- I_in_) relation. These measurements are achieved with the use of measurement tools which already installed to the power transformer. The locus diagram is restored every one cycle (20 ms). Such locus diagram will be affected by any type of defect inside the transformer winding; and hence, to determine the fault type, the extracted Five-proposed features are used.

Recently, several power system issues have been identified and mitigated using artificial intelligence techniques. Learn Vector Quantization (LVQ) networks have found to be superior to different algorithms of neural networks for faults identification and location in power networks [32, 33].

**Fig 8:**
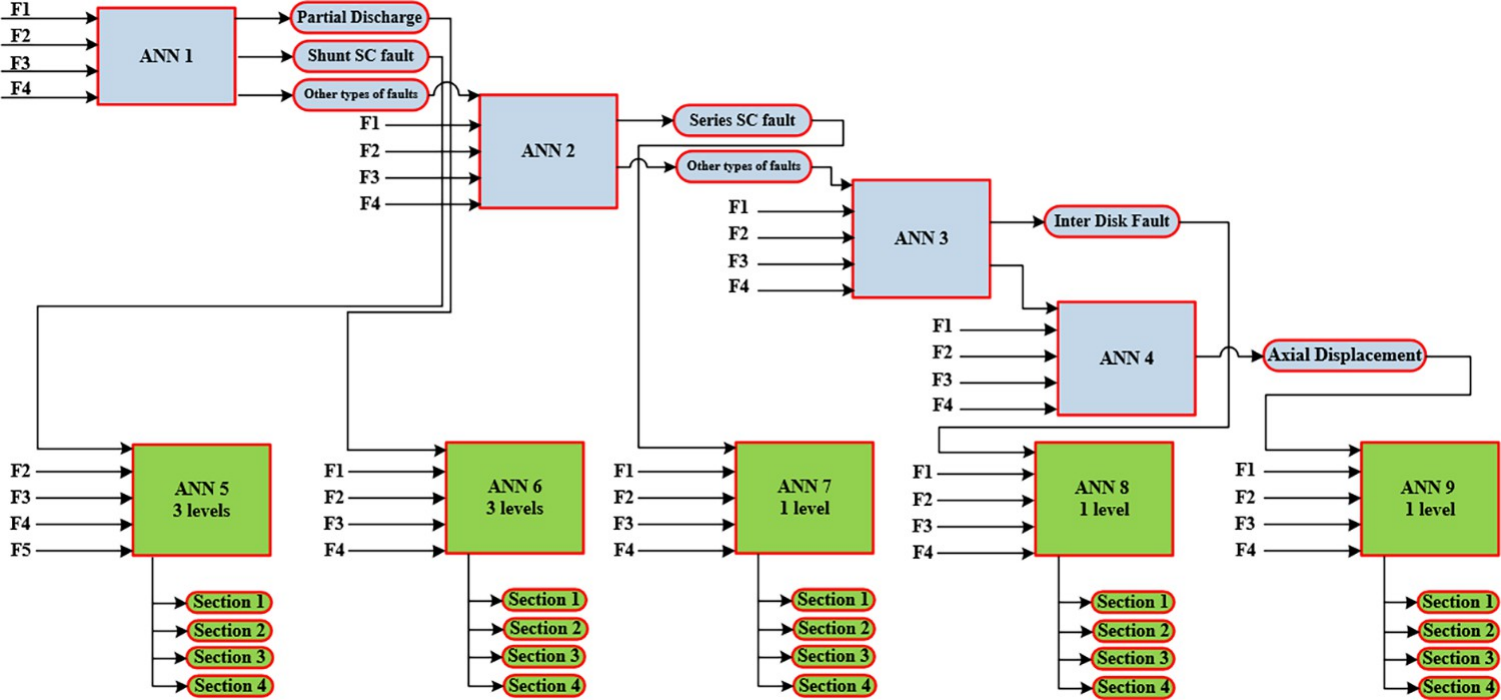
Flowchart of faults identification and location algorithms.

In this paper, a multi-level LVQ network for fault diagnosis is applied to classify the transformer internal fault types into five states. The five states of LVQ fault identification model include partial discharge fault, inter-disk fault, series short fault, shunt short fault and axial displacement fault. Fig 8 present the flowchart for complete fault identification and location algorithms showing the order number and function of LVQs networks.

There are 88 various locations along the transformer winding that the transformer understudy is exposed to five different fault types. So, theoretically the entire matrix of data that can be used for fault identification has 440 rows (5 fault types × 88 different locations) and 5 columns (five proposed features). Preconditioning the input data for classification-based neural networks is a significant issue, it is noteworthy that the matrix of data that is used to identify faults is reduced to only 440 rows (5 fault types × 88 different locations) and 4 columns (four out of five proposed features). The reduction is applied to reduce the ANN training process while guaranteeing acceptable performance.

As shown in Fig 8, the fault identification process is described using 4-levels LVQ network (ANN 1 to ANN 4) as follows:

- **Level 1 (ANN 1):** The first LVQ is designed to classify the partial discharge and shunt short circuit faults while the other three fault types are distinguished in next ANN levels.
- **Level 2 (ANN 2)**: This level of LVQ model is applied to classify series short circuit faults from the data set for the three types remaining. For discrimination the inter-disk fault and the axial displacement, levels 3 and 4 are applied.
- **Level 3 (ANN 3)**: This level classifies inter-disk fault from the remaining data set.
- **Level 4 (ANN 4)**: classifies axial displacement faults.

After the fault identification process is completed, another sub-classification multi-level LVQ algorithm is used to decide exactly the location of the classified fault. The total HV winding of the transformer is divided into four sections as illustrated in Table 3. The fault is then localized within one of these four sections.

**Table 3:** Sections describe transformer disks distribution.

Sections No.	From disk no.	To disk no.
**Section 1** (Sec. 1)	1	22
**Section 2** (Sec. 2)	23	44
**Section 3** (Sec. 3)	45	66
**Section 4** (Sec. 4)	67	88

A dimensional data matrix, m×n, is formed by the features obtained from the produced ellipse of each fault type. where m is the locations for any fault case and n is location features extracted from various faults. In the transformer winding, m is 440 samples representing five different aberrant conditions at 88 different positions. So, the multi-level LVQ localization algorithm uses a data matrix contains 440 locations cases (rows) and 5 features (columns).

**Fig 9:**
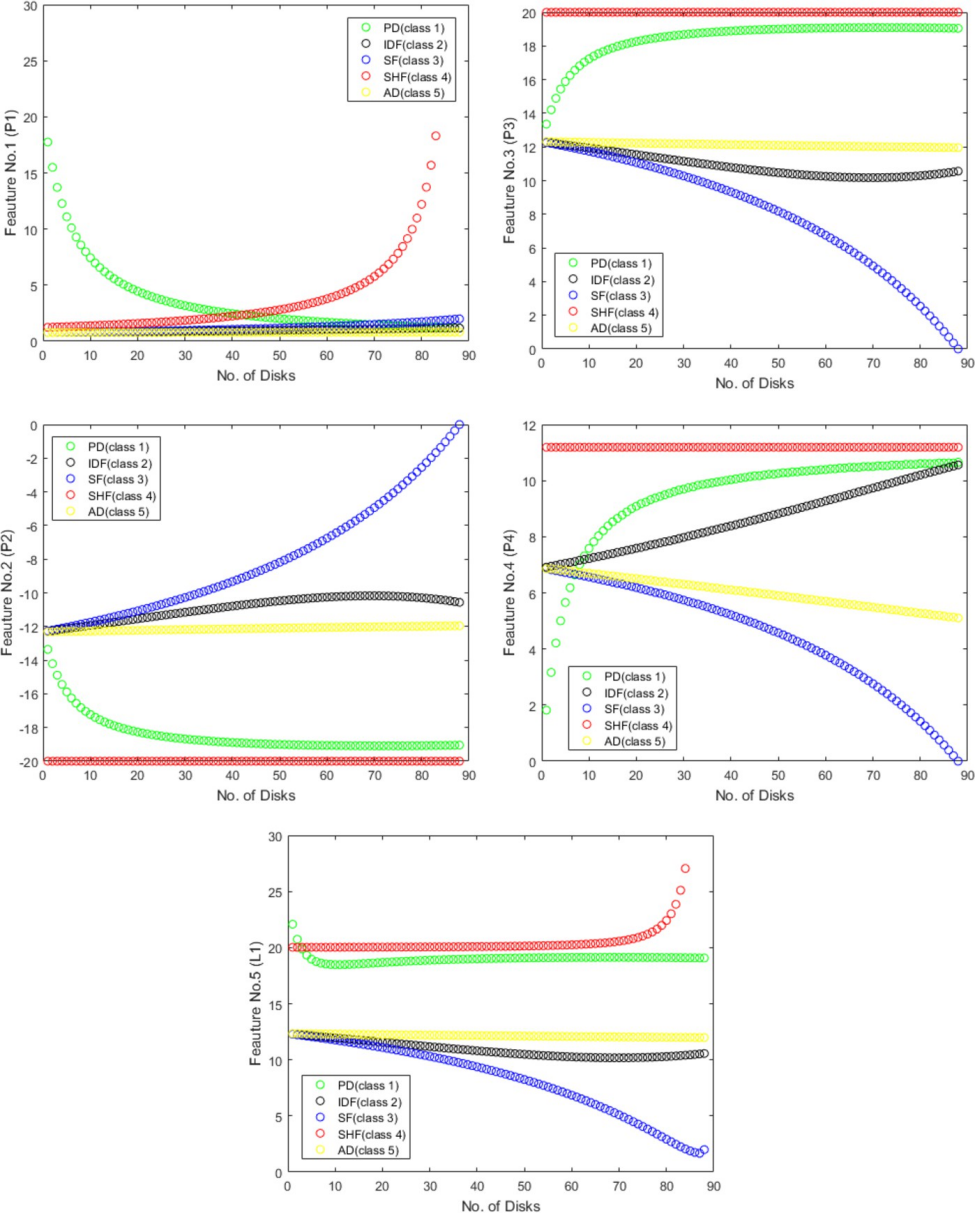
Proposed features variation versus no. of faulty disks for all fault types. (A) First feature variation (F1). (B) Second feature variation (F2). (C) Third feature variation (F3). (D) Fourth feature variation (F4). (E) Fifth feature variation (F5).

As shown in Fig 8, the fault location process is achieved using five ANNs (ANN 5 to ANN 9). Each ANN belongs to specific fault type. The five applied features for each ANN are also illustrated in Fig 9.

In the following, description of such five ANNs is introduced:

- **ANN 5:** Using ANN 5 (consists of 3-levels LVQ network), the faulty section in case of shunt short circuit faults is localized.
- **ANN 6:** Using ANN 6 (3-levels LVQ network), the faulty section in case of partial discharge faults is determined.
- **ANN 7:** Using ANN 7 (1-levels LVQ network), the faulty section in case of series short circuit faults is determined.
- **ANN 8:** Using ANN 8 (1-levels LVQ network), the faulty section in case of Inter-disk faults is determined.
- **ANN 9:** Using ANN 9 (1-levels LVQ network), the faulty section in case of axial displacement faults is determined.

## VI. Validation results and discussion

As discussed before, this research work investigates utilizing five proposed features to accurately classify and locate five different fault types. The proposed features are comprehensively investigated for all fault types along the transformer. Each part in Fig 9 represents (in the y-axis) the distinguished variation in the values of one of the proposed five-features (F1, F2, F3, F4 or F5) with the different five fault types under study (different colors) and at different disk location (x-axis). This highlight the suitability of the proposed features to be used in the classification and localization process. Based on the achieved results, it is concluded that four out of the proposed five features can be applied for each ANN. The corresponding features to each ANN are shown in the flowchart of Fig 8.

As previously mentioned, the entire data matrix required for the fault identification process is 440 rows by 4 columns, and for the fault location process, it is 440 rows by 5 columns. In the presented work, half of the total data is applied to train the LVQs for the identification strategy and location strategy, and the other half is used to test the validity and accuracy of the scheme.

Using four features (F1 to F4), the identification algorithm effectively identified all of the defective loci in one minute and 20 seconds of training time, as shown in Table 4.

**Table 4:** Overall fault identification results.

ANN description and training features	Four levels ANN, 220 fault cases for training procedure, zero training error				
**No. of tested cases**	**220 fault cases**				
	**44**	**44**	**44**	**44**	**44**
	**partial Dis.**	**Shunt Fault**	**Series Fault**	**Inter Disk**	**Axial Disp.**
**No. of false identification cases**	**0**	**0**	**0**	**0**	**0**
**Fault identification accuracy**	**100%**	**100%**	**100%**	**100%**	**100%**
**Overall accuracy**	**100%**				

For PDF location process (as an example), the algorithm has successfully located 434 faulty disks from 440 total faulty disks in 3 min and 19 second training time The fault location accuracy of partial discharge faults is about 95.45% as illustrated in Table 5.

**Table 5:** Partial discharge fault location results.

Fault Type	Partial Discharge			
**ANN description and training features**	**Three levels ANN, 44 fault cases for training procedure, zero training error**			
**No. of tested cases**	**44 fault cases**			
	**11**	**11**	**11**	**11**
	**Sec. 1**	**Sec. 2**	**Sec. 3**	**Sec. 4**
**No. of false location cases**	**0**	**1**	**1**	**0**
**Predicted location section for false cases**	**-**	**Sec 3**	**Sec 4**	**-**
**Fault localization accuracy**	**100%**	**90.9%**	**90.9%**	**100%**
**Overall accuracy**	**95.45%**			

The overall fault location accuracy of the proposed scheme for each type of faults within power transformer is summarized in Table 6.

**Table 6:** Overall fault location results.

Fault type	Fault Location Accuracy				
	Sec. 1	Sec. 2	Sec. 3	Sec. 4	Overall accuracy
**Partial Discharge (PD)**	**100%**	**90.91%**	**90.91%**	**100%**	**95.45%**
**Inter-disk fault (IDF)**	**100%**	**90.91%**	**90.91%**	**100%**	**95.45%**
**Series short-circuit fault (SEF)**	**100%**	**90.91%**	**100%**	**100%**	**97.72%**
**Shunt short-circuit fault (SHF)**	**100%**	**100%**	**100%**	**100%**	**100%**
**Axial displacement (ADF)**	**100%**	**100%**	**90.91%**	**100%**	**97.72%**

## VII. Comparing proposed scheme with other methods

Table 7 presents a brief comparison of the proposed method with some published methods for fault identification and location [31, 34–40]. The proposed scheme has better capability to distinguish internal faults within power transformer. It has an overall accuracy of 100% compared to 99.95% [40], 99.07% [36], 94.7% [38] 87.18% [35] and 85.71% [39], overall accuracy using support vector machine (SVM) in applying various types of fault diagnosis techniques: differential protection, transfer function measurement, and dissolved gas analysis respectively.

**Table 7:** Comparison of the proposed method with several alternative methods for internal fault identification and location.

Item		Proposed Method	Shah et al. [34] 2016	Patel et al. [40] 2021	Zhang et al. [35] 2016	A. J. et al. [31] 2014	Bhalja et al. [36] 2013	Bigdeli et al. [37] 2013	YIN et al. [39] 2011	Vakilian et al. [38] 2012
**fault identification**	**Used method**	(ΔV-I_in_) locus	Differential protection	XGBoost	Dissolved gas analysis (DGA)	Frequency response analysis (FRA)	Differential protection	Transfer function measurement	Cross correlation	Transfer function measurement
	**Applied technique**	Learning vector quantization (LVQ)	Random Forest technique (RF)	convolution neural network (CNN)	Support vector machine (SVM)	ANN-feed forward back propagation	Support vector machine (SVM)	probabilistic neural network (PNN)	SVM-Genetic Algorithm	Support vector machine (SVM)
	**Fault types list**	**1.** PDF **2.** IDF **3.** SHF **4.** SEF **5.** ADF	**1.** L–G **2.** L–L **3.** L–L–G **4.** Inter-winding **5.** Turn-to-turn	**1.** Turn-turn **2.** Prim.-Sec. winding fault **3.** Internal winding fault	**1.** LE-D * 2. HE-D ** 3. LM-T *** 4. H-T **** 5. N-C ****	**1.** disc-to-disc SC **2.** radial deformation **3.** axial displacement	**1.** L–G **2.** L–L **3.** L–L–G **4.** Prim.-Sec. winding **5.** Turn-to-turn	**1.** Axial displacement **2.** Radial deformation **3.** Disc space variation **4.** Short circuit	**1.** Single fault **2.** Discharge **3.** Overheating **4.** Devided as **5.** High, middle low temp.	**1.** Axial displacement **2.** Radial deformation **3.** Disc space variation **4.** Short circuit
	**No. of tested cases**	220	3240	36180	117	N/S	6420	19	N/S	19
	**No. of false cases**	0	54	18	15	N/S	60	1	N/S	1
	**Fault identification accuracy**	100%	98.33%	99.95%	87.18%	98.8%	99.07%	94.7%	85.71%	94.7%
**Fault Location**	**Technique**	A	N/A	N/A	N/A	A	N/A	N/A	N/A	N/A
	**Accuracy**	97.27%	N/A	N/A	N/A	95.4%.	N/A	N/A	N/A	N/A

Where A = Applied, N/A = Not Applied, N/S = Not Specified.

*LE-D: Low-energy discharge.

**HE-D: High-energy discharge.

***LM-T: Low and medium temperature.

****H-T: high temperature.

****N-C: normal condition.

Moreover, the artificial neural network based on frequency response analysis and transfer function measurement gives an overall accuracy of 98.8% [31] and 94.7% [37], respectively. Besides, random forest technique has been introduced with accuracy 98.33% in [34].

Moreover, the proposed scheme has approved superior competency to locate five types of internal faults within power transformer with a reasonable accuracy of 97.27% compared to only three internal faults types and 95.4% accuracy as introduced in [31].

## VIII. Conclusion

Five frequent internal transformer failures are discussed in this work, namely, partial discharge, inter-disk, series short -circuit, shunt short-circuit and axial displacement at different locations were simulated to 33 kV winding of a 3 MVA power transformer. Successful discrimination and location of these insulation failures is achieved using a proposed scheme. The (ΔV- I_in_) locus is constructed using the signal of input voltage, current and output voltage. Five proposed features are sufficient for detecting the variation in the (ΔV- I_in_) locus for various forms of insulation failure. The identification and localization of any deformation in power transformers winding is proposed using a multi-level neural network approach based on analytical feature training sets. The fault identification algorithm has comprised four learn vector quantization levels to identify all internal fault types in effective manner. Moreover, multi-level neural networks are implemented based on the extracted features and (ΔV- I_in_) locus to locate which section has the faulty disk in the transformer.

The achieved fault identification results showed that four features are sufficient to get a reasonable accuracy of the identification process. From a total of 440 defective loci, the proposed approach successfully recognized 440 of them, with a 100% overall identification accuracy. Besides, the overall location accuracy of about 97.27% has been achieved from the proposed location algorithm.

Finally, the proposed scheme has proved its effectiveness to accurately discriminate and locate internal transformer winding faults. Thus, it can be applied as a significant tool for evaluating the status of transformers that enables power management systems to identify those that need prompt periodic repair or replacement without causing a supply interruption.

## Supporting information

S1 Table General ellipse parameter features and extracted features from locus for transformer healthy condition. (PDF)

S2 Table General ellipse parameter features and extracted features from locus for five internal faults: -Partial discharge fault, inter-disk fault, series short circuit, shunt short circuit and axial displacement respectively. (PDF)
