# Supplementary material for: New approach for accurate discrimination and location of power transformers with different internal winding faults
Source: PLoS One. 2024 Oct 11;19(10):e0309926. doi: 10.1371/journal.pone.0309926 (PMC11469500; doi:10.1371/journal.pone.0309926)
Supplement: S1 Table — (PDF) [file pone.0309926.s001.pdf]

	1. General ellipse parameter features									
	A\`	B\`	θ	f	e	e'	$\frac{e}{e'}$	g	$A_{ellipse}$	$C_{ellipse}$
Healthy condition	11.8046275	3.60479261	86.42967621	11.24076068	0.952233408	3.118282215	0.305371144	0.694628856	133.6849273	51.90001542

	2. features extracted from locus																					
	I_{min}	ΔV_{Imin}	θ_{Imin}	Abs_{Imin}	I_{max}	ΔV_{Imax}	θ_{Imax}	Abs_{Imax}	ΔV_{min}	$I_{\Delta Vmin}$	$\theta_{\Delta Vmin}$	$Abs_{\Delta Vmin}$	Δv_{max}	$I_{\Delta Vmax}$	$\theta_{\Delta Vmax}$	$Abs_{\Delta Vmax}$	$\Delta V_{H(0)}$	$\Delta V_{L(0)}$	$I_{H(0)}$	$I_{L(0)}$
Healthy condition	-0.768714059	-10.21382149	94.30408852	10.242709	0.768714027	10.21382232	85.69591201	10.242709	-12.31879844	-0.637201668	92.96104022	12.33526736	12.31879808	0.637201593	87.03896004	12.335267	6.886995039	-6.886995039	-0.429994586	0.429994586
