# Supplementary material for: New approach for accurate discrimination and location of power transformers with different internal winding faults
Source: PLoS One. 2024 Oct 11;19(10):e0309926. doi: 10.1371/journal.pone.0309926 (PMC11469500; doi:10.1371/journal.pone.0309926)
Supplement: S2 Table — (PDF) [file pone.0309926.s002.pdf]

partial discharge	***1. General ellipse parameter features***										***2. features extracted from locus***																			
faulty disk	A`	B`	θ	f	e	e'	$\frac{e}{e'}$	g	$A_{ellipse}$	$C_{ellipse}$	I_{min}	ΔV_{Imin}	θ_{Imin}	Abs_{Imin}	I_{max}	ΔV_{Imax}	θ_{Imax}	Abs_{Imax}	ΔV_{min}	$I_{\Delta Vmin}$	$\theta_{\Delta Vmin}$	$Abs_{\Delta Vmin}$	Δv_{max}	$I_{\Delta Vmax}$	$\theta_{\Delta Vmax}$	$Abs_{\Delta Vmax}$	$\Delta V_{H(0)}$	$\Delta V_{L(0)}$	$I_{H(0)}$	$I_{L(0)}$
1	22.1643	1.52227	36.9541	22.112	0.99764	14.5257	0.06868	0.93132	105.997	89.285	-17.7533	-13.232	143.302	22.1419	17.7533	13.232	36.6982	22.1419	-13.3565	-17.586	142.783	22.0831	13.3565	17.586	37.2166	22.0831	1.8192	-1.8192	-2.4316	2.4316
2	20.8931	2.36307	42.5067	20.759	0.99358	8.78478	0.1131	0.8869	155.106	85.1579	-15.4997	-13.8506	138.216	20.7865	15.4997	13.8506	41.7842	20.7865	-14.2079	-15.1082	136.759	20.7394	14.2079	15.1082	43.241	20.7394	3.16609	-3.16609	-3.46163	3.46163
3	20.0357	2.88841	47.3522	19.8264	0.98955	6.86411	0.14416	0.85584	181.808	82.4712	-13.7131	-14.2828	133.834	19.8002	13.7131	14.2828	46.1659	19.8002	-14.8904	-13.1519	131.453	19.8669	14.8904	13.1519	48.5475	19.8669	4.20991	-4.20991	-3.86223	3.86223
4	19.4541	3.24483	51.5202	19.1815	0.98599	5.91142	0.16679	0.83321	198.313	80.7115	-12.2729	-14.6059	130.039	19.0776	12.2729	14.6059	49.9607	19.0776	-15.4391	-11.6087	126.94	19.3165	15.4391	11.6087	53.0603	19.3165	5.00342	-5.00342	-3.98251	3.98251
5	19.0552	3.50829	55.0717	18.7295	0.98291	5.33864	0.18411	0.81589	210.02	79.5645	-11.0941	-14.8434	126.775	18.5313	11.0941	14.8434	53.2252	18.5313	-15.8849	-10.3648	123.124	18.9673	15.8849	10.3648	56.8758	18.9673	5.6571	-5.6571	-3.9561	3.9561
6	18.7776	3.71735	58.1052	18.4059	0.98021	4.95135	0.19797	0.80203	219.292	78.824	-10.1132	-15.0298	123.936	18.1155	10.1132	15.0298	56.0642	18.1155	-16.2523	-9.35425	119.923	18.7521	16.2523	9.3482	60.0929	18.7491	6.18419	-6.18419	-3.85855	3.85855
7	18.5853	3.87566	60.7221	18.1767	0.97802	4.68996	0.20853	0.79147	226.29	78.3437	-9.28652	-15.1691	121.475	17.786	9.28652	15.1691	58.525	17.786	-16.5588	-8.50814	117.195	18.6167	16.5588	8.50814	62.8053	18.6167	6.64029	-6.64029	-3.72171	3.72171
8	18.4476	4.0207	62.9657	18.0041	0.97596	4.47786	0.21795	0.78205	233.019	78.0597	-8.58183	-15.2882	119.307	17.5321	8.58183	15.2882	60.6929	17.5321	-16.8176	-7.80675	114.901	18.5413	16.8176	7.80114	65.115	18.5389	7.00745	-7.00745	-3.57631	3.57631
9	18.3504	4.14289	64.9185	17.8766	0.97418	4.31501	0.22577	0.77423	238.835	77.8979	-7.97381	-15.3894	117.39	17.3325	7.97381	15.3894	62.6097	17.3325	-17.0387	-7.20385	112.918	18.499	17.0387	7.20385	67.0817	18.499	7.31329	-7.31329	-3.41852	3.41852
10	18.283	4.2435	66.6296	17.7837	0.97269	4.1908	0.2321	0.7679	243.737	77.8167	-7.44495	-15.4699	115.699	17.1681	7.44495	15.4699	64.3007	17.1681	-17.2292	-6.68519	111.207	18.4808	17.2293	6.68519	68.7931	18.4808	7.58479	-7.58479	-3.2765	3.2765
11	18.2333	4.34318	68.1264	17.7085	0.97122	4.07731	0.2382	0.7618	248.784	77.8047	-6.98088	-15.536	114.196	17.0323	6.98088	15.536	65.8039	17.0323	-17.3949	-6.23473	109.719	18.4785	17.3949	6.23473	70.2812	18.4785	7.82413	-7.82413	-3.14018	3.14018
12	18.2009	4.42233	69.4603	17.6555	0.97003	3.99236	0.24297	0.75703	252.868	77.8243	-6.56988	-15.5929	112.848	16.9205	6.56988	15.5929	67.1525	16.9205	-17.5404	-5.83962	108.414	18.487	17.5404	5.83962	71.5862	18.487	8.03289	-8.03289	-3.01035	3.01035
13	18.1827	4.47992	70.6549	17.6222	0.96917	3.9336	0.24638	0.75362	255.905	77.86	-6.20434	-15.6444	111.633	16.8298	6.20434	15.6444	68.3675	16.8298	-17.6688	-5.49125	107.265	18.5025	17.6688	5.49125	72.7354	18.5025	8.21217	-8.21217	-2.8879	2.8879
14	18.1651	4.56066	71.7069	17.5833	0.96797	3.85543	0.25107	0.74893	260.265	77.9416	-5.87672	-15.6808	110.545	16.7458	5.87672	15.6808	69.4554	16.7458	-17.783	-5.18158	106.245	18.5225	17.783	5.18158	73.755	18.5225	8.38745	-8.38745	-2.77256	2.77256
15	18.1573	4.61995	72.6614	17.5597	0.96709	3.80084	0.25444	0.74556	263.535	78.0219	-5.58173	-15.7173	109.552	16.679	5.58173	15.7173	70.4483	16.679	-17.885	-4.90485	105.336	18.5454	17.885	4.90485	74.6641	18.5454	8.53477	-8.53477	-2.66425	2.66425
16	18.1583	4.65651	73.534	17.5511	0.96656	3.76916	0.25644	0.74356	265.636	78.0944	-5.31452	-15.7563	108.639	16.6285	5.31452	15.7563	71.3611	16.6285	-17.9768	-4.65593	104.52	18.5699	17.9768	4.65593	75.4796	18.5699	8.65463	-8.65463	-2.56249	2.56249
17	18.1552	4.71803	74.3114	17.5314	0.96564	3.71584	0.25987	0.74013	269.099	78.198	-5.07145	-15.7861	107.81	16.5807	5.07145	15.7861	72.1898	16.5807	-18.0597	-4.43099	103.785	18.5953	18.0597	4.43099	76.2146	18.5953	8.77218	-8.77218	-2.46696	2.46696
18	18.1594	4.7564	75.0288	17.5254	0.96509	3.6846	0.26192	0.73808	271.35	78.2866	-4.8496	-15.8075	107.056	16.5347	4.8496	15.8075	72.9444	16.5347	-18.1347	-4.22693	103.121	18.6208	18.1347	4.22693	76.8795	18.6208	8.88779	-8.88779	-2.37733	2.37733
19	18.1643	4.79568	75.6828	17.5198	0.96452	3.65325	0.26402	0.73598	273.664	78.3797	-4.64629	-15.8215	106.366	16.4896	4.64629	15.8215	73.6341	16.4896	-18.2031	-4.04098	102.516	18.6462	18.2031	4.04098	77.4837	18.6462	9.00176	-9.00176	-2.29314	2.29314
20	18.1694	4.83594	76.2811	17.514	0.96393	3.62164	0.26616	0.73384	276.04	78.476	-4.45934	-15.8433	105.72	16.4589	4.45935	15.8433	74.2799	16.4589	-18.2655	-3.8709	101.965	18.6712	18.2655	3.86742	78.0451	18.6704	9.08949	-9.08949	-2.2201	2.2201
21	18.1745	4.87715	76.8317	17.5079	0.96332	3.58978	0.26835	0.73165	278.47	78.5742	-4.28643	-15.8596	105.124	16.4287	4.28643	15.8596	74.8758	16.4287	-18.3228	-3.71101	101.45	18.6949	18.3228	3.71101	78.5505	18.6949	9.176	-9.176	-2.1452	2.1452
22	18.1863	4.91935	77.3495	17.5083	0.96272	3.55907	0.2705	0.7295	281.062	78.7001	-4.12635	-15.8855	104.561	16.4126	4.12635	15.8855	75.4389	16.4126	-18.3756	-3.5669	100.985	18.7185	18.3756	3.56689	79.0149	18.7185	9.23651	-9.23651	-2.07461	2.07461
23	18.1907	4.93649	77.8171	17.5081	0.96247	3.54667	0.27137	0.72863	282.109	78.7498	-3.97798	-15.8923	104.053	16.3826	3.97798	15.8923	75.9471	16.3826	-18.4241	-3.43075	100.548	18.7408	18.4241	3.43075	79.4517	18.7408	9.32103	-9.32103	-2.01353	2.01353
24	18.1946	4.98034	78.2515	17.4997	0.96181	3.51376	0.27373	0.72627	284.676	78.8493	-3.83955	-15.9099	103.568	16.3666	3.83955	15.9099	76.4322	16.3666	-18.469	-3.30729	100.152	18.7628	18.469	3.30728	79.8475	18.7628	9.37979	-9.37979	-1.95039	1.95039
25	18.2049	4.99873	78.6627	17.5052	0.96156	3.50193	0.27458	0.72542	285.89	78.9243	-3.71092	-15.9237	103.118	16.3504	3.71092	15.9237	76.8817	16.3504	-18.5104	-3.19003	99.7781	18.7833	18.5104	3.19003	80.2219	18.7833	9.4378	-9.4378	-1.89596	1.89596
26	18.2149	5.01759	79.048	17.5102	0.96131	3.48977	0.27547	0.72453	287.126	78.9988	-3.58994	-15.9346	102.696	16.334	3.58994	15.9346	77.3037	16.334	-18.5492	-3.08304	99.4368	18.8036	18.5492	3.08304	80.5632	18.8036	9.4952	-9.4952	-1.83916	1.83916
27	18.2242	5.03702	79.4061	17.5142	0.96104	3.47711	0.27639	0.72361	288.384	79.0717	-3.47706	-15.9424	102.304	16.3172	3.47706	15.9424	77.6964	16.3172	-18.585	-2.98075	99.1118	18.8225	18.585	2.98075	80.8882	18.8225	9.55203	-9.55203	-1.79028	1.79028
28	18.2328	5.05689	79.7415	17.5175	0.96077	3.46409	0.27735	0.72265	289.659	79.1434	-3.37095	-15.9476	101.935	16.2999	3.37095	15.9476	78.0647	16.2999	-18.6184	-2.88762	98.816	18.841	18.6184	2.88762	81.184	18.841	9.60837	-9.60837	-1.73923	1.73923
29	18.2334	5.07725	80.0484	17.5123	0.96045	3.44917	0.27846	0.72154	290.835	79.1853	-3.27078	-15.9504	101.588	16.2823	3.27078	15.9504	78.4116	16.2823	-18.6497	-2.79734	98.5304	18.8584	18.6497	2.79734	81.4696	18.8584	9.66426	-9.66426	-1.69497	1.69497
30	18.2483	5.09808	80.3523	17.5217	0.96018	3.43693	0.27937	0.72063	292.267	79.2827	-3.17669	-15.966	101.253	16.279	3.17669	15.966	78.7471	16.279	-18.6789	-2.71534	98.2711	18.8752	18.6789	2.71534	81.7289	18.8752	9.69468	-9.69468	-1.64872	1.64872
31	18.2551	5.11934	80.6307	17.5226	0.95987	3.42283	0.28043	0.71957	293.595	79.35	-3.08768	-15.9645	100.946	16.2603	3.08768	15.9645	79.0536	16.2603	-18.7063	-2.63547	98.0195	18.891	18.7063	2.63547	81.9806	18.891	9.74982	-9.74982	-1.60874	1.60874
32	18.2612	5.14102	80.892	17.5226	0.95955	3.40838	0.28153	0.71847	294.937	79.4155	-3.00386	-15.9762	100.648	16.2562	3.00386	15.9762	79.3515	16.2562	-18.7317	-2.56043	97.7835	18.9059	18.7317	2.56043	82.2165	18.9059	9.77952	-9.77952	-1.57079	1.57079
33	18.2668	5.16308	81.1406	17.522	0.95922	3.3937	0.28265	0.71735	296.293	79.4802	-2.92387	-15.9711	100.374	16.2366	2.92387	15.9711	79.6256	16.2366	-18.7559	-2.49142	97.5665	18.9207	18.7559	2.49142	82.4335	18.9207	9.83403	-9.83403	-1.53031	1.53031
34	18.2717	5.18555	81.3742	17.5204	0.95888	3.37869	0.2838	0.7162	297.661	79.5428	-2.84847	-15.9796	100.107	16.2315	2.84847	15.9796	79.8928	16.2315	-18.7784	-2.42418	97.3559	18.9343	18.7784	2.42418	82.6441	18.9343	9.86316	-9.86316	-1.49571	1.49571
35	18.276	5.20833	81.5964	17.5181	0.95853	3.36349	0.28498	0.71502	299.04	79.6042	-2.77656	-15.9868	99.8528	16.2261	2.77656	15.9868	80.1472	16.2261	-18.7997	-2.3602	97.1557	18.9473	18.7997	2.3602	82.8443	18.9473	9.89205	-9.89205	-1.46244	1.46244
36	18.2875	5.20394	81.8132	17.5315	0.95866	3.36888	0.28456	0.71544	298.976	79.6391	-2.70852	-15.9924	99.6126	16.2202	2.70852	15.9924	80.3874	16.2202	-18.8196	-2.30202	96.9738	18.9599	18.8196	2.30202	83.0262	18.9599	9.92069	-9.92069	-1.42716	1.42716
37	18.2907	5.22733	82.0128	17.5278	0.95829	3.35311	0.28579	0.71421	300.372	79.6974	-2.6435	-15.9969	99.3834	16.2139	2.6435	15.9969	80.6166	16.2139	-18.8384	-2.24435	96.794	18.9717	18.8384	2.24435	83.206	18.9717	9.94913	-9.94913	-1.39678	1.39678
38	18.2933	5.25103	82.2023	17.5235	0.95792	3.33715	0.28705	0.71295	301.777	79.7542	-2.5816	-16.0002	99.1656	16.2071	2.5816	16.0002	80.8344	16.2071	-18.8561	-2.18955	96.6234	18.9828	18.8561	2.18955	83.3766	18.9828	9.97738	-9.97738	-1.36768	1.36768
39	18.3034	5.24728	82.3896	17.5351	0.95803	3.34175	0.28668	0.71332	301.728	79.7849	-2.52247	-16.0024	98.9579	16.1999	2.52247	16.0024	81.0421	16.1999	-18.8728	-2.1373	96.4611	18.9935	18.8728	2.1373	83.5389	18.9935	10.0054	-10.0054	-1.33972	1.33972
40	18.3049	5.27149	82.5609	17.5294	0.95764	3.32532	0.28798	0.71202	303.145	79.8387	-2.46619	-16.0034	98.7606	16.1923	2.46619	16.0034	81.2394	16.1923	-18.8885	-2.0897	96.3131	19.0038	18.8885	2.0897	83.6869	19.0038	10.0333	-10.0333	-1.31296	1.31296
41	18.314	5.29596	82.7315	17.5316	0.95728	3.31036	0.28918	0.71082	304.704	79.9218	-2.41215	-16.0035	98.5714	16.1843	2.41215	16.0035	81.4285	16.1843	-18.9034	-2.04206	96.1655	19.0134	18.9034	2.04206	83.8345	19.0134	10.0611	-10.0611	-1.28392	1.28392
42	18.3146	5.2928	82.888	17.5332	0.95733	3.31265	0.28899	0.71101	304.533	79.918	-2.36042	-16.0027	98.3907	16.1759	2.36042	16.0027	81.6093	16.1759	-18.9174	-1.99654	96.0247	19.0225	18.9174	1.99654	83.9753	19.0225	10.0887	-10.0887	-1.25914	1.25914
43	18.3148	5.31772	83.0377	17.5258	0.95692	3.29574	0.29035	0.70965	305.969	79.9681	-2.31085	-16.001	98.2178	16.167	2.31085	16.001	81.7822	16.167	-18.9307	-1.95298	95.8901	19.0311	18.9307	1.95298	84.1099	19.0311	10.1162	-10.1162	-1.23527	1.23527
44	18.3226	5.31488	83.1868	17.5348	0.957	3.29919	0.29007	0.70993	305.936	79.9919	-2.26337	-15.9985	98.0524	16.1578	2.26337	15.9985	81.9476	16.1578	-18.9431	-1.91132	95.7615	19.0393	18.9431	1.91132	84.2385	19.0393	10.1435	-10.1435	-1.21231	1.21231
45	18.3219	5.34019	83.3241	17.5264	0.95658	3.28198	0.29146	0.70854	307.381	80.0397	-2.21766	-15.9952	97.8934	16.1482	2.21766	15.9952	82.1066	16.1482	-18.955	-1.87127	95.6381	19.0471	18.955	1.87126	84.3619	19.0471	10.1708	-10.1708	-1.1901	1.1901
46	18.3288	5.33767	83.4604	17.5343	0.95666	3.28501	0.29122	0.70878	307.351	80.0607	-2.17412	-16.007	97.7348	16.1539	2.17412	16.007	82.2652	16.1539	-18.966	-1.835	95.5263	19.0545	18.966	1.835	84.4737	19.0545	10.1727	-10.1727	-1.16601	1.16601
47	18.3272	5.33529	83.5863	17.5334	0.95669	3.28631	0.29111	0.70889	307.187	80.0499	-2.132	-16.0022	97.5889	16.1436	2.132	16.0022	82.4111	16.1436	-18.9764	-1.79816	95.413	19.0614	18.9764	1.79816	84.587	19.0614	10.1997	-10.1997	-1.14544	1.14544
48	18.3334	5.36113	83.7132	17.5321	0.95629	3.27022	0.29242	0.70758	308.781	80.1252	-2.09135	-15.9968	97.4483	16.1329	2.09135	15.9968	82.5517	16.1329	-18.9864	-1.76266	95.304	19.068	18.9864	1.76266	84.696	19.068	10.2267	-10.2267	-1.12551	1.12551
49	18.3392	5.35903	83.8342	17.5387	0.95635	3.27274	0.29222	0.70778	308.756	80.1428	-2.05254	-16.0067	97.3072	16.1377	2.05254	16.0066	82.6928	16.1377	-18.9956	-1.72879	95.2002	19.0741	18.9956	1.72879	84.7998	19.0741	10.2284	-10.2284	-1.10643	1.10643
50	18.3364	5.3852	83.9456	17.5278	0.9559	3.25481	0.29369	0.70631	310.218	80.1847	-2.01494	-15.9999	97.1777	16.1263	2.01494	15.9999	82.8223	16.1263	-19.0043	-1.69603	95.0998	19.0799	19.0043	1.69603	84.9002	19.0799	10.2551	-10.2551	-1.08787	1.08787
51	18.3416	5.38335	84.0583	17.5337	0.95596	3.25703	0.29351	0.70649	310.198	80.2004	-1.97869	-15.9926	97.0531	16.1145	1.97869	15.9926	82.9469	16.1145	-19.0126	-1.66448	95.0033	19.0853	19.0126	1.66448	84.9967	19.0853	10.2818	-10.2818	-1.06992	1.06992
52	18.3463	5.3816	84.1668	17.5393	0.95601	3.25912	0.29333	0.70667	310.178	80.215	-1.94374	-16.0008	96.9262	16.1185	1.94374	16.0008	83.0738	16.1185	-19.0203	-1.63409	94.9104	19.0904	19.0203	1.63409	85.0896	19.0904	10.2832	-10.2832	-1.05256	1.05256
53	18.3424	5.40821	84.2658	17.527	0.95554	3.24081	0.29485	0.70515	311.645	80.2536	-1.91008	-15.9923	96.811	16.106	1.91008	15.9923	83.189	16.106	-19.0275	-1.60485	94.8211	19.095	19.0275	1.60485	85.1789	19.095	10.3097	-10.3097	-1.03578	1.03578
54	18.3465	5.4067	84.3667	17.5318	0.95559	3.2426	0.2947	0.7053	311.628	80.2661	-1.87748	-15.9995	96.6928	16.1093	1.87748	15.9995	83.3072	16.1093	-19.0343	-1.57817	94.7397	19.0996	19.0343	1.57817	85.2603	19.0996	10.3111	-10.3111	-1.01701	1.01701
55	18.3503	5.40529	84.4638	17.5361	0.95563	3.24425	0.29456	0.70544	311.61	80.2774	-1.84605	-15.99	96.5857	16.0962	1.84605	15.99	83.4143	16.0962	-19.0405	-1.5509	94.6566	19.1036	19.0405	1.5509	85.3434	19.1036	10.3374	-10.3374	-1.00131	1.00131
56	18.3453	5.43228	84.5524	17.5226	0.95515	3.22564	0.29611	0.70389	313.082	80.3129	-1.8157	-15.9962	96.4758	16.0989	1.8157	15.9962	83.5242	16.0989	-19.0464	-1.52459	94.5766	19.1073	19.0464	1.52459	85.4234	19.1073	10.3386	-10.3386	-0.9861	0.9861
57	18.3485	5.43109	84.6437	17.5263	0.95519	3.22704	0.296	0.704	313.068	80.3226	-1.78611	-15.9858	96.3753	16.0852	1.78611	15.9858	83.6247	16.0852	-19.0519	-1.49897	94.4987	19.1108	19.0519	1.49897	85.5013	19.1108	10.3649	-10.3649	-0.97122	0.97122
58	18.3514	5.43	84.7314	17.5296	0.95522	3.22829	0.29589	0.70411	313.053	80.3311	-1.75762	-15.9911	96.2723	16.0874	1.75762	15.9911	83.7276	16.0874	-19.057	-1.47432	94.4238	19.1139	19.057	1.47432	85.5762	19.1139	10.366	-10.366	-0.95687	0.95687
59	18.3538	5.42898	84.816	17.5325	0.95525	3.22944	0.2958	0.7042	313.036	80.3384	-1.73009	-15.9796	96.1793	16.073	1.73009	15.9796	83.8207	16.073	-19.0616	-1.45052	94.3516	19.1167	19.0616	1.45052	85.6484	19.1167	10.3921	-10.3921	-0.94297	0.94297
60	18.3477	5.45644	84.8937	17.5176	0.95476	3.21044	0.29739	0.70261	314.515	80.3704	-1.70322	-15.9841	96.0823	16.0746	1.70322	15.9841	83.9177	16.0746	-19.0659	-1.42732	94.2813	19.1193	19.0659	1.42732	85.7187	19.1193	10.3931	-10.3931	-0.92936	0.92936
61	18.3496	5.45563	84.9731	17.5198	0.95478	3.21132	0.29732	0.70268	314.5	80.3758	-1.67735	-15.9718	95.9952	16.0597	1.67735	15.9718	84.0048	16.0597	-19.0698	-1.40499	94.2137	19.1215	19.0698	1.40499	85.7863	19.1215	10.4191	-10.4191	-0.91624	0.91624
62	18.3512	5.4549	85.0504	17.5217	0.9548	3.21211	0.29725	0.70275	314.486	80.3806	-1.6521	-15.9755	95.9042	16.0607	1.6521	15.9755	84.0958	16.0607	-19.0734	-1.38323	94.1479	19.1235	19.0734	1.38323	85.8521	19.1235	10.42	-10.42	-0.9034	0.9034
63	18.3524	5.45426	85.1245	17.5232	0.95482	3.21276	0.2972	0.7028	314.47	80.384	-1.62786	-15.9623	95.823	16.0451	1.62787	15.9623	84.177	16.0451	-19.0765	-1.36234	94.0848	19.1251	19.0765	1.36234	85.9152	19.1251	10.4459	-10.4459	-0.89105	0.89105
64	18.3451	5.48212	85.1931	17.5069	0.95431	3.19345	0.29883	0.70117	315.951	80.4125	-1.60392	-15.9653	95.7368	16.0457	1.60392	15.9653	84.2632	16.0457	-19.0794	-1.34172	94.0226	19.1266	19.0795	1.34172	85.9774	19.1266	10.4467	-10.4467	-0.87882	0.87882
65	18.3458	5.48168	85.263	17.5077	0.95432	3.19386	0.2988	0.7012	315.937	80.4141	-1.58102	-15.9513	95.6604	16.0295	1.58102	15.9513	84.3396	16.0295	-19.0819	-1.32202	93.9632	19.1276	19.0819	1.32202	86.0368	19.1276	10.4726	-10.4726	-0.86711	0.86711
66	18.3463	5.48132	85.3313	17.5083	0.95433	3.19418	0.29877	0.70123	315.924	80.4152	-1.55859	-15.9534	95.5799	16.0294	1.55859	15.9534	84.4201	16.0294	-19.0841	-1.30274	93.9051	19.1285	19.0841	1.30274	86.0949	19.1285	10.4733	-10.4733	-0.85561	0.85561
67	18.3464	5.48103	85.3972	17.5085	0.95433	3.19439	0.29875	0.70125	315.909	80.415	-1.53691	-15.9551	95.5022	16.029	1.53691	15.9551	84.4978	16.029	-19.0859	-1.28412	93.8491	19.129	19.0859	1.28412	86.1509	19.129	10.4739	-10.4739	-0.84448	0.84448
68	18.3462	5.48083	85.4614	17.5084	0.95433	3.19449	0.29874	0.70126	315.895	80.4141	-1.51577	-15.94	95.432	16.0119	1.51577	15.94	84.568	16.0119	-19.0873	-1.26596	93.7946	19.1293	19.0873	1.26596	86.2054	19.1293	10.4996	-10.4996	-0.8336	0.8336
69	18.3458	5.50917	85.5239	17.4991	0.95385	3.17636	0.3003	0.6997	317.522	80.4698	-1.49516	-15.9411	95.3583	16.011	1.49516	15.9411	84.6417	16.011	-19.0885	-1.24828	93.7415	19.1293	19.0885	1.24828	86.2585	19.1293	10.5002	-10.5002	-0.82298	0.82298
70	18.3366	5.50915	85.5804	17.4894	0.9538	3.17461	0.30045	0.69955	317.36	80.4347	-1.47515	-15.9417	95.2868	16.0098	1.47516	15.9417	84.7132	16.0098	-19.0894	-1.23113	93.6901	19.129	19.0894	1.23113	86.3099	19.129	10.5008	-10.5008	-0.81265	0.81265
71	18.3356	5.5092	85.6396	17.4884	0.95379	3.17439	0.30046	0.69954	317.346	80.4311	-1.45559	-15.9255	95.2223	15.9919	1.45559	15.9255	84.7777	15.9919	-19.09	-1.21436	93.6398	19.1285	19.09	1.21436	86.3602	19.1285	10.5264	-10.5264	-0.80254	0.80254
72	18.3343	5.50933	85.6972	17.487	0.95378	3.17407	0.30049	0.69951	317.331	80.4266	-1.43654	-15.9255	95.1543	15.9901	1.43654	15.9255	84.8457	15.9901	-19.0902	-1.19805	93.591	19.1278	19.0902	1.19804	86.409	19.1278	10.5269	-10.5269	-0.79267	0.79267
73	18.3326	5.50957	85.7527	17.4851	0.95377	3.1736	0.30053	0.69947	317.316	80.4208	-1.41811	-15.925	95.0887	15.988	1.41811	15.925	84.9113	15.988	-19.0901	-1.18228	93.5439	19.1266	19.0901	1.18228	86.4561	19.1266	10.5273	-10.5273	-0.78312	0.78312
74	18.3221	5.50987	85.8029	17.474	0.95371	3.1714	0.30072	0.69928	317.151	80.3816	-1.40013	-15.9076	95.03	15.9691	1.40013	15.9076	84.97	15.9691	-19.0896	-1.16689	93.498	19.1252	19.0896	1.16689	86.502	19.1252	10.5528	-10.5528	-0.77378	0.77378
75	18.3199	5.53874	85.8559	17.4626	0.9532	3.1528	0.30233	0.69767	318.775	80.4317	-1.38253	-15.9065	94.9674	15.9664	1.38253	15.9065	85.0326	15.9664	-19.0889	-1.15184	93.4531	19.1236	19.0889	1.15184	86.5469	19.1236	10.5532	-10.5532	-0.76462	0.76462
76	18.3174	5.53922	85.9075	17.4597	0.95318	3.15202	0.3024	0.6976	318.758	80.4232	-1.36537	-15.905	94.9065	15.9635	1.36537	15.905	85.0935	15.9635	-19.0878	-1.13717	93.4094	19.1217	19.0878	1.13717	86.5906	19.1217	10.5535	-10.5535	-0.75568	0.75568
77	18.3145	5.53978	85.9577	17.4566	0.95316	3.15113	0.30248	0.69752	318.741	80.4135	-1.34863	-15.8865	94.8523	15.9436	1.34863	15.8865	85.1477	15.9436	-19.0864	-1.12288	93.3669	19.1194	19.0864	1.12288	86.6331	19.1194	10.5788	-10.5788	-0.74695	0.74695
78	18.3113	5.54044	86.0065	17.453	0.95313	3.15012	0.30257	0.69743	318.723	80.4028	-1.33232	-15.8842	94.7946	15.94	1.33232	15.8842	85.2054	15.94	-19.0847	-1.10896	93.3256	19.1169	19.0847	1.10896	86.6744	19.1169	10.5791	-10.5791	-0.73844	0.73844
79	18.3078	5.54119	86.0541	17.4491	0.9531	3.14898	0.30267	0.69733	318.705	80.391	-1.31641	-15.8815	94.7384	15.936	1.31641	15.8815	85.2616	15.936	-19.0826	-1.09538	93.2853	19.1141	19.0826	1.09538	86.7147	19.1141	10.5794	-10.5794	-0.73012	0.73012
80	18.304	5.54203	86.1005	17.4448	0.95306	3.14773	0.30278	0.69722	318.686	80.3782	-1.30085	-15.8619	94.6884	15.9151	1.30085	15.8619	85.3116	15.9151	-19.0803	-1.08211	93.246	19.1109	19.0803	1.08211	86.754	19.1109	10.6046	-10.6046	-0.72198	0.72198
81	18.2998	5.54295	86.1457	17.4401	0.95302	3.14636	0.3029	0.6971	318.666	80.3642	-1.28566	-15.8585	94.6349	15.9105	1.28566	15.8585	85.3651	15.9105	-19.0775	-1.06916	93.2077	19.1075	19.0775	1.06916	86.7923	19.1075	10.6048	-10.6048	-0.71401	0.71401
82	18.2951	5.54397	86.1894	17.4349	0.95298	3.14483	0.30303	0.69697	318.644	80.3487	-1.27096	-15.8545	94.5832	15.9054	1.27096	15.8545	85.4168	15.9054	-19.0744	-1.05663	93.1707	19.1036	19.0744	1.05663	86.8293	19.1036	10.6049	-10.6049	-0.70631	0.70631
83	18.2903	5.54509	86.2327	17.4295	0.95294	3.14322	0.30317	0.69683	318.624	80.3328	-1.25636	-15.8504	94.532	15.9001	1.25636	15.8504	85.468	15.9001	-19.071	-1.04419	93.134	19.0996	19.071	1.04419	86.866	19.0996	10.6051	-10.6051	-0.69864	0.69864
84	18.2763	5.57479	86.271	17.4053	0.95234	3.12214	0.30503	0.69497	320.086	80.3404	-1.24223	-15.8291	94.4873	15.8777	1.24223	15.8291	85.5127	15.8777	-19.0672	-1.03216	93.0986	19.0951	19.0672	1.03108	86.9047	19.095	10.6301	-10.6301	-0.69121	0.69121
85	18.2705	5.57611	86.3116	17.3988	0.95229	3.12024	0.3052	0.6948	320.061	80.3214	-1.22848	-15.8239	94.4392	15.8715	1.22848	15.8239	85.5608	15.8715	-19.0629	-1.01938	93.0609	19.0902	19.0629	1.01938	86.9391	19.0902	10.6302	-10.6302	-0.68558	0.68558
86	18.2645	5.57754	86.3517	17.392	0.95223	3.11823	0.30538	0.69462	320.037	80.3015	-1.21488	-15.8184	94.3918	15.865	1.21488	15.8184	85.6082	15.865	-19.0585	-1.00782	93.027	19.0851	19.0584	1.00782	86.973	19.0851	10.6302	-10.6302	-0.6784	0.6784
87	18.258	5.57907	86.3908	17.3847	0.95217	3.11606	0.30557	0.69443	320.011	80.2802	-1.20161	-15.8125	94.3456	15.8581	1.20161	15.8125	85.6544	15.8581	-19.0535	-0.99655	92.994	19.0796	19.0535	0.99655	87.006	19.0796	10.6302	-10.6302	-0.67139	0.67139
88	18.2511	5.58071	86.429	17.377	0.9521	3.11376	0.30577	0.69423	319.984	80.2575	-1.18861	-15.7894	94.3051	15.8341	1.18861	15.7894	85.6949	15.8341	-19.0482	-0.98551	92.9617	19.0737	19.0482	0.98551	87.0383	19.0737	10.655	-10.655	-0.66451	0.66451

**inter disk fault**	1. General ellipse parameter features										2. features extracted from locus																			
faulty disk	A'	B'	θ	f	e	e'	$\frac{e}{e'}$	g	$A_{ellipse}$	$C_{ellipse}$	I_{min}	ΔV_{Imin}	θ_{Imin}	Abs_{Imin}	I_{max}	ΔV_{Imax}	θ_{Imax}	Abs_{Imax}	ΔV_{min}	$I_{\Delta Vmin}$	$\theta_{\Delta Vmin}$	$Abs_{\Delta Vmin}$	Δv_{max}	$I_{\Delta Vmax}$	$\theta_{\Delta Vmax}$	$Abs_{\Delta Vmax}$	$\Delta V_{H(0)}$	$\Delta V_{L(0)}$	$I_{H(0)}$	$I_{L(0)}$
1	11.7564	3.63535	86.4001	11.1802	0.95099	3.07541	0.30922	0.69078	134.267	51.7804	-0.77234	-10.1476	94.3524	10.177	0.77234	10.1476	85.6476	10.177	-12.2814	-0.63807	92.9741	12.2979	12.2813	0.63807	87.0259	12.2979	6.91787	-6.91787	-0.43517	0.43517
2	11.7124	3.64759	86.3727	11.1299	0.95027	3.05131	0.31143	0.68857	134.215	51.6398	-0.7761	-10.08	94.4027	10.1098	0.7761	10.08	85.5972	10.1098	-12.2427	-0.63831	92.9846	12.2593	12.2427	0.63901	87.0122	12.2593	6.94805	-6.94805	-0.44044	0.44044
3	11.6632	3.67867	86.3421	11.0679	0.94896	3.00866	0.31541	0.68459	134.79	51.5188	-0.77981	-10.0126	94.4534	10.0429	0.77981	10.0126	85.5466	10.0429	-12.2047	-0.63984	93.001	12.2215	12.2047	0.63984	86.999	12.2215	6.97872	-6.97872	-0.44677	0.44677
4	11.6195	3.69203	86.3148	11.0173	0.94818	2.98409	0.31774	0.68226	134.773	51.3821	-0.78352	-9.93374	94.5098	9.9646	0.78352	9.93375	85.4902	9.9646	-12.1668	-0.63921	93.0074	12.1835	12.1668	0.63921	86.9926	12.1835	7.025	-7.025	-0.45211	0.45211
5	11.5749	3.70563	86.2868	10.9658	0.94737	2.95922	0.32014	0.67986	134.751	51.2428	-0.78731	-9.86484	94.5631	9.8962	0.78731	9.86484	85.4369	9.8962	-12.1281	-0.64071	93.024	12.145	12.1281	0.64071	86.976	12.145	7.0552	-7.0552	-0.45753	0.45753
6	11.5245	3.71974	86.2549	10.9077	0.94648	2.93239	0.32277	0.67723	134.674	51.0828	-0.7911	-9.79562	94.6172	9.8275	0.7911	9.79561	85.3828	9.8275	-12.0896	-0.64072	93.0337	12.1066	12.0896	0.64072	86.9663	12.1066	7.08551	-7.08551	-0.46402	0.46402
7	11.4799	3.75235	86.2267	10.8493	0.94507	2.89134	0.32686	0.67314	135.329	50.9836	-0.79489	-9.72615	94.6722	9.75858	0.79489	9.72616	85.3278	9.75858	-12.0514	-0.64139	93.0465	12.0684	12.0514	0.64065	86.957	12.0684	7.11602	-7.11602	-0.46955	0.46955
8	11.4344	3.76729	86.1977	10.796	0.94417	2.86572	0.32947	0.67053	135.33	50.8447	-0.79874	-9.64449	94.7344	9.67749	0.79874	9.64447	85.2656	9.67749	-12.0126	-0.64132	93.056	12.0297	12.0125	0.64206	86.9405	12.0297	7.16134	-7.16134	-0.47614	0.47614
9	11.3829	3.8005	86.1645	10.7297	0.94262	2.82324	0.33388	0.66612	135.907	50.7218	-0.80262	-9.57356	94.7923	9.60715	0.80262	9.57356	85.2077	9.60715	-11.9737	-0.64194	93.0688	11.9909	11.9737	0.64194	86.9312	11.9909	7.19144	-7.19144	-0.48179	0.48179
10	11.3424	3.81619	86.1387	10.6812	0.9417	2.79891	0.33645	0.66355	135.984	50.6037	-0.80657	-9.50169	94.852	9.53583	0.80657	9.50166	85.1479	9.53583	-11.9343	-0.64102	93.0745	11.9515	11.9344	0.64179	86.9218	11.9516	7.22126	-7.22126	-0.48853	0.48853
11	11.2845	3.85026	86.1008	10.6073	0.93999	2.75495	0.3412	0.6588	136.496	50.4598	-0.81046	-9.41847	94.9182	9.45327	0.81046	9.41846	85.0818	9.45327	-11.8957	-0.6423	93.0906	11.913	11.8957	0.64152	86.9131	11.9129	7.26633	-7.26633	-0.49427	0.49427
12	11.2374	3.86698	86.0701	10.5511	0.93893	2.7285	0.34412	0.65588	136.517	50.3197	-0.81446	-9.34544	94.9808	9.38083	0.81447	9.3454	85.0192	9.38083	-11.8562	-0.64125	93.0959	11.8735	11.8562	0.64283	86.8965	11.8736	7.29597	-7.29597	-0.50114	0.50114
13	11.1847	3.88443	86.0353	10.4885	0.93775	2.70013	0.3473	0.6527	136.49	50.1605	-0.81842	-9.27243	95.044	9.30846	0.81842	9.27241	84.9559	9.30846	-11.8174	-0.64166	93.108	11.8348	11.8174	0.64246	86.8881	11.8348	7.32615	-7.32615	-0.50801	0.50801
14	11.1373	3.91964	86.0042	10.4247	0.93602	2.65962	0.35194	0.64806	137.144	50.0602	-0.82242	-9.18702	95.1155	9.22377	0.82242	9.18703	84.8845	9.22377	-11.7782	-0.64205	93.1202	11.7957	11.7782	0.64124	86.8837	11.7957	7.37054	-7.37054	-0.51395	0.51395
15	11.0899	3.93808	85.973	10.3671	0.93483	2.63252	0.35511	0.64489	137.202	49.9237	-0.82643	-9.11275	95.182	9.15018	0.82643	9.11278	84.8181	9.15018	-11.7393	-0.64074	93.1242	11.7567	11.7393	0.64074	86.8758	11.7567	7.40053	-7.40053	-0.52095	0.52095
16	11.0414	3.95685	85.9407	10.308	0.93358	2.60511	0.35837	0.64163	137.253	49.7843	-0.83055	-9.02561	95.2576	9.06374	0.83055	9.02561	84.7424	9.06374	-11.6995	-0.6419	93.1404	11.7171	11.6995	0.64107	86.8636	11.7171	7.44427	-7.44427	-0.52806	0.52806
17	10.9931	3.97625	85.9086	10.2488	0.93229	2.57749	0.36171	0.63829	137.323	49.6473	-0.83464	-8.94993	95.3278	8.98872	0.83464	8.94989	84.6722	8.98872	-11.6603	-0.6413	93.148	11.6779	11.6603	0.64046	86.8561	11.6778	7.47404	-7.47404	-0.53519	0.53519
18	10.9381	4.01336	85.8712	10.1752	0.93025	2.53533	0.36692	0.63308	137.911	49.525	-0.83876	-8.86171	95.4069	8.90132	0.83876	8.86171	84.5931	8.90132	-11.6209	-0.6398	93.1513	11.6385	11.6209	0.6398	86.8487	11.6385	7.51769	-7.51769	-0.54239	0.54239
19	10.8891	4.03394	85.8382	10.1143	0.92885	2.5073	0.37046	0.62954	137.997	49.3888	-0.84291	-8.78475	95.4809	8.82507	0.84291	8.78472	84.5191	8.82507	-11.5816	-0.63906	93.1583	11.5992	11.5816	0.63906	86.8417	11.5992	7.54738	-7.54738	-0.54964	0.54964
20	10.8338	4.05522	85.8002	10.0462	0.9273	2.47735	0.37431	0.62569	138.021	49.2313	-0.84706	-8.69552	95.5638	8.73667	0.84705	8.69551	84.4362	8.73667	-11.5428	-0.63822	93.1648	11.5604	11.5428	0.63822	86.8353	11.5604	7.59099	-7.59099	-0.55694	0.55694
21	10.7836	4.07686	85.7659	9.98321	0.92578	2.44875	0.37806	0.62194	138.114	49.0937	-0.8513	-8.61683	95.6422	8.65878	0.8513	8.61683	84.3577	8.65878	-11.503	-0.63648	93.167	11.5206	11.503	0.63648	86.8329	11.5206	7.6203	-7.6203	-0.56434	0.56434
22	10.7338	4.11607	85.7318	9.91322	0.92355	2.40842	0.38347	0.61653	138.799	48.9979	-0.85552	-8.52609	95.7299	8.56893	0.85552	8.52611	84.2701	8.56893	-11.464	-0.63549	93.1729	11.4816	11.464	0.63639	86.8227	11.4817	7.66356	-7.66356	-0.57177	0.57177
23	10.6834	4.13906	85.6972	9.84905	0.9219	2.37954	0.38743	0.61257	138.919	48.8639	-0.85978	-8.43441	95.8205	8.47813	0.85978	8.43443	84.1796	8.47813	-11.4249	-0.63535	93.183	11.4425	11.4249	0.63535	86.817	11.4425	7.70642	-7.70642	-0.57926	0.57926
24	10.6325	4.16262	85.6619	9.78382	0.92018	2.3504	0.3915	0.6085	139.044	48.7296	-0.86409	-8.35395	95.9054	8.39852	0.86409	8.35395	84.0946	8.39852	-11.3856	-0.63333	93.1838	11.4032	11.3856	0.63333	86.8162	11.4032	7.73582	-7.73582	-0.58784	0.58784
25	10.575	4.20332	85.621	9.70371	0.91761	2.30858	0.39748	0.60252	139.644	48.6106	-0.86842	-8.26078	96.0012	8.30628	0.86842	8.26076	83.9988	8.30628	-11.3465	-0.63306	93.1934	11.3642	11.3465	0.63213	86.8113	11.3641	7.77839	-7.77839	-0.59546	0.59546
26	10.524	4.22834	85.5854	9.63719	0.91574	2.27919	0.40178	0.59822	139.798	48.4805	-0.87272	-8.16718	96.0993	8.21369	0.87272	8.16719	83.9007	8.21369	-11.308	-0.62985	93.188	11.3255	11.308	0.62985	86.812	11.3256	7.82104	-7.82104	-0.60311	0.60311
27	10.4725	4.25402	85.5493	9.56961	0.91378	2.24954	0.40621	0.59379	139.959	48.3507	-0.87708	-8.0727	96.2008	8.13248	0.87708	8.08505	83.8086	8.13248	-11.2695	-0.62941	93.1967	11.287	11.2695	0.62845	86.8082	11.287	7.85071	-7.85071	-0.61182	0.61182
28	10.4271	4.28033	85.5183	9.5081	0.91186	2.22135	0.4105	0.5895	140.214	48.2451	-0.88149	-7.98959	96.296	8.03812	0.88149	7.98964	83.7041	8.03812	-11.2308	-0.626	93.1903	11.2482	11.2308	0.626	86.8097	11.2483	7.89284	-7.89284	-0.61962	0.61962
29	10.3616	4.30741	85.4695	9.42383	0.9095	2.18782	0.41571	0.58429	140.214	48.0678	-0.88592	-7.8935	96.4038	7.94306	0.88592	7.8935	83.5962	7.94306	-11.1924	-0.62442	93.1932	11.2098	11.1924	0.62442	86.8068	11.2098	7.93488	-7.93488	-0.62846	0.62846
30	10.3089	4.35116	85.4318	9.34567	0.90656	2.14786	0.42208	0.57792	140.919	47.9777	-0.89039	-7.79655	96.5151	7.84724	0.89039	7.79657	83.4849	7.84724	-11.1541	-0.62175	93.1905	11.1714	11.1541	0.62175	86.8095	11.1714	7.97668	-7.97668	-0.63638	0.63638
31	10.2633	4.36384	85.4004	9.28937	0.9051	2.12871	0.42519	0.57481	140.704	47.8407	-0.89482	-7.69929	96.6293	7.75109	0.89482	7.69926	83.3707	7.75109	-11.1166	-0.62095	93.1971	11.1339	11.1166	0.61993	86.8081	11.1338	8.01871	-8.01871	-0.64528	0.64528
32	10.2034	4.40899	85.3559	9.20159	0.90182	2.08701	0.43211	0.56789	141.329	47.7287	-0.89934	-7.60078	96.748	7.65381	0.89934	7.60079	83.252	7.65381	-11.0788	-0.61704	93.1878	11.0959	11.0788	0.61806	86.8069	11.096	8.06024	-8.06024	-0.65428	0.65428
33	10.157	4.4233	85.3236	9.14322	0.90019	2.06706	0.43549	0.56451	141.143	47.5936	-0.90387	-7.50164	96.8704	7.5559	0.90387	7.50164	83.1296	7.5559	-11.0414	-0.614	93.1829	11.0584	11.0414	0.61399	86.8171	11.0584	8.10169	-8.10169	-0.66235	0.66235
34	10.1035	4.46993	85.2847	9.06097	0.89681	2.02709	0.44241	0.55759	141.881	47.5106	-0.90842	-7.40182	96.9969	7.45734	0.90842	7.4018	83.0031	7.45734	-11.0044	-0.61083	93.1771	11.0214	11.0045	0.61083	86.8229	11.0214	8.14316	-8.14316	-0.67144	0.67144
35	10.0423	4.48594	85.2383	8.9847	0.89468	2.00286	0.4467	0.5533	141.527	47.3265	-0.91305	-7.30064	97.1286	7.37028	0.91305	7.31351	82.8838	7.37028	-10.9671	-0.60756	93.1709	10.984	10.9671	0.60756	86.8291	10.984	8.18405	-8.18405	-0.6806	0.6806
36	9.99587	4.53416	85.206	8.90835	0.8912	1.96472	0.4536	0.5464	142.386	47.2744	-0.91759	-7.19966	97.2631	7.25788	0.91759	7.19964	82.7369	7.25788	-10.9313	-0.60518	93.1687	10.9481	10.9313	0.60517	86.8313	10.9481	8.22552	-8.22552	-0.68974	0.68974
37	9.94077	4.55196	85.165	8.83735	0.889	1.94144	0.45791	0.54209	142.157	47.1178	-0.92231	-7.09663	97.4049	7.15631	0.92232	7.09662	82.595	7.15631	-10.8945	-0.60168	93.1611	10.9111	10.8944	0.60169	86.8388	10.911	8.26597	-8.26597	-0.69903	0.69903
38	9.88662	4.6017	85.125	8.75041	0.88508	1.90156	0.46545	0.53455	142.928	47.0433	-0.92695	-6.99377	97.5499	7.05493	0.92695	6.99376	82.4501	7.05493	-10.8591	-0.59798	93.1519	10.8756	10.8592	0.59686	86.854	10.8755	8.30712	-8.30712	-0.70921	0.70921
39	9.83207	4.62162	85.0844	8.67814	0.88264	1.87773	0.47006	0.52994	142.754	46.895	-0.93161	-6.88996	97.7004	6.95269	0.93161	6.89	82.2996	6.95269	-10.8241	-0.593	93.1358	10.8403	10.8241	0.59413	86.8582	10.8404	8.34801	-8.34801	-0.7185	0.7185
40	9.77711	4.65775	85.0432	8.59635	0.87923	1.8456	0.47639	0.52361	143.066	46.7858	-0.93633	-6.78519	97.857	6.84949	0.93633	6.78519	82.143	6.84949	-10.7893	-0.58902	93.1248	10.8054	10.7893	0.59016	86.8691	10.8055	8.38873	-8.38873	-0.72786	0.72786
41	9.70708	4.69474	84.9863	8.49628	0.87527	1.80975	0.48364	0.51636	143.169	46.6253	-0.9411	-6.67935	98.02	6.74529	0.9411	6.67932	81.9799	6.74529	-10.7547	-0.58491	93.113	10.7706	10.7548	0.58491	86.887	10.7707	8.42918	-8.42918	-0.73726	0.73726
42	9.66693	4.73284	84.9603	8.4291	0.87195	1.78098	0.48959	0.51041	143.734	46.5759	-0.94581	-6.57351	98.1876	6.6412	0.9458	6.57351	81.8124	6.6412	-10.7217	-0.57941	93.0933	10.7373	10.7217	0.57941	86.9067	10.7373	8.47015	-8.47015	-0.74755	0.74755
43	9.61081	4.77161	84.9175	8.34263	0.86805	1.74839	0.49648	0.50352	144.071	46.4717	-0.95065	-6.46584	98.3641	6.53534	0.95065	6.46582	81.6359	6.53534	-10.688	-0.57381	93.0731	10.7033	10.688	0.575	86.9205	10.7034	8.5103	-8.5103	-0.75704	0.75704
44	9.54752	4.81151	84.8668	8.24649	0.86373	1.71391	0.50395	0.49605	144.318	46.3455	-0.95548	-6.34422	98.5648	6.41574	0.95548	6.34419	81.4352	6.41574	-10.6553	-0.56799	93.0513	10.6704	10.6553	0.57041	86.9357	10.6705	8.56071	-8.56071	-0.76744	0.76744
45	9.49944	4.85245	84.8325	8.16659	0.85969	1.68298	0.51081	0.48919	144.814	46.2774	-0.96028	-6.23562	98.7548	6.30911	0.96028	6.2356	81.2452	6.30911	-10.6237	-0.56318	93.0345	10.6387	10.6237	0.5644	86.9589	10.6387	8.60122	-8.60122	-0.7778	0.7778
46	9.45103	4.87977	84.7979	8.09382	0.8564	1.65865	0.51632	0.48368	144.887	46.1741	-0.96514	-6.1259	98.9534	6.20144	0.96514	6.12587	81.0466	6.20144	-10.5926	-0.55823	93.0167	10.6073	10.5926	0.55699	86.99	10.6072	8.64155	-8.64155	-0.78732	0.78732
47	9.38826	4.92293	84.747	7.99401	0.85149	1.62383	0.52437	0.47563	145.197	46.0609	-0.96994	-6.00241	99.1792	6.08028	0.96994	6.00241	80.8208	6.08028	-10.563	-0.55307	92.9972	10.5775	10.563	0.55305	87.0029	10.5775	8.69187	-8.69187	-0.79768	0.79768
48	9.33923	4.96687	84.7118	7.90894	0.84685	1.59234	0.53183	0.46817	145.728	45.9997	-0.97488	-5.89056	99.3972	5.9707	0.97488	5.89057	80.6028	5.9707	-10.5328	-0.54527	92.9635	10.5469	10.5329	0.54526	87.0366	10.547	8.73165	-8.73165	-0.80813	0.80813
49	9.28257	5.01191	84.6676	7.81325	0.84171	1.55894	0.53993	0.46007	146.157	45.9152	-0.97986	-5.7642	99.6475	5.84687	0.97986	5.76418	80.3525	5.84687	-10.5036	-0.53853	92.935	10.5174	10.5036	0.53724	87.072	10.5173	8.78061	-8.78061	-0.8186	0.8186
50	9.22652	5.04388	84.6242	7.7258	0.83735	1.53172	0.54667	0.45333	146.202	45.7999	-0.98477	-5.65128	99.8849	5.73644	0.98477	5.65128	80.1152	5.73644	-10.4759	-0.53154	92.9046	10.4893	10.4759	0.53153	87.0954	10.4893	8.8208	-8.8208	-0.829	0.829
51	9.1863	5.09132	84.5994	7.64634	0.83236	1.50184	0.55423	0.44577	146.933	45.7817	-0.98969	-5.52373	100.158	5.6117	0.98968	5.52374	79.8422	5.6117	-10.4492	-0.52434	92.8727	10.4623	10.4492	0.52564	87.1202	10.4625	8.86989	-8.86989	-0.83938	0.83938
52	9.1295	5.12561	84.5549	7.55485	0.82752	1.47394	0.56143	0.43857	147.009	45.6714	-0.99473	-5.40846	100.421	5.49918	0.99473	5.40846	79.5786	5.49918	-10.4226	-0.51564	92.8323	10.4353	10.4226	0.51431	87.175	10.4353	8.90948	-8.90948	-0.85064	0.85064
53	9.06529	5.18931	84.5013	7.43307	0.81995	1.43238	0.57244	0.42756	147.789	45.6138	-0.99973	-5.27875	100.724	5.37259	0.99973	5.27876	79.276	5.37259	-10.3975	-0.50667	92.7898	10.4099	10.3975	0.50803	87.2027	10.4099	8.9579	-8.9579	-0.86102	0.86102
54	9.01628	5.2121	84.4662	7.35713	0.81598	1.41155	0.57808	0.42192	147.635	45.5022	-1.0048	-5.16165	101.016	5.25857	1.00481	5.16168	78.9842	5.25857	-10.3729	-0.50022	92.7609	10.3849	10.3728	0.50024	87.239	10.3849	8.99744	-8.99744	-0.87144	0.87144
55	8.96842	5.26426	84.4326	7.26087	0.8096	1.37928	0.58698	0.41302	148.321	45.4737	-1.00982	-5.0303	101.351	5.13064	1.00982	5.03028	78.6488	5.13064	-10.3502	-0.49216	92.7224	10.3619	10.3502	0.49078	87.2852	10.3618	9.04555	-9.04555	-0.88254	0.88254
56	8.92072	5.31759	84.3995	7.16257	0.80291	1.34696	0.59609	0.40391	149.027	45.45	-1.01484	-4.89793	101.706	5.00196	1.01484	4.89793	78.2941	5.00196	-10.3286	-0.48107	92.6667	10.3398	10.3286	0.48107	87.3333	10.3398	9.09342	-9.09342	-0.89281	0.89281
57	8.86444	5.37198	84.3554	7.05125	0.79545	1.3126	0.60601	0.39399	149.601	45.4005	-1.01993	-4.77855	102.048	4.88619	1.01994	4.77856	77.9516	4.88619	-10.3078	-0.47114	92.617	10.3186	10.3078	0.47115	87.3829	10.3185	9.13325	-9.13325	-0.90385	0.90385
58	8.80883	5.40061	84.312	6.95909	0.79001	1.28857	0.61309	0.38691	149.455	45.2846	-1.02499	-4.6444	102.445	4.75615	1.02499	4.64439	77.5546	4.75615	-10.2887	-0.46237	92.5731	10.2991	10.2888	0.46237	87.4269	10.2991	9.18084	-9.18084	-0.91478	0.91478
59	8.76075	5.47101	84.2787	6.84243	0.78103	1.25067	0.62449	0.37551	150.577	45.3097	-1.03015	-4.5085	102.871	4.62468	1.03015	4.50849	77.1293	4.62468	-10.2701	-0.4534	92.5278	10.2801	10.2701	0.45196	87.4802	10.28	9.22755	-9.22755	-0.92572	0.92572
60	8.70558	5.51599	84.2359	6.73506	0.77365	1.22101	0.63362	0.36638	150.859	45.242	-1.03521	-4.37254	103.32	4.49341	1.03521	4.37254	76.6803	4.49341	-10.2539	-0.44122	92.4639	10.2634	10.2539	0.44269	87.5279	10.2634	9.27485	-9.27485	-0.93648	0.93648
61	8.6503	5.57557	84.1929	6.61367	0.76456	1.18619	0.64455	0.35545	151.52	45.2153	-1.04032	-4.23538	103.8	4.36127	1.04032	4.23538	76.1999	4.36127	-10.2388	-0.43173	92.4145	10.2479	10.2388	0.43023	87.5939	10.2479	9.32172	-9.32172	-0.94718	0.94718
62	8.60313	5.60988	84.1611	6.5225	0.75815	1.16268	0.65207	0.34793	151.621	45.1479	-1.04551	-4.11143	104.268	4.22802	1.04551	4.09672	75.6833	4.22802	-10.2245	-0.41751	92.3383	10.233	10.2245	0.42053	87.6448	10.2331	9.36146	-9.36146	-0.95787	0.95787
63	8.54853	5.67228	84.119	6.39551	0.74814	1.1275	0.66354	0.33646	152.335	45.134	-1.05061	-3.97264	104.813	4.10923	1.05061	3.97265	75.1866	4.10923	-10.2126	-0.40597	92.2764	10.2207	10.2126	0.40597	87.7236	10.2207	9.40828	-9.40828	-0.96837	0.96837
64	8.50191	5.74883	84.0882	6.26366	0.73674	1.08955	0.67618	0.32382	153.548	45.1887	-1.05583	-3.83203	105.404	3.97482	1.05584	3.83202	74.5955	3.97482	-10.2014	-0.39574	92.2215	10.2091	10.2013	0.39575	87.7784	10.209	9.4543	-9.4543	-0.97887	0.97887
65	8.44846	5.80083	84.0478	6.14221	0.72702	1.05885	0.68661	0.31339	153.963	45.1527	-1.0609	-3.69176	106.033	3.8412	1.0609	3.69179	73.9671	3.8412	-10.1932	-0.38521	92.1642	10.2004	10.1932	0.38364	87.8446	10.2004	9.50109	-9.50109	-0.9891	0.9891
66	8.40302	5.84128	84.019	6.04071	0.71887	1.03414	0.69514	0.30486	154.203	45.1123	-1.06611	-3.53457	106.785	3.69185	1.0661	3.53457	73.2155	3.69185	-10.1856	-0.36977	92.0791	10.1923	10.1856	0.37133	87.9121	10.1924	9.55266	-9.55266	-0.99935	0.99935
67	8.36772	5.90906	84.0051	5.92467	0.70804	1.00264	0.70617	0.29383	155.337	45.185	-1.07127	-3.39182	107.528	3.55697	1.07126	3.39182	72.472	3.55697	-10.1802	-0.35715	92.0093	10.1865	10.1803	0.35872	87.9819	10.1866	9.59864	-9.59864	-1.00997	1.00997
68	8.31389	5.97815	83.9647	5.77775	0.69495	0.96648	0.71906	0.28094	156.142	45.2001	-1.07652	-3.24734	108.341	3.42114	1.07652	3.24735	71.6593	3.42114	-10.1758	-0.34268	91.9288	10.1816	10.1758	0.34429	88.0622	10.1817	9.64379	-9.64379	-1.01998	1.01998
69	8.261	6.04862	83.9255	5.62656	0.6811	0.93022	0.73219	0.26781	156.978	45.224	-1.08172	-3.10239	109.222	3.28556	1.08172	3.10238	70.7776	3.28556	-10.1739	-0.32951	91.855	10.1792	10.1739	0.32952	88.1449	10.1792	9.68931	-9.68931	-1.03031	1.03031
70	8.21844	6.09542	83.9018	5.51258	0.67076	0.90438	0.74168	0.25832	157.378	45.216	-1.08687	-2.95707	110.181	3.1505	1.08686	2.95708	69.8193	3.1505	-10.1743	-0.31604	91.7792	10.1792	10.1743	0.31603	88.2209	10.1792	9.73505	-9.73505	-1.0399	1.0399
71	8.18528	6.15631	83.8929	5.39431	0.65903	0.87623	0.75212	0.24788	158.308	45.2812	-1.09208	-2.81021	111.237	3.01492	1.09208	2.81018	68.7631	3.01492	-10.176	-0.30231	91.7017	10.1805	10.176	0.30066	88.3076	10.1805	9.78031	-9.78031	-1.04988	1.04988
72	8.13409	6.24367	83.8568	5.21344	0.64094	0.83499	0.76759	0.23241	159.551	45.3645	-1.09729	-2.64714	112.515	2.86551	1.09729	2.64709	67.4848	2.86551	-10.18	-0.28664	91.6129	10.184	10.18	0.28498	88.3965	10.184	9.82981	-9.82981	-1.05963	1.05963
73	8.08405	6.29515	83.8225	5.07178	0.62738	0.80567	0.77871	0.22129	159.877	45.3485	-1.10241	-2.4991	113.803	2.73147	1.1024	2.49913	66.197	2.73147	-10.1866	-0.27065	91.5219	10.1902	10.1866	0.27062	88.4782	10.1902	9.87523	-9.87523	-1.06868	1.06868
74	8.05256	6.36059	83.8175	4.93827	0.61325	0.77639	0.78989	0.21011	160.909	45.4364	-1.10771	-2.34887	115.248	2.59695	1.10771	2.34885	64.7515	2.59695	-10.194	-0.25608	91.439	10.1973	10.194	0.25609	88.5609	10.1972	9.91974	-9.91974	-1.07811	1.07811
75	8.01344	6.43998	83.8009	4.76885	0.59511	0.74051	0.80365	0.19635	162.126	45.5414	-1.11287	-2.18309	117.011	2.45038	1.11287	2.18309	62.9888	2.45038	-10.2047	-0.23779	91.3349	10.2074	10.2046	0.2378	88.6651	10.2074	9.96838	-9.96838	-1.08717	1.08717
76	7.95603	6.52071	83.7552	4.55837	0.57294	0.69906	0.81959	0.18041	162.983	45.5919	-1.11795	-2.03268	118.81	2.31985	1.11794	2.03271	61.1903	2.31985	-10.2181	-0.2226	91.248	10.2205	10.2183	0.22257	88.7522	10.2207	10.0139	-10.0139	-1.0959	1.0959
77	7.9172	6.57869	83.7398	4.40487	0.55637	0.66957	0.83094	0.16906	163.629	45.6373	-1.12322	-1.86397	121.073	2.17624	1.12323	1.86397	58.9268	2.17624	-10.2323	-0.20544	91.1502	10.2344	10.2323	0.20545	88.8497	10.2344	10.0611	-10.0611	-1.10428	1.10428
78	7.87044	6.65043	83.7115	4.20899	0.53478	0.63289	0.84499	0.15501	164.437	45.6992	-1.12837	-1.71114	123.402	2.0497	1.12837	1.71116	56.5985	2.0497	-10.2499	-0.18796	91.0506	10.2516	10.2498	0.18621	88.9592	10.2515	10.106	-10.106	-1.1126	1.1126
79	7.83478	6.7357	83.7016	4.00176	0.51077	0.59411	0.85972	0.14028	165.79	45.8397	-1.13349	-1.54166	126.325	1.91354	1.13348	1.5417	53.6761	1.91354	-10.2698	-0.17018	90.9494	10.2712	10.2699	0.17017	89.0507	10.2713	10.1535	-10.1535	-1.12064	1.12064
80	7.80996	6.8105	83.71	3.82263	0.48946	0.56128	0.87203	0.12797	167.101	45.9852	-1.13866	-1.37063	129.718	1.78191	1.13866	1.37065	50.282	1.78191	-10.2917	-0.15212	90.8468	10.2928	10.2917	0.15212	89.1532	10.2928	10.2001	-10.2001	-1.12845	1.12845
81	7.7554	6.89873	83.6696	3.54313	0.45686	0.51359	0.88954	0.11046	168.083	46.0767	-1.14376	-1.21523	133.265	1.66884	1.14376	1.21525	46.7358	1.66884	-10.3165	-0.13376	90.7429	10.3174	10.3166	0.13375	89.2572	10.3174	10.2447	-10.2447	-1.13591	1.13591
82	7.72238	6.97663	83.6653	3.31087	0.42874	0.47457	0.90343	0.09657	169.257	46.208	-1.14889	-1.04254	137.778	1.5514	1.14889	1.04253	42.2213	1.5514	-10.3435	-0.11692	90.6476	10.3442	10.3435	0.11333	89.3723	10.3441	10.2909	-10.2909	-1.14311	1.14311
83	7.6695	7.06775	83.6278	2.97793	0.38828	0.42134	0.92154	0.07846	170.294	46.3177	-1.15395	-0.8694	143.005	1.44482	1.15394	0.86945	36.9967	1.44482	-10.3735	-0.09801	90.5413	10.374	10.3736	0.09618	89.4688	10.374	10.3371	-10.3371	-1.14992	1.14992
84	7.6605	7.14872	83.6647	2.75301	0.35938	0.38511	0.93319	0.06681	172.042	46.5384	-1.15905	-0.69479	149.059	1.35135	1.15905	0.6948	30.941	1.35135	-10.4056	-0.07882	90.434	10.4059	10.4056	0.07882	89.566	10.4059	10.3824	-10.3824	-1.15636	1.15636
85	7.5978	7.2544	83.6111	2.25837	0.29724	0.31131	0.9548	0.0452	173.157	46.6658	-1.16413	-0.51931	155.959	1.2747	1.16414	0.51927	24.0396	1.2747	-10.4403	-0.06118	90.3358	10.4405	10.4402	0.05938	89.6741	10.4404	10.4273	-10.4273	-1.16262	1.16262
86	7.57027	7.33828	83.6173	1.85974	0.24566	0.25343	0.96936	0.03064	174.524	46.8394	-1.1691	-0.36008	162.881	1.22329	1.16909	0.36008	17.119	1.22329	-10.4782	-0.0378	90.2067	10.4783	10.4783	0.03779	89.7934	10.4784	10.472	-10.472	-1.16843	1.16843
87	7.57753	7.45833	83.6836	1.33876	0.17668	0.1795	0.98427	0.01573	177.549	47.2373	-1.17411	-0.18311	171.136	1.1883	1.17411	0.18311	8.86445	1.1883	-10.5184	-0.01964	90.107	10.5185	10.5184	0.01779	89.9031	10.5184	10.5168	-10.5168	-1.17394	1.17394
88	7.17739	9.97068	83.0345	0	0	0	1.38918	-0.38918	224.823	54.2302	-1.17906	-0.00553	179.731	1.17907	1.17906	0.00556	0.27019	1.17907	-10.5615	0.0006	269.997	10.5615	10.5615	-0.00061	-89.9967	10.5615	10.5615	-10.5615	-1.17906	1.17906

series short circuit	1. General ellipse parameter features										2. features extracted from locus																			
faulty disk	A`	B`	θ	f	e	e'	e/e'	g	$A_{ellipse}$	$c_{ellipse}$	I_{min}	ΔV_{Imin}	θ_{Imin}	Abs_{Imin}	I_{max}	ΔV_{Imax}	θ_{Imax}	Abs_{Imax}	ΔV_{min}	$I_{\Delta Vmin}$	$\theta_{\Delta Vmin}$	$Abs_{\Delta Vmin}$	Δv_{max}	$I_{\Delta Vmax}$	$\theta_{\Delta Vmax}$	$Abs_{\Delta Vmax}$	$\Delta V_{H(0)}$	$\Delta V_{L(0)}$	$I_{H(0)}$	$I_{L(0)}$
1	11.754	3.58761	86.3892	11.1931	0.95228	3.11994	0.30522	0.69478	132.477	51.6739	-0.77411	-10.1701	94.3527	10.1995	0.77411	10.1701	85.6473	10.1995	-12.2649	-0.64161	92.9946	12.2817	12.2649	0.64161	87.0054	12.2817	6.87146	-6.87146	-0.4331	0.4331
2	11.7024	3.5701	86.3477	11.1445	0.95233	3.12163	0.30507	0.69493	131.252	51.4435	-0.7796	-10.1255	94.4028	10.1555	0.7796	10.1255	85.5972	10.1555	-12.21	-0.64611	93.0291	12.2271	12.21	0.64611	86.9709	12.2271	6.8234	-6.8234	-0.43626	0.43626
3	11.6499	3.55232	86.3051	11.0951	0.95238	3.12335	0.30492	0.69508	130.013	51.2093	-0.78518	-10.0801	94.454	10.1107	0.78518	10.0801	85.546	10.1107	-12.1542	-0.65136	93.0676	12.1716	12.1542	0.65136	86.9324	12.1716	6.79078	-6.79078	-0.43846	0.43846
4	11.597	3.5344	86.2616	11.0453	0.95243	3.12508	0.30477	0.69523	128.769	50.9729	-0.79082	-10.0343	94.5063	10.0655	0.79082	10.0343	85.4937	10.0655	-12.0978	-0.65598	93.1037	12.1156	12.0978	0.65598	86.8963	12.1156	6.75786	-6.75786	-0.44171	0.44171
5	11.5371	3.53395	86.213	10.9825	0.95193	3.10772	0.30631	0.69369	128.087	50.7459	-0.79662	-9.98728	94.5604	10.019	0.79662	9.98728	85.4396	10.019	-12.0399	-0.66072	93.1411	12.058	12.0399	0.66072	86.8589	12.058	6.72403	-6.72403	-0.44501	0.44501
6	11.4825	3.5154	86.1673	10.9311	0.95198	3.1095	0.30615	0.69385	126.812	50.502	-0.80243	-9.94003	94.6153	9.97236	0.80243	9.94003	85.3847	9.97236	-11.9817	-0.66548	93.179	12.0002	11.9817	0.66548	86.821	12.0002	6.6901	-6.6901	-0.44836	0.44836
7	11.4273	3.49667	86.1206	10.8792	0.95203	3.1113	0.30599	0.69401	125.53	50.2555	-0.80832	-9.88178	94.6763	9.91478	0.80832	9.88177	85.3237	9.91478	-11.9229	-0.67029	93.2177	11.9418	11.9229	0.67029	86.7823	11.9418	6.67135	-6.67135	-0.45176	0.45176
8	11.3711	3.47761	86.0727	10.8263	0.95209	3.11313	0.30583	0.69417	124.232	50.0046	-0.8143	-9.83316	94.734	9.86682	0.8143	9.83316	85.266	9.86682	-11.8631	-0.67518	93.2575	11.8823	11.8631	0.67518	86.7425	11.8823	6.63637	-6.63637	-0.45521	0.45521
9	11.3141	3.45831	86.0236	10.7726	0.95214	3.11499	0.30566	0.69434	122.923	49.7501	-0.82037	-9.78384	94.793	9.81817	0.82037	9.78384	85.207	9.81817	-11.8023	-0.68015	93.2982	11.8219	11.8023	0.68015	86.7018	11.8219	6.6009	-6.6009	-0.45871	0.45871
10	11.2561	3.43869	85.9731	10.718	0.95219	3.11689	0.3055	0.6945	121.599	49.4912	-0.82655	-9.73366	94.8537	9.76869	0.82655	9.73366	85.1463	9.76869	-11.7406	-0.6852	93.3401	11.7606	11.7406	0.6852	86.6599	11.7606	6.56485	-6.56485	-0.46227	0.46227
11	11.1973	3.41882	85.9214	10.6626	0.95225	3.11881	0.30532	0.69468	120.265	49.2289	-0.83282	-9.68277	94.9159	9.71852	0.83282	9.68277	85.0841	9.71852	-11.678	-0.69032	93.383	11.6983	11.678	0.69032	86.617	11.6983	6.52831	-6.52831	-0.46588	0.46588
12	11.1375	3.39861	85.8682	10.6063	0.9523	3.12077	0.30515	0.69485	118.915	48.9619	-0.83919	-9.63095	94.9799	9.66744	0.83919	9.63095	85.0201	9.66744	-11.6142	-0.69553	93.4271	11.635	11.6142	0.69553	86.5729	11.635	6.49112	-6.49112	-0.46956	0.46956
13	11.0769	3.37818	85.8138	10.5492	0.95236	3.12275	0.30498	0.69502	117.558	48.6916	-0.84565	-9.57849	95.0453	9.61575	0.84565	9.57849	84.9547	9.61575	-11.5497	-0.70154	93.4759	11.571	11.5497	0.70154	86.5241	11.571	6.45349	-6.45349	-0.47218	0.47218
14	11.0153	3.35742	85.7578	10.4912	0.95242	3.12477	0.3048	0.6952	116.186	48.4168	-0.85221	-9.52511	95.1126	9.56315	0.85221	9.52511	84.8874	9.56315	-11.484	-0.70691	93.5224	11.5058	11.484	0.70691	86.4775	11.5058	6.41524	-6.41524	-0.47596	0.47596
15	10.9477	3.35342	85.6963	10.4214	0.95193	3.1077	0.30631	0.69369	115.335	48.1534	-0.85887	-9.471	95.1816	9.50986	0.85887	9.471	84.8184	9.50986	-11.4175	-0.71235	93.5701	11.4397	11.4175	0.71235	86.4299	11.4397	6.37649	-6.37649	-0.4798	0.4798
16	10.8842	3.33196	85.6372	10.3616	0.95199	3.10977	0.30613	0.69387	113.932	47.87	-0.86564	-9.41591	95.2526	9.45561	0.86564	9.41591	84.7473	9.45561	-11.3498	-0.71788	93.6192	11.3725	11.3498	0.71789	86.3808	11.3725	6.33706	-6.33706	-0.48371	0.48371
17	10.8198	3.31022	85.5765	10.301	0.95205	3.11186	0.30594	0.69406	112.519	47.5826	-0.87251	-9.35012	95.3311	9.39075	0.87251	9.35012	84.6689	9.39075	-11.2811	-0.7235	93.6695	11.3043	11.2811	0.7235	86.3305	11.3043	6.3118	-6.3118	-0.48767	0.48767
18	10.7543	3.28813	85.514	10.2392	0.95211	3.114	0.30575	0.69425	111.091	47.2903	-0.87949	-9.29332	95.4062	9.33485	0.87949	9.29332	84.5938	9.33485	-11.2113	-0.7292	93.7214	11.2349	11.2113	0.7292	86.2786	11.2349	6.27107	-6.27107	-0.4917	0.4917
19	10.6877	3.26572	85.4498	10.1765	0.95217	3.11617	0.30556	0.69444	109.651	46.9934	-0.88659	-9.2356	95.4834	9.27806	0.88659	9.2356	84.5166	9.27806	-11.1403	-0.735	93.7747	11.1645	11.1403	0.735	86.2253	11.1645	6.22972	-6.22972	-0.49581	0.49581
20	10.62	3.24295	85.3836	10.1128	0.95224	3.11838	0.30536	0.69464	108.197	46.6916	-0.89381	-9.17688	95.563	9.2203	0.89381	9.17688	84.437	9.2203	-11.0681	-0.74089	93.8296	11.0929	11.0681	0.74089	86.1704	11.0929	6.18768	-6.18768	-0.49998	0.49998
21	10.5513	3.21986	85.3155	10.048	0.9523	3.12063	0.30516	0.69484	106.731	46.385	-0.90115	-9.11721	95.6448	9.16164	0.90115	9.11721	84.3552	9.16164	-10.9948	-0.74688	93.8862	11.0201	10.9948	0.74688	86.1138	11.0201	6.145	-6.145	-0.50422	0.50422
22	10.4815	3.19645	85.2455	9.98222	0.95236	3.1229	0.30496	0.69504	105.255	46.074	-0.90859	-9.05665	95.7289	9.10211	0.90859	9.05665	84.2711	9.10211	-10.9203	-0.75375	93.9485	10.9463	10.9203	0.75375	86.0515	10.9463	6.10171	-6.10171	-0.50734	0.50734
23	10.4104	3.17262	85.1731	9.91516	0.95243	3.12523	0.30476	0.69524	103.761	45.757	-0.91618	-8.99487	95.8158	9.04141	0.91618	8.99487	84.1841	9.04141	-10.8444	-0.75995	94.0086	10.871	10.8444	0.75995	85.9914	10.871	6.05759	-6.05759	-0.51172	0.51172
24	10.3333	3.16447	85.094	9.83687	0.95195	3.10853	0.30624	0.69376	102.729	45.4497	-0.92388	-8.93218	95.9053	8.97983	0.92388	8.93218	84.0947	8.97983	-10.7675	-0.76624	94.0704	10.7947	10.7675	0.76624	85.9296	10.7947	6.01285	-6.01285	-0.51617	0.51617
25	10.26	3.13984	85.0172	9.76777	0.95202	3.11092	0.30603	0.69397	101.206	45.1227	-0.93171	-8.85903	96.0038	8.90789	0.93171	8.85903	83.9962	8.90789	-10.6892	-0.77263	94.1342	10.7171	10.6892	0.77263	85.8658	10.7171	5.98131	-5.98131	-0.5207	0.5207
26	10.1853	3.11475	84.9377	9.69732	0.95209	3.11335	0.30581	0.69419	99.6658	44.7895	-0.93969	-8.79406	96.0992	8.84413	0.93969	8.79406	83.9008	8.84413	-10.6094	-0.77914	94.2002	10.6379	10.6094	0.77914	85.7998	10.6379	5.93488	-5.93488	-0.52532	0.52532
27	10.1095	3.08935	84.8559	9.62585	0.95216	3.11582	0.30559	0.69441	98.1172	44.4516	-0.94779	-8.72815	96.1975	8.77946	0.94779	8.72815	83.8025	8.77946	-10.5284	-0.78575	94.2681	10.5577	10.5284	0.78575	85.7319	10.5577	5.8878	-5.8878	-0.53	0.53
28	10.0322	3.06349	84.7712	9.55298	0.95223	3.11833	0.30537	0.69463	96.5519	44.1071	-0.95604	-8.66093	96.2991	8.71353	0.95604	8.66093	83.7009	8.71353	-10.4458	-0.79248	94.3385	10.4758	10.4458	0.79248	85.6615	10.4758	5.83983	-5.83983	-0.53478	0.53478
29	9.95365	3.03726	84.6838	9.47894	0.95231	3.12089	0.30514	0.69486	94.976	43.7574	-0.96443	-8.59261	96.4041	8.64656	0.96443	8.59261	83.5959	8.64656	-10.3619	-0.79932	94.4111	10.3927	10.3619	0.80017	85.5843	10.3928	5.79113	-5.79113	-0.53965	0.53965
30	9.87374	3.01059	84.5934	9.40356	0.95238	3.12349	0.30491	0.69509	93.3864	43.4014	-0.97297	-8.52303	96.5126	8.57839	0.97297	8.52303	83.4874	8.57839	-10.2766	-0.80714	94.4909	10.3082	10.2766	0.80714	85.5091	10.3082	5.74157	-5.74157	-0.54333	0.54333
31	9.78781	2.9986	84.4947	9.31717	0.95192	3.10717	0.30636	0.69364	92.205	43.0527	-0.98167	-8.45221	96.6248	8.50902	0.98167	8.45221	83.3752	8.50902	-10.1897	-0.81423	94.5686	10.2221	10.1897	0.81423	85.4314	10.2221	5.69117	-5.69117	-0.54836	0.54836
32	9.70507	2.97093	84.3978	9.23915	0.95199	3.10986	0.30612	0.69388	90.5816	42.684	-0.99051	-8.38006	96.741	8.43839	0.99051	8.38006	83.259	8.43839	-10.1012	-0.82144	94.6491	10.1345	10.1012	0.82144	85.3509	10.1345	5.63988	-5.63988	-0.55349	0.55349
33	9.62078	2.94278	84.2973	9.15967	0.95207	3.11259	0.30588	0.69412	88.9442	42.3085	-0.99953	-8.29775	96.8686	8.35773	0.99953	8.29775	83.1314	8.35773	-10.011	-0.82879	94.7326	10.0453	10.011	0.82879	85.2674	10.0453	5.60069	-5.60069	-0.55872	0.55872
34	9.53503	2.91419	84.1933	9.07879	0.95215	3.11537	0.30563	0.69437	87.295	41.9266	-1.0087	-8.22298	96.9935	8.28462	1.0087	8.22298	83.0065	8.28462	-9.91927	-0.83627	94.8191	9.95446	9.91927	0.83627	85.1809	9.95446	5.54747	-5.54747	-0.56404	0.56404
35	9.44767	2.8851	84.0853	8.99637	0.95223	3.11821	0.30538	0.69462	85.632	41.5376	-1.01805	-8.14677	97.123	8.21014	1.01805	8.14677	82.877	8.21014	-9.82577	-0.84388	94.9088	9.86194	9.82577	0.84388	85.0912	9.86194	5.49326	-5.49326	-0.56946	0.56946
36	9.35869	2.85553	83.9732	8.9124	0.95231	3.1211	0.30512	0.69488	83.956	41.1415	-1.02758	-8.06911	97.2574	8.13427	1.02758	8.06911	82.7426	8.13427	-9.73052	-0.85164	95.0019	9.76772	9.73052	0.85164	84.9981	9.76772	5.43808	-5.43808	-0.57499	0.57499
37	9.26811	2.82548	83.8569	8.82692	0.9524	3.12404	0.30486	0.69514	82.2685	40.7384	-1.03728	-7.99	97.3969	8.05705	1.03728	7.99	82.6031	8.05705	-9.63355	-0.86045	95.104	9.6719	9.63355	0.86045	84.896	9.6719	5.38193	-5.38193	-0.57928	0.57928
38	9.17141	2.80894	83.7299	8.73067	0.95194	3.10817	0.30627	0.69373	80.9336	40.3397	-1.04717	-7.90925	97.542	7.97827	1.04717	7.90925	82.458	7.97827	-9.53459	-0.86851	95.2047	9.57406	9.53459	0.86851	84.7953	9.57406	5.32467	-5.32467	-0.58502	0.58502
39	9.07741	2.77769	83.604	8.64198	0.95203	3.11121	0.306	0.694	79.2128	39.9212	-1.05724	-7.81872	97.7008	7.88988	1.05724	7.81872	82.2992	7.88988	-9.43383	-0.87672	95.3094	9.47448	9.43383	0.87672	84.6906	9.47448	5.2787	-5.2787	-0.59087	0.59087
40	8.98167	2.74592	83.473	8.55163	0.95212	3.11431	0.30572	0.69428	77.4809	39.4951	-1.06751	-7.735	97.8578	7.80831	1.06751	7.735	82.1422	7.80831	-9.33118	-0.88508	95.4184	9.37306	9.33118	0.88508	84.5816	9.37306	5.21926	-5.21926	-0.59684	0.59684
41	8.88396	2.71355	83.3364	8.4594	0.95221	3.11747	0.30544	0.69456	75.7345	39.0604	-1.07799	-7.64948	98.0215	7.72507	1.07799	7.64948	81.9785	7.72507	-9.22637	-0.8936	95.532	9.26955	9.22637	0.8936	84.468	9.26955	5.15862	-5.15862	-0.60293	0.60293
42	8.78443	2.68064	83.194	8.36543	0.9523	3.12068	0.30516	0.69484	73.9779	38.6177	-1.08867	-7.56232	98.192	7.64028	1.08867	7.56232	81.808	7.64028	-9.11959	-0.90229	95.6505	9.16411	9.11959	0.90229	84.3495	9.16411	5.09689	-5.09689	-0.60915	0.60915
43	8.68288	2.64713	83.0454	8.26953	0.95239	3.12397	0.30487	0.69513	72.2085	38.1661	-1.09957	-7.47331	98.37	7.55377	1.09957	7.47331	81.63	7.55377	-9.01059	-0.91212	95.7802	9.05663	9.01059	0.91212	84.2198	9.05663	5.03391	-5.03391	-0.61406	0.61406
44	8.5754	2.6261	82.8834	8.1634	0.95196	3.10856	0.30624	0.69376	70.7483	37.7176	-1.11068	-7.38253	98.5558	7.46561	1.11068	7.38253	81.4442	7.46561	-8.89946	-0.92117	95.9095	8.947	8.89946	0.92117	84.0905	8.947	4.96976	-4.96976	-0.62053	0.62053
45	8.46982	2.59119	82.7208	8.06372	0.95205	3.11197	0.30593	0.69407	68.9483	37.2479	-1.12202	-7.28207	98.7593	7.36801	1.12202	7.28207	81.2407	7.36801	-8.78596	-0.9304	96.0449	8.83509	8.78596	0.9304	83.9551	8.83509	4.91574	-4.91574	-0.62713	0.62713
46	8.36221	2.55569	82.5509	7.96209	0.95215	3.11543	0.30562	0.69438	67.1397	36.7694	-1.1336	-7.18757	98.9626	7.27641	1.1336	7.18757	81.0374	7.27641	-8.67024	-0.93981	96.1864	8.72102	8.67024	0.93981	83.8136	8.72102	4.8489	-4.8489	-0.63388	0.63388
47	8.25231	2.51951	82.3726	7.85829	0.95225	3.11897	0.30531	0.69469	65.3194	36.2809	-1.14541	-7.09097	99.1758	7.18289	1.14541	7.09097	80.8242	7.18289	-8.552	-0.94941	96.3349	8.60453	8.552	0.94941	83.6652	8.60453	4.78066	-4.78066	-0.64077	0.64077
48	8.14022	2.48269	82.1857	7.75238	0.95236	3.12257	0.30499	0.69501	63.4905	35.7828	-1.15748	-6.99235	99.3992	7.0875	1.15748	6.99235	80.6008	7.0875	-8.43132	-0.96024	96.4974	8.48582	8.43132	0.96024	83.5026	8.48582	4.71108	-4.71108	-0.6463	0.6463
49	8.02208	2.45729	81.9818	7.63646	0.95193	3.10767	0.30632	0.69368	61.929	35.2852	-1.1698	-6.89155	99.6338	6.99013	1.1698	6.89155	80.3662	6.99013	-8.30804	-0.97026	96.6611	8.36451	8.30804	0.97026	83.3389	8.36451	4.64006	-4.64006	-0.65348	0.65348
50	7.90533	2.41889	81.7752	7.52617	0.95204	3.11142	0.30598	0.69402	60.0739	34.7663	-1.18239	-6.78137	99.8906	6.88368	1.18239	6.78137	80.1094	6.88368	-8.18213	-0.98049	96.8333	8.24067	8.18213	0.98049	83.1667	8.24067	4.57824	-4.57824	-0.66083	0.66083
51	7.78607	2.37975	81.5576	7.41348	0.95215	3.11524	0.30564	0.69436	58.2103	34.2364	-1.19526	-6.67615	100.15	6.7823	1.19526	6.67615	79.8496	6.7823	-8.05342	-0.99094	97.0148	8.11415	8.05342	0.99095	82.9852	8.11415	4.50405	-4.50405	-0.66834	0.66834
52	7.66439	2.33991	81.3284	7.29847	0.95226	3.11913	0.3053	0.6947	56.3412	33.6959	-1.2084	-6.56866	100.424	6.67889	1.2084	6.56866	79.5761	6.67889	-7.92198	-1.00162	97.206	7.98505	7.92198	1.00162	82.794	7.98505	4.42837	-4.42837	-0.67602	0.67602
53	7.54008	2.29931	81.0865	7.18094	0.95237	3.12309	0.30494	0.69506	54.4656	33.144	-1.22184	-6.45871	100.712	6.57326	1.22184	6.45871	79.2876	6.57326	-7.7876	-1.01359	97.4156	7.85328	7.7876	1.01359	82.5844	7.85328	4.35107	-4.35107	-0.68229	0.68229
54	7.4097	2.26898	80.822	7.05375	0.95196	3.10877	0.30622	0.69378	52.818	32.5902	-1.23557	-6.34622	101.017	6.46538	1.23557	6.34622	78.9827	6.46538	-7.65019	-1.02475	97.6294	7.71851	7.65019	1.02475	82.3706	7.71851	4.2721	-4.2721	-0.69032	0.69032
55	7.28004	2.2266	80.5509	6.93118	0.95208	3.1129	0.30585	0.69415	50.9244	32.0144	-1.24963	-6.22455	101.352	6.34875	1.24963	6.22455	78.6483	6.34875	-7.50967	-1.03616	97.8559	7.58082	7.50967	1.03616	82.1441	7.58082	4.20121	-4.20121	-0.69853	0.69853
56	7.1474	2.18335	80.2631	6.80576	0.9522	3.11712	0.30547	0.69453	49.0254	31.4256	-1.264	-6.10677	101.694	6.23621	1.264	6.10677	78.3058	6.23621	-7.36577	-1.04782	98.0963	7.43993	7.36577	1.04782	81.9037	7.43993	4.11849	-4.11849	-0.70695	0.70695
57	7.01197	2.13932	79.9576	6.67765	0.95232	3.1214	0.30509	0.69491	47.1264	30.8247	-1.27871	-5.98631	102.058	6.12135	1.27871	5.98631	77.9424	6.12135	-7.21867	-1.06087	98.3605	7.29621	7.21867	1.06087	81.6395	7.29621	4.03403	-4.03403	-0.71557	0.71557
58	6.87038	2.1045	79.6226	6.54012	0.95193	3.10768	0.30632	0.69368	45.4235	30.2194	-1.29377	-5.86289	102.444	6.00394	1.29377	5.86289	77.5559	6.00394	-7.06804	-1.07309	98.6329	7.14904	7.06804	1.07309	81.3671	7.14904	3.94763	-3.94763	-0.72271	0.72271
59	6.7289	2.05848	79.2754	6.4063	0.95206	3.11215	0.30592	0.69408	43.5152	29.5916	-1.30918	-5.73043	102.869	5.87807	1.30918	5.73043	77.131	5.87807	-6.91386	-1.08559	98.9235	6.99857	6.91386	1.08559	81.0765	6.99857	3.86829	-3.86829	-0.73175	0.73175
60	6.58419	2.01155	78.9044	6.26939	0.95219	3.1167	0.30551	0.69449	41.6086	28.9498	-1.32497	-5.60102	103.309	5.7556	1.32497	5.60102	76.6908	5.7556	-6.75591	-1.09839	99.2344	6.84462	6.75591	1.09839	80.7656	6.84462	3.77769	-3.77769	-0.74101	0.74101
61	6.43624	1.96371	78.507	6.12935	0.95232	3.12131	0.3051	0.6949	39.7063	28.2939	-1.34114	-5.4684	103.78	5.63046	1.34114	5.4684	76.2201	5.63046	-6.59414	-1.11149	99.5676	6.68715	6.59414	1.11148	80.4324	6.68715	3.685	-3.685	-0.74876	0.74876
62	6.2822	1.92398	78.0693	5.98033	0.95195	3.10831	0.30626	0.69374	37.972	27.6316	-1.35771	-5.33246	104.285	5.50259	1.35771	5.33246	75.7153	5.50259	-6.42841	-1.12609	99.9359	6.52629	6.42841	1.12609	80.0641	6.52629	3.59017	-3.59017	-0.75848	0.75848
63	6.12753	1.87398	77.6096	5.83394	0.95209	3.11313	0.30583	0.69417	36.0744	26.9459	-1.37469	-5.18751	104.842	5.36657	1.37469	5.18751	75.1577	5.36657	-6.25848	-1.13985	100.322	6.36144	6.25848	1.13985	79.6779	6.36144	3.5012	-3.5012	-0.76845	0.76845
64	5.9693	1.82299	77.1136	5.68412	0.95223	3.11801	0.30539	0.69461	34.1868	26.2448	-1.39211	-5.04457	105.427	5.23313	1.39211	5.04457	74.5725	5.23313	-6.08423	-1.15395	100.739	6.19269	6.08423	1.15395	79.2607	6.19269	3.4015	-3.4015	-0.77869	0.77869
65	5.80747	1.77103	76.5768	5.53084	0.95237	3.12294	0.30496	0.69504	32.312	25.5281	-1.40997	-4.89789	106.06	5.09679	1.40997	4.89789	73.9403	5.09679	-5.90553	-1.16964	101.203	6.02024	5.90553	1.16964	78.7971	6.02024	3.29939	-3.29939	-0.78737	0.78737
66	5.63952	1.72589	75.9809	5.36894	0.95202	3.11083	0.30603	0.69397	30.5777	24.8022	-1.4283	-4.74726	106.745	4.95747	1.4283	4.74726	73.2551	4.95747	-5.72214	-1.18448	101.695	5.84344	5.72214	1.18448	78.305	5.84344	3.19474	-3.19474	-0.79816	0.79816
67	5.47036	1.67162	75.3456	5.2087	0.95217	3.11597	0.30558	0.69442	28.7278	24.0532	-1.4471	-4.58778	107.507	4.81059	1.4471	4.58778	72.4934	4.81059	-5.53399	-1.19968	102.232	5.66253	5.53399	1.19968	77.7684	5.66253	3.09473	-3.09473	-0.80923	0.80923
68	5.29734	1.61632	74.651	5.04473	0.95231	3.12112	0.30512	0.69488	26.8989	23.2875	-1.46641	-4.42905	108.319	4.6655	1.46641	4.42905	71.6809	4.6655	-5.34078	-1.21658	102.833	5.47759	5.34078	1.21658	77.1675	5.47759	2.98452	-2.98452	-0.81871	0.81871
69	5.11839	1.56683	73.8742	4.87268	0.95199	3.10989	0.30612	0.69388	25.1945	22.5112	-1.48624	-4.26595	109.208	4.51744	1.48624	4.26595	70.7919	4.51744	-5.14237	-1.23262	103.479	5.28804	5.14237	1.23262	76.5206	5.28804	2.87152	-2.87152	-0.8304	0.8304
70	4.93772	1.50917	73.0336	4.70143	0.95215	3.11524	0.30564	0.69436	23.4107	21.7118	-1.50661	-4.09931	110.204	4.36234	1.50661	4.09931	69.7957	4.36234	-4.9385	-1.24908	104.194	5.09402	4.9385	1.24908	75.806	5.09402	2.76201	-2.76201	-0.84243	0.84243
71	4.75319	1.45054	72.1032	4.52645	0.9523	3.12052	0.30517	0.69483	21.6603	20.8959	-1.52755	-3.92163	111.282	4.20864	1.52755	3.92163	68.7181	4.20864	-4.729	-1.26599	104.987	4.89552	4.729	1.26599	75.0129	4.89552	2.64277	-2.64277	-0.8548	0.8548
72	4.56302	1.3966	71.0511	4.34403	0.95201	3.11043	0.30607	0.69393	20.0205	20.0682	-1.54908	-3.74432	112.476	4.05211	1.54908	3.74432	67.5243	4.05211	-4.51353	-1.28473	105.888	4.69281	4.51353	1.28473	74.1116	4.69281	2.52033	-2.52033	-0.86553	0.86553
73	4.37097	1.33572	69.8927	4.16188	0.95216	3.11584	0.30559	0.69441	18.3418	19.2193	-1.57123	-3.55806	113.826	3.88955	1.57123	3.55806	66.1739	3.88955	-4.29194	-1.3026	106.883	4.48526	4.29194	1.3026	73.117	4.48526	2.4002	-2.4002	-0.87863	0.87863
74	4.17528	1.27399	68.5895	3.97617	0.95231	3.12103	0.30513	0.69487	16.711	18.3549	-1.59401	-3.3703	115.312	3.72825	1.59401	3.3703	64.6878	3.72825	-4.06388	-1.32238	108.025	4.27362	4.06388	1.32238	71.9752	4.27362	2.27072	-2.27072	-0.89005	0.89005
75	3.97489	1.2159	67.0946	3.78435	0.95207	3.11239	0.30589	0.69411	15.1835	17.4802	-1.61747	-3.17349	117.007	3.56191	1.61747	3.17349	62.9928	3.56191	-3.82909	-1.34131	109.305	4.05722	3.82909	1.34131	70.6949	4.05722	2.14264	-2.14264	-0.90394	0.90394
76	3.77307	1.15245	65.4129	3.59276	0.95221	3.11751	0.30544	0.69456	13.6604	16.5891	-1.64163	-2.97437	118.895	3.39733	1.64163	2.97437	61.1046	3.39733	-3.58737	-1.36078	110.773	3.83679	3.58737	1.36078	69.227	3.83679	2.00557	-2.00557	-0.91827	0.91827
77	3.56775	1.09199	63.4602	3.39653	0.95201	3.11042	0.30607	0.69393	12.2394	15.691	-1.66652	-2.76901	121.042	3.23182	1.66652	2.76901	58.9585	3.23182	-3.33826	-1.38229	112.493	3.61313	3.33826	1.38229	67.5068	3.61313	1.86457	-1.86457	-0.93089	0.93089
78	3.36202	1.02756	61.2254	3.20114	0.95215	3.11529	0.30564	0.69436	10.8532	14.7832	-1.69217	-2.55443	123.522	3.06408	1.69217	2.55443	56.4777	3.06408	-3.0815	-1.40296	114.479	3.38584	3.0815	1.40296	65.521	3.38584	1.72352	-1.72352	-0.94613	0.94613
79	3.15585	0.96346	58.6191	3.00518	0.95226	3.11914	0.30529	0.69471	9.55214	13.8744	-1.71863	-2.336	126.342	2.9001	1.71863	2.336	53.6575	2.9001	-2.81672	-1.42576	116.847	3.15701	2.81672	1.42576	63.1526	3.15701	1.57387	-1.57387	-0.95963	0.95963
80	2.95033	0.90192	55.5285	2.80909	0.95213	3.11457	0.3057	0.6943	8.35963	12.9733	-1.74592	-2.10819	129.63	2.73728	1.74592	2.10819	50.3698	2.73728	-2.54355	-1.44773	119.648	2.9267	2.54355	1.44773	60.3525	2.9267	1.42309	-1.42309	-0.97587	0.97587
81	2.74893	0.83972	51.8929	2.61753	0.9522	3.11715	0.30547	0.69453	7.25184	12.0865	-1.7741	-1.8753	133.411	2.58151	1.7741	1.8753	46.5885	2.58151	-2.26149	-1.47039	123.031	2.69747	2.26149	1.47039	56.9686	2.69747	1.26395	-1.26395	-0.99266	0.99266
82	2.5542	0.7806	47.5266	2.432	0.95216	3.11553	0.30562	0.69438	6.26376	11.231	-1.80319	-1.63285	137.838	2.43263	1.80319	1.63285	42.1619	2.43263	-1.97022	-1.49535	127.198	2.47343	1.97022	1.49535	52.8025	2.47343	1.10253	-1.10253	-1.00769	1.00769
83	2.37109	0.72461	42.3236	2.25766	0.95216	3.11569	0.3056	0.6944	5.39761	10.4258	-1.83326	-1.3841	142.948	2.29708	1.83326	1.3841	37.0525	2.29708	-1.66922	-1.52112	132.342	2.25833	1.66922	1.51951	47.688	2.25725	0.93304	-0.93304	-1.02565	1.02565
84	2.20594	0.67371	36.0622	2.10054	0.95222	3.11787	0.30541	0.69459	4.66892	9.69875	-1.86435	-1.12666	148.855	2.17834	1.86435	1.12666	31.1453	2.17834	-1.35803	-1.5461	138.705	2.05783	1.35803	1.5461	41.2948	2.05783	0.75821	-0.75821	-1.04182	1.04182
85	2.06663	0.6319	28.6534	1.96765	0.95211	3.11389	0.30576	0.69424	4.10259	9.08775	-1.89651	-0.85914	155.629	2.08203	1.89651	0.85914	24.3709	2.08203	-1.0361	-1.57191	146.61	1.88265	1.0361	1.57191	33.3903	1.88265	0.57913	-0.57913	-1.06107	1.06107
86	1.96434	0.59957	20.0078	1.8706	0.95228	3.11988	0.30523	0.69477	3.70006	8.63581	-1.92979	-0.58253	163.203	2.0158	1.92979	0.58253	16.797	2.0158	-0.70286	-1.60028	156.288	1.74783	0.70286	1.60028	23.7116	1.74783	0.39329	-0.39329	-1.07852	1.07852
87	1.90916	0.58428	10.3221	1.81755	0.95202	3.11075	0.30604	0.69396	3.5044	8.39638	-1.96427	-0.29659	171.414	1.98653	1.96427	0.29659	8.58644	1.98653	-0.35765	-1.62792	167.609	1.66674	0.35765	1.62792	12.3909	1.66674	0.19986	-0.19986	-1.09664	1.09664
88	1.99999	0.00106	5.7E-07	1.99999	1	1885.5	0.00053	0.99947	0.00666	7.96711	-1.99999	-2E-08	180	1.99999	1.99999	2E-08	5.7E-07	1.99999	-2E-08	-1.99999	180	1.99999	2E-08	1.99999	5.7E-07	1.99999	1.1E-11	-1.1E-11	-0.00106	0.00106

shunt short circuit	***1. General ellipse parameter features***										***2. features extracted from locus***																			
faulty disk	A'	B'	θ	f	e	e'	$\frac{e}{e'}$	g	$A_{ellipse}$	$c_{ellipse}$	I_{min}	ΔV_{Imin}	θ_{Imin}	Abs_{Imin}	I_{max}	ΔV_{Imax}	θ_{Imax}	Abs_{Imax}	ΔV_{min}	$I_{\Delta Vmin}$	$\theta_{\Delta Vmin}$	$Abs_{\Delta Vmin}$	Δv_{max}	$I_{\Delta Vmax}$	$\theta_{\Delta Vmax}$	$Abs_{\Delta Vmax}$	$\Delta V_{H(0)}$	$\Delta V_{L(0)}$	$I_{H(0)}$	$I_{L(0)}$
1	19.1623	5.8621	86.4286	18.2436	0.95206	3.11212	0.30592	0.69408	352.899	84.27	-1.24802	-16.5768	94.3055	16.6238	1.24802	16.5768	85.6945	16.6238	-20	-1.03486	92.962	20.0268	20	1.03486	87.038	20.0268	11.1896	-11.1896	-0.69759	0.69759
2	19.1632	5.86237	86.3875	18.2444	0.95206	3.11213	0.30592	0.69408	352.931	84.2738	-1.26242	-16.5768	94.355	16.6248	1.26242	16.5768	85.645	16.6248	-20	-1.0468	92.9961	20.0274	20	1.0468	87.0039	20.0274	11.1896	-11.1896	-0.70563	0.70563
3	19.164	5.86264	86.3457	18.2453	0.95206	3.11213	0.30592	0.69408	352.964	84.2777	-1.27706	-16.5768	94.4053	16.626	1.27706	16.5768	85.5947	16.626	-20	-1.05894	93.0308	20.028	20	1.05894	86.9692	20.028	11.1896	-11.1896	-0.71382	0.71382
4	19.165	5.86292	86.3028	18.2461	0.95206	3.11213	0.30592	0.69408	352.998	84.2817	-1.29212	-16.5768	94.457	16.6271	1.29212	16.5768	85.543	16.6271	-20	-1.07143	93.0665	20.0287	20	1.07143	86.9335	20.0287	11.1896	-11.1896	-0.72223	0.72223
5	19.1659	5.86321	86.259	18.2471	0.95206	3.11213	0.30592	0.69408	353.033	84.2859	-1.30747	-16.5768	94.5098	16.6283	1.30747	16.5768	85.4902	16.6283	-20	-1.08416	93.1028	20.0294	20	1.08416	86.8972	20.0294	11.1896	-11.1896	-0.73081	0.73081
6	19.1669	5.86351	86.2139	18.248	0.95206	3.11213	0.30592	0.69408	353.069	84.2903	-1.32326	-16.5768	94.564	16.6296	1.32326	16.5768	85.436	16.6296	-20	-1.09725	93.1402	20.0301	20	1.09725	86.8598	20.0301	11.1896	-11.1896	-0.73964	0.73964
7	19.1679	5.86382	86.168	18.249	0.95206	3.11213	0.30592	0.69408	353.107	84.2948	-1.33937	-16.5768	94.6193	16.6309	1.33937	16.5768	85.3807	16.6309	-20	-1.11061	93.1784	20.0308	20	1.11061	86.8216	20.0308	11.1896	-11.1896	-0.74865	0.74865
8	19.169	5.86415	86.1208	18.25	0.95206	3.11213	0.30592	0.69408	353.146	84.2994	-1.35589	-16.5768	94.6761	16.6322	1.35589	16.5768	85.3239	16.6322	-20	-1.12431	93.2175	20.0316	20	1.1243	86.7825	20.0316	11.1896	-11.1896	-0.75788	0.75788
9	19.1701	5.86448	86.0724	18.251	0.95206	3.11213	0.30592	0.69408	353.186	84.3043	-1.37287	-16.5768	94.7343	16.6336	1.37287	16.5768	85.2657	16.6336	-20	-1.13838	93.2577	20.0324	20	1.13838	86.7423	20.0324	11.1896	-11.1896	-0.76737	0.76737
10	19.1712	5.86483	86.0229	18.2521	0.95206	3.11213	0.30592	0.69408	353.228	84.3093	-1.39023	-16.5768	94.7939	16.635	1.39023	16.5768	85.2061	16.635	-20	-1.15278	93.2988	20.0332	20	1.15278	86.7012	20.0332	11.1896	-11.1896	-0.77707	0.77707
11	19.1724	5.86519	85.9721	18.2533	0.95206	3.11213	0.30592	0.69408	353.272	84.3146	-1.40806	-16.5768	94.8551	16.6365	1.40806	16.5768	85.1449	16.6365	-20	-1.16756	93.341	20.0341	20	1.16756	86.659	20.0341	11.1896	-11.1896	-0.78704	0.78704
12	19.1737	5.86557	85.9198	18.2544	0.95206	3.11213	0.30592	0.69408	353.318	84.32	-1.42638	-16.5768	94.918	16.6381	1.42639	16.5768	85.082	16.6381	-20	-1.18276	93.3844	20.0349	20	1.18276	86.6156	20.0349	11.1896	-11.1896	-0.79728	0.79728
13	19.1749	5.86596	85.8666	18.2557	0.95206	3.11214	0.30592	0.69408	353.365	84.3256	-1.44505	-16.5768	94.982	16.6397	1.44504	16.5768	85.018	16.6397	-20	-1.19823	93.4286	20.0359	20	1.19823	86.5714	20.0359	11.1896	-11.1896	-0.80771	0.80771
14	19.1763	5.86637	85.8115	18.2569	0.95206	3.11214	0.30592	0.69408	353.414	84.3315	-1.46439	-16.5768	95.0484	16.6414	1.46439	16.5768	84.9516	16.6414	-20	-1.21427	93.4744	20.0368	20	1.21427	86.5256	20.0368	11.1896	-11.1896	-0.81852	0.81852
15	19.1777	5.86679	85.755	18.2583	0.95206	3.11214	0.30592	0.69408	353.465	84.3377	-1.4842	-16.5768	95.1163	16.6432	1.4842	16.5768	84.8837	16.6432	-20	-1.2307	93.5213	20.0378	20	1.2307	86.4787	20.0378	11.1896	-11.1896	-0.8296	0.8296
16	19.1791	5.86723	85.6973	18.2596	0.95206	3.11214	0.30592	0.69408	353.518	84.344	-1.50444	-16.5768	95.1857	16.645	1.50444	16.5768	84.8143	16.645	-20	-1.24749	93.5692	20.0389	20	1.24748	86.4308	20.0389	11.1896	-11.1896	-0.84091	0.84091
17	19.1806	5.86769	85.6378	18.2611	0.95206	3.11214	0.30592	0.69408	353.574	84.3506	-1.52535	-16.5768	95.2574	16.6469	1.52535	16.5768	84.7426	16.6469	-20	-1.26482	93.6186	20.04	20	1.26481	86.3814	20.04	11.1896	-11.1896	-0.8526	0.8526
18	19.1822	5.86817	85.5762	18.2626	0.95206	3.11214	0.30592	0.69408	353.632	84.3576	-1.54696	-16.5768	95.3314	16.6489	1.54696	16.5768	84.6686	16.6489	-20	-1.28274	93.6697	20.0411	20	1.28274	86.3302	20.0411	11.1896	-11.1896	-0.86463	0.86463
19	19.1839	5.86867	85.5136	18.2641	0.95206	3.11214	0.30592	0.69408	353.692	84.3648	-1.56895	-16.5768	95.4068	16.6509	1.56895	16.5768	84.5932	16.6509	-20	-1.30097	93.7218	20.0423	20	1.30097	86.2782	20.0423	11.1896	-11.1896	-0.87697	0.87697
20	19.1856	5.86919	85.449	18.2658	0.95206	3.11214	0.30592	0.69408	353.755	84.3723	-1.59164	-16.5768	95.4845	16.6531	1.59164	16.5768	84.5155	16.6531	-20	-1.31979	93.7754	20.0435	20	1.31978	86.2246	20.0435	11.1896	-11.1896	-0.88965	0.88965
21	19.1874	5.86974	85.3824	18.2675	0.95206	3.11215	0.30592	0.69408	353.821	84.3801	-1.61502	-16.5768	95.5646	16.6553	1.61502	16.5768	84.4354	16.6553	-20	-1.33918	93.8307	20.0448	20	1.33917	86.1693	20.0448	11.1896	-11.1896	-0.90272	0.90272
22	19.1892	5.87031	85.3134	18.2693	0.95206	3.11215	0.30592	0.69408	353.89	84.3884	-1.63927	-16.5768	95.6476	16.6577	1.63928	16.5768	84.3524	16.6577	-20	-1.35929	93.8881	20.0461	20	1.35929	86.1119	20.0461	11.1896	-11.1896	-0.91628	0.91628
23	19.1912	5.8709	85.2428	18.2711	0.95206	3.11215	0.30592	0.69408	353.962	84.397	-1.66407	-16.5768	95.7325	16.6602	1.66407	16.5768	84.2675	16.6602	-20	-1.37985	93.9467	20.0475	20	1.37985	86.0533	20.0475	11.1896	-11.1896	-0.93014	0.93014
24	19.1932	5.87153	85.1699	18.2731	0.95206	3.11215	0.30592	0.69408	354.037	84.406	-1.6897	-16.5768	95.8201	16.6627	1.6897	16.5768	84.1799	16.6627	-20	-1.40109	94.0073	20.049	20	1.4011	85.9927	20.049	11.1896	-11.1896	-0.94446	0.94446
25	19.1954	5.87218	85.0948	18.2751	0.95206	3.11215	0.30592	0.69408	354.116	84.4154	-1.71608	-16.5768	95.9104	16.6654	1.71608	16.5768	84.0896	16.6654	-20	-1.42297	94.0697	20.0506	20	1.42297	85.9303	20.0506	11.1896	-11.1896	-0.95921	0.95921
26	19.1976	5.87286	85.0174	18.2773	0.95206	3.11215	0.30592	0.69408	354.199	84.4253	-1.7433	-16.5768	96.0034	16.6683	1.7433	16.5768	83.9966	16.6683	-20	-1.44554	94.134	20.0522	20	1.44554	85.866	20.0522	11.1896	-11.1896	-0.97442	0.97442
27	19.2	5.87358	84.9376	18.2795	0.95206	3.11216	0.30592	0.69408	354.285	84.4356	-1.77138	-16.5768	96.0994	16.6712	1.77138	16.5768	83.9006	16.6712	-20	-1.46883	94.2003	20.0539	20	1.46882	85.7997	20.0539	11.1896	-11.1896	-0.99012	0.99012
28	19.2024	5.87433	84.855	18.2818	0.95206	3.11216	0.30592	0.69408	354.377	84.4465	-1.80044	-16.5768	96.1987	16.6743	1.80044	16.5768	83.8013	16.6743	-20	-1.49292	94.269	20.0556	20	1.49292	85.731	20.0556	11.1896	-11.1896	-1.00636	1.00636
29	19.205	5.87512	84.7694	18.2843	0.95206	3.11216	0.30592	0.69408	354.472	84.4579	-1.83056	-16.5768	96.3016	16.6776	1.83056	16.5768	83.6984	16.6776	-20	-1.5179	94.3401	20.0575	20	1.5179	85.6599	20.0575	11.1896	-11.1896	-1.02314	1.02314
30	19.2078	5.87595	84.6817	18.2869	0.95206	3.11216	0.30592	0.69408	354.572	84.4698	-1.86143	-16.5768	96.407	16.681	1.86143	16.5768	83.593	16.681	-20	-1.5435	94.413	20.0595	20	1.54349	85.587	20.0595	11.1896	-11.1896	-1.04045	1.04045
31	19.2106	5.87683	84.5902	18.2897	0.95206	3.11217	0.30592	0.69408	354.678	84.4825	-1.89363	-16.5768	96.5169	16.6847	1.89363	16.5768	83.4831	16.6847	-20	-1.5702	94.4891	20.0615	20	1.5702	85.5109	20.0615	11.1896	-11.1896	-1.05845	1.05845
32	19.2137	5.87775	84.496	18.2925	0.95206	3.11217	0.30592	0.69408	354.79	84.4958	-1.92682	-16.5768	96.6301	16.6885	1.92683	16.5768	83.3699	16.6885	-20	-1.59772	94.5674	20.0637	20	1.59772	85.4326	20.0637	11.1896	-11.1896	-1.07701	1.07701
33	19.2168	5.87872	84.3985	18.2956	0.95206	3.11217	0.30591	0.69409	354.907	84.5097	-1.96118	-16.5768	96.7472	16.6925	1.96117	16.5768	83.2528	16.6925	-20	-1.62621	94.6485	20.066	20	1.6262	85.3515	20.066	11.1896	-11.1896	-1.09621	1.09621
34	19.2202	5.87974	84.2973	18.2988	0.95206	3.11217	0.30591	0.69409	355.031	84.5245	-1.99681	-16.5768	96.8686	16.6967	1.99681	16.5768	83.1314	16.6967	-20	-1.65576	94.7326	20.0684	20	1.65575	85.2674	20.0684	11.1896	-11.1896	-1.11613	1.11613
35	19.2237	5.88082	84.1923	18.3021	0.95206	3.11217	0.30591	0.69409	355.162	84.5401	-2.03385	-16.5768	96.9948	16.7011	2.03385	16.5768	83.0052	16.7011	-20	-1.68646	94.82	20.071	20	1.68646	85.18	20.071	11.1896	-11.1896	-1.13683	1.13683
36	19.2275	5.88196	84.0835	18.3057	0.95206	3.11218	0.30591	0.69409	355.3	84.5566	-2.07222	-16.5768	97.1254	16.7059	2.07223	16.5768	82.8746	16.7059	-20	-1.71829	94.9105	20.0737	20	1.71829	85.0895	20.0737	11.1896	-11.1896	-1.15828	1.15828
37	19.2315	5.88317	83.9706	18.3095	0.95206	3.11218	0.30591	0.69409	355.446	84.574	-2.11205	-16.5768	97.2609	16.7109	2.11205	16.5768	82.7391	16.7109	-20	-1.75131	95.0044	20.0765	20	1.75131	84.9956	20.0765	11.1896	-11.1896	-1.18054	1.18054
38	19.2357	5.88445	83.8533	18.3135	0.95206	3.11218	0.30591	0.69409	355.601	84.5925	-2.15346	-16.5768	97.4017	16.7161	2.15346	16.5768	82.5983	16.7161	-20	-1.78565	95.102	20.0796	20	1.78565	84.898	20.0796	11.1896	-11.1896	-1.20369	1.20369
39	19.2401	5.88581	83.7313	18.3177	0.95206	3.11219	0.30591	0.69409	355.765	84.612	-2.19652	-16.5768	97.548	16.7217	2.19652	16.5768	82.452	16.7217	-20	-1.82136	95.2034	20.0828	20	1.82135	84.7966	20.0828	11.1896	-11.1896	-1.22776	1.22776
40	19.2448	5.88724	83.6046	18.3222	0.95206	3.11219	0.30591	0.69409	355.94	84.6328	-2.2413	-16.5768	97.7001	16.7277	2.2413	16.5768	82.2999	16.7277	-20	-1.85849	95.3089	20.0862	20	1.85848	84.6911	20.0862	11.1896	-11.1896	-1.25279	1.25279
41	19.2499	5.88877	83.4726	18.327	0.95206	3.1122	0.30591	0.69409	356.125	84.6549	-2.28796	-16.5768	97.8584	16.734	2.28796	16.5768	82.1416	16.734	-20	-1.89718	95.4188	20.0898	20	1.89717	84.5812	20.0898	11.1896	-11.1896	-1.27887	1.27887
42	19.2552	5.89041	83.3345	18.3321	0.95206	3.1122	0.30591	0.69409	356.323	84.6784	-2.33681	-16.5768	98.024	16.7407	2.33681	16.5768	81.976	16.7407	-20	-1.93768	95.5338	20.0936	20	1.93768	84.4662	20.0936	11.1896	-11.1896	-1.30617	1.30617
43	19.2609	5.89214	83.1908	18.3376	0.95206	3.11221	0.30591	0.69409	356.533	84.7035	-2.38763	-16.5768	98.1962	16.7479	2.38763	16.5768	81.8038	16.7479	-20	-1.97982	95.6533	20.0978	20	1.97982	84.3466	20.0978	11.1896	-11.1896	-1.33457	1.33457
44	19.267	5.89399	83.0413	18.3433	0.95206	3.11221	0.30591	0.69409	356.758	84.7302	-2.44057	-16.5768	98.3754	16.7555	2.44057	16.5768	81.6246	16.7555	-20	-2.02372	95.7779	20.1021	20	2.02372	84.2221	20.1021	11.1896	-11.1896	-1.36417	1.36417
45	19.2735	5.89597	82.8849	18.3495	0.95206	3.11221	0.30591	0.69409	356.998	84.7587	-2.49601	-16.5768	98.5628	16.7637	2.49601	16.5768	81.4372	16.7637	-20	-2.06969	95.9082	20.1068	20	2.06969	84.0918	20.1068	11.1896	-11.1896	-1.39516	1.39516
46	19.2805	5.89809	82.7208	18.3562	0.95206	3.11222	0.30591	0.69409	357.255	84.7893	-2.55416	-16.5768	98.7593	16.7725	2.55416	16.5768	81.2407	16.7725	-20	-2.11791	96.0448	20.1118	20	2.11791	83.9552	20.1118	11.1896	-11.1896	-1.42766	1.42766
47	19.2879	5.90036	82.5495	18.3633	0.95206	3.11223	0.30591	0.69409	357.531	84.8221	-2.61497	-16.5768	98.9645	16.7818	2.61497	16.5768	81.0355	16.7818	-20	-2.16833	96.1876	20.1172	20	2.16834	83.8123	20.1172	11.1896	-11.1896	-1.46165	1.46165
48	19.2959	5.9028	82.3701	18.3709	0.95206	3.11223	0.30591	0.69409	357.827	84.8573	-2.67867	-16.5768	99.1791	16.7919	2.67867	16.5768	80.8209	16.7919	-20	-2.22115	96.3372	20.123	20	2.22115	83.6629	20.123	11.1896	-11.1896	-1.49725	1.49725
49	19.3045	5.90542	82.1814	18.3791	0.95206	3.11224	0.30591	0.69409	358.146	84.8951	-2.74574	-16.5768	99.4049	16.8027	2.74574	16.5768	80.5951	16.8027	-20	-2.27677	96.4945	20.1292	20	2.27677	83.5055	20.1292	11.1896	-11.1896	-1.53474	1.53474
50	19.3138	5.90824	81.9838	18.3879	0.95206	3.11225	0.30591	0.69409	358.489	84.9358	-2.81603	-16.5768	99.6412	16.8143	2.81603	16.5768	80.3588	16.8143	-20	-2.33505	96.6593	20.1359	20	2.33505	83.3407	20.1358	11.1896	-11.1896	-1.57403	1.57403
51	19.3238	5.91129	81.7756	18.3975	0.95206	3.11225	0.30591	0.69409	358.86	84.9799	-2.89016	-16.5768	99.8901	16.8269	2.89016	16.5768	80.1099	16.8269	-20	-2.39652	96.8329	20.1431	20	2.39652	83.1671	20.1431	11.1896	-11.1896	-1.61547	1.61547
52	19.3347	5.91459	81.5562	18.4078	0.95206	3.11226	0.30591	0.69409	359.262	85.0275	-2.96837	-16.5768	100.152	16.8405	2.96837	16.5768	79.8478	16.8405	-20	-2.46137	97.016	20.1509	20	2.46137	82.984	20.1509	11.1896	-11.1896	-1.65918	1.65918
53	19.3464	5.91817	81.3253	18.419	0.95206	3.11227	0.30591	0.69409	359.697	85.0791	-3.05081	-16.5768	100.428	16.8552	3.05081	16.5768	79.5719	16.8552	-20	-2.52973	97.2089	20.1594	20	2.52973	82.7911	20.1594	11.1896	-11.1896	-1.70526	1.70526
54	19.3591	5.92205	81.0812	18.4311	0.95206	3.11228	0.3059	0.6941	360.17	85.1351	-3.13801	-16.5768	100.719	16.8712	3.13801	16.5768	79.2807	16.8712	-20	-2.60203	97.4126	20.1686	20	2.60204	82.5873	20.1686	11.1896	-11.1896	-1.75396	1.75396
55	19.373	5.92627	80.8238	18.4443	0.95206	3.11229	0.3059	0.6941	360.685	85.196	-3.23014	-16.5768	101.026	16.8886	3.23013	16.5768	78.9736	16.8886	-20	-2.67843	97.6277	20.1786	20	2.67842	82.3723	20.1786	11.1896	-11.1896	-1.8055	1.8055
56	19.3882	5.93089	80.5503	18.4588	0.95206	3.11231	0.3059	0.6941	361.249	85.2627	-3.32815	-16.5768	101.352	16.9076	3.32815	16.5768	78.6476	16.9076	-20	-2.7597	97.8563	20.1895	20	2.7597	82.1437	20.1895	11.1896	-11.1896	-1.86028	1.86028
57	19.4047	5.93594	80.2607	18.4745	0.95206	3.11232	0.3059	0.6941	361.866	85.3356	-3.43213	-16.5768	101.697	16.9284	3.43213	16.5768	78.3026	16.9284	-20	-2.84591	98.0986	20.2015	20	2.84592	81.9014	20.2015	11.1896	-11.1896	-1.9184	1.9184
58	19.4229	5.94148	79.9529	18.4919	0.95206	3.11233	0.3059	0.6941	362.543	85.4155	-3.54281	-16.5768	102.064	16.9512	3.54281	16.5768	77.9362	16.9512	-20	-2.93769	98.3561	20.2146	20	2.93769	81.6439	20.2146	11.1896	-11.1896	-1.98027	1.98027
59	19.443	5.94758	79.6252	18.5109	0.95206	3.11235	0.3059	0.6941	363.289	85.5035	-3.6609	-16.5768	102.454	16.9763	3.6609	16.5768	77.5465	16.9763	-20	-3.03561	98.6305	20.2291	20	3.03561	81.3695	20.2291	11.1896	-11.1896	-2.04627	2.04627
60	19.4651	5.95431	79.2756	18.532	0.95206	3.11236	0.3059	0.6941	364.114	85.6006	-3.78713	-16.5768	102.869	17.0039	3.78713	16.5768	77.1311	17.0039	-20	-3.14028	98.9234	20.245	20	3.14028	81.0766	20.245	11.1896	-11.1896	-2.11683	2.11683
61	19.4896	5.96177	78.9019	18.5553	0.95207	3.11238	0.3059	0.6941	365.029	85.7083	-3.92241	-16.5768	103.312	17.0346	3.92241	16.5768	76.6875	17.0346	-20	-3.25245	99.2367	20.2627	20	3.25245	80.7633	20.2627	11.1896	-11.1896	-2.19245	2.19245
62	19.5168	5.97006	78.5013	18.5813	0.95207	3.11241	0.30589	0.69411	366.047	85.828	-4.06778	-16.5768	103.787	17.0686	4.06778	16.5768	76.2127	17.0686	-20	-3.373	99.5728	20.2824	20	3.373	80.4271	20.2824	11.1896	-11.1896	-2.27365	2.27365
63	19.5471	5.97931	78.0719	18.6102	0.95207	3.11243	0.30589	0.69411	367.185	85.9614	-4.2241	-16.5768	104.296	17.1066	4.2241	16.5768	75.7042	17.1066	-20	-3.50262	99.9335	20.3044	20	3.50262	80.0665	20.3044	11.1896	-11.1896	-2.36108	2.36108
64	19.5812	5.98968	77.6091	18.6426	0.95207	3.11245	0.30589	0.69411	368.462	86.111	-4.39309	-16.5768	104.843	17.1491	4.39309	16.5768	75.1571	17.1491	-20	-3.64274	100.323	20.329	20	3.64274	79.6775	20.329	11.1896	-11.1896	-2.45554	2.45554
65	19.6195	6.00135	77.1099	18.6791	0.95207	3.11248	0.30589	0.69411	369.902	86.2794	-4.57609	-16.5768	105.432	17.1969	4.57609	16.5768	74.5677	17.1969	-20	-3.79449	100.743	20.3568	20	3.79449	79.2573	20.3568	11.1896	-11.1896	-2.55783	2.55783
66	19.6628	6.01455	76.5691	18.7204	0.95207	3.11252	0.30588	0.69412	371.534	86.4698	-4.77513	-16.5768	106.07	17.2509	4.77513	16.5768	73.9304	17.2509	-20	-3.95953	101.198	20.3882	20	3.95953	78.8016	20.3882	11.1896	-11.1896	-2.66908	2.66908
67	19.7121	6.02955	75.9822	18.7673	0.95207	3.11255	0.30588	0.69412	373.394	86.6863	-4.99218	-16.5768	106.76	17.3122	4.99218	16.5768	73.2401	17.3122	-20	-4.13951	101.694	20.4239	20	4.13951	78.3063	20.4239	11.1896	-11.1896	-2.7904	2.7904
68	19.7684	6.0467	75.3428	18.8209	0.95207	3.1126	0.30588	0.69412	375.525	86.9338	-5.2299	-16.5768	107.51	17.3823	5.2299	16.5768	72.4898	17.3823	-20	-4.33663	102.234	20.4648	20	4.33663	77.7659	20.4648	11.1896	-11.1896	-2.92328	2.92328
69	19.8331	6.06642	74.6439	18.8826	0.95207	3.11264	0.30587	0.69413	377.984	87.2183	-5.49135	-16.5768	108.328	17.4627	5.49135	16.5768	71.6717	17.4627	-20	-4.55342	102.826	20.5118	20	4.55342	77.174	20.5118	11.1896	-11.1896	-3.06942	3.06942
70	19.9082	6.08926	73.8762	18.9541	0.95207	3.11271	0.30587	0.69413	380.843	87.5481	-5.78057	-16.5768	109.224	17.5558	5.78057	16.5768	70.7756	17.5558	-20	-4.79324	103.477	20.5664	20	4.79325	76.5225	20.5664	11.1896	-11.1896	-3.23098	3.23098
71	19.9956	6.1159	73.0309	19.0374	0.95208	3.11277	0.30586	0.69414	384.19	87.9325	-6.10162	-16.5768	110.208	17.6641	6.10163	16.5768	69.7923	17.6641	-20	-5.05946	104.196	20.63	20	5.05946	75.8035	20.63	11.1896	-11.1896	-3.41048	3.41048
72	20.0985	6.14724	72.095	19.1353	0.95208	3.11283	0.30586	0.69414	388.144	88.3845	-6.46048	-16.5768	111.292	17.7913	6.46048	16.5768	68.7077	17.7913	-20	-5.35702	104.995	20.705	20	5.35702	75.0052	20.705	11.1896	-11.1896	-3.61111	3.61111
73	20.2206	6.18442	71.0535	19.2516	0.95208	3.11292	0.30585	0.69415	392.864	88.9211	-6.86431	-16.5768	112.494	17.9419	6.86432	16.5768	67.506	17.9419	-20	-5.69188	105.886	20.7942	20	5.69188	74.114	20.7942	11.1896	-11.1896	-3.83679	3.83679
74	20.3668	6.22898	69.8894	19.3909	0.95208	3.11302	0.30584	0.69416	398.556	89.5639	-7.32173	-16.5768	113.83	18.1218	7.32172	16.5768	66.1697	18.1218	-20	-6.07117	106.886	20.9012	20	6.07116	73.1139	20.9012	11.1896	-11.1896	-4.09251	4.09251
75	20.5442	6.28301	68.5792	19.5598	0.95209	3.11314	0.30583	0.69417	405.514	90.3434	-7.84474	-16.5768	115.325	18.3394	7.84474	16.5768	64.6749	18.3394	-20	-6.50485	108.017	21.0312	20	6.50485	71.9833	21.0312	11.1896	-11.1896	-4.38485	4.38485
76	20.7619	6.34933	67.0964	19.7672	0.95209	3.11328	0.30582	0.69418	414.138	91.3003	-8.44815	-16.5768	117.005	18.6055	8.44815	16.5768	62.995	18.6055	-20	-7.00519	109.303	21.1913	20	7.00519	70.6967	21.1913	11.1896	-11.1896	-4.72213	4.72213
77	21.0332	6.43197	65.4061	20.0256	0.9521	3.11345	0.3058	0.6942	425.01	92.4926	-9.15234	-16.5768	118.904	18.9356	9.15234	16.5768	61.0962	18.9356	-20	-7.58911	110.78	21.3915	20	7.58911	69.2205	21.3915	11.1896	-11.1896	-5.11574	5.11574
78	21.3766	6.5366	63.4664	20.3527	0.9521	3.11365	0.30578	0.69422	438.976	94.002	-9.98433	-16.5768	121.061	19.3514	9.98433	16.5768	58.9392	19.3514	-20	-8.27899	112.487	21.6458	20	8.27899	67.5129	21.6458	11.1896	-11.1896	-5.58078	5.58078
79	21.82	6.67167	61.2223	20.775	0.95211	3.11391	0.30576	0.69424	457.339	95.9506	-10.9828	-16.5768	123.526	19.885	10.9828	16.5768	56.474	19.885	-20	-9.10693	114.482	21.9758	20	9.10693	65.518	21.9758	11.1896	-11.1896	-6.13889	6.13889
80	22.4053	6.84999	58.605	21.3325	0.95212	3.11424	0.30573	0.69427	482.159	98.5232	-12.2033	-16.5768	126.359	20.5843	12.2033	16.5768	53.6408	20.5843	-20	-10.119	116.837	22.4141	20	10.119	63.163	22.4141	11.1896	-11.1896	-6.82096	6.82096
81	23.1987	7.09179	55.5278	22.0882	0.95213	3.11462	0.3057	0.6943	516.856	102.011	-13.7286	-16.5768	129.631	21.5236	13.7286	16.5768	50.3691	21.5236	-20	-11.3838	119.648	23.0128	20	11.3838	60.352	23.0128	11.1896	-11.1896	-7.67358	7.67358
82	24.3096	7.43037	51.881	23.1461	0.95214	3.11507	0.30566	0.69434	567.463	106.893	-15.6896	-16.5768	133.425	22.8245	15.6896	16.5768	46.575	22.8245	-20	-13.0098	123.044	23.8591	20	13.0098	56.9563	23.8591	11.1896	-11.1896	-8.7698	8.7698
83	25.9281	7.92373	47.5291	24.6876	0.95216	3.11566	0.3056	0.6944	645.43	114.007	-18.3044	-16.5768	137.835	24.695	18.3043	16.5768	42.1647	24.695	-20	-15.1779	127.195	25.1072	20	15.1779	52.8053	25.1072	11.1896	-11.1896	-10.2313	10.2313
84	28.4101	8.68044	42.3129	27.0515	0.95218	3.11637	0.30554	0.69446	774.754	124.917	-21.9655	-16.5768	142.959	27.5186	21.9655	16.5768	37.041	27.5186	-20	-18.2137	132.324	27.0507	20	18.2137	47.6763	27.0507	11.1896	-11.1896	-12.2777	12.2777
85	32.4869	9.92368	36.0649	30.9341	0.9522	3.1172	0.30547	0.69453	1012.82	142.838	-27.4569	-16.5768	148.879	32.0729	27.4569	16.5768	31.1211	32.0729	-20	-22.7672	138.702	30.3042	20	22.7672	41.2979	30.3042	11.1896	-11.1896	-15.3471	15.3471
86	39.8971	12.184	28.6436	37.9912	0.95223	3.11812	0.30539	0.69461	1527.15	175.412	-36.6091	-16.5768	155.639	40.1873	36.6091	16.5768	24.3613	40.1873	-20	-30.3562	146.621	36.3524	20	30.3562	33.3786	36.3524	11.1896	-11.1896	-20.4628	20.4628
87	55.8955	17.0653	20.0084	53.2267	0.95225	3.11901	0.30531	0.69469	2996.68	245.742	-54.9139	-16.5768	163.203	57.3614	54.9139	16.5768	16.7975	57.3614	-20	-45.5345	156.288	49.7332	20	45.5345	23.7124	49.7332	11.1896	-11.1896	-30.6943	30.6943
88	106.77	32.5914	10.3187	101.674	0.95227	3.11967	0.30525	0.69475	10932.1	469.398	-109.828	-16.5768	171.417	111.072	109.828	16.5768	8.58316	111.072	-20	-91.0689	167.614	93.2391	20	91.0689	12.3863	93.2391	11.1896	-11.1896	-61.3886	61.3886

Axial Displacement	1. General ellipse parameter features										2. features extracted from locus																			
faulty disk	A`	B`	θ	f	e	e'	$\frac{e}{e'}$	g	$A_{ellipse}$	$c_{ellipse}$	I_{min}	ΔV_{Imin}	θ_{Imin}	Abs_{Imin}	I_{max}	ΔV_{Imax}	θ_{Imax}	Abs_{Imax}	ΔV_{min}	$I_{\Delta Vmin}$	$\theta_{\Delta Vmin}$	$Abs_{\Delta Vmi}$	Δv_{max}	$I_{\Delta Vmax}$	$\theta_{\Delta Vmax}$	$Abs_{\Delta Vma}$	$\Delta V_{H(0)}$	$\Delta V_{L(0)}$	$I_{H(0)}$	$I_{L(0)}$
1	11.8026	3.59616	86.4258	11.2414	0.95245	3.12595	0.30469	0.69531	133.342	51.8748	-0.7692	-10.2252	94.302	10.254	0.7692	10.2252	85.698	10.254	-12.3143	-0.63869	92.969	12.3309	12.3143	0.63801	87.0341	12.3308	6.86211	-6.86211	-0.42867	0.42867
2	11.7998	3.58724	86.4212	11.2413	0.95267	3.1337	0.30401	0.69599	132.98	51.8461	-0.76977	-10.225	94.3053	10.254	0.76977	10.225	85.6947	10.254	-12.309	-0.63958	92.9744	12.3256	12.309	0.63958	87.0256	12.3256	6.85275	-6.85275	-0.42836	0.42836
3	11.7977	3.57853	86.4171	11.2418	0.95289	3.14147	0.30333	0.69667	132.633	51.8202	-0.77028	-10.2362	94.3034	10.2651	0.77028	10.2362	85.6966	10.2651	-12.3044	-0.64109	92.9826	12.321	12.3044	0.64109	87.0174	12.321	6.82771	-6.82771	-0.42701	0.42701
4	11.7955	3.56979	86.4131	11.2424	0.95311	3.14931	0.30264	0.69736	132.285	51.7944	-0.77078	-10.2473	94.3016	10.2763	0.77078	10.2473	85.6984	10.2763	-12.2997	-0.64191	92.9875	12.3165	12.2997	0.64191	87.0125	12.3165	6.80266	-6.80266	-0.42668	0.42668
5	11.7985	3.56089	86.4121	11.2483	0.95337	3.15885	0.30181	0.69819	131.989	51.7877	-0.77133	-10.2474	94.3046	10.2764	0.77133	10.2474	85.6954	10.2764	-12.2947	-0.64345	92.9959	12.3115	12.2947	0.64278	87.0072	12.3115	6.79341	-6.79341	-0.42635	0.42635
6	11.7909	3.55215	86.4047	11.2432	0.95354	3.16517	0.30126	0.69874	131.58	51.7413	-0.77183	-10.2585	94.3027	10.2875	0.77183	10.2585	85.6973	10.2875	-12.2902	-0.64428	93.0008	12.307	12.2902	0.64428	86.9992	12.307	6.76835	-6.76835	-0.425	0.425
7	11.7944	3.52501	86.4041	11.2553	0.95429	3.19299	0.29887	0.70113	130.613	51.6995	-0.77233	-10.2591	94.3053	10.2881	0.77233	10.2591	85.6948	10.2881	-12.2857	-0.64511	93.0058	12.3026	12.2857	0.64511	86.9942	12.3026	6.75937	-6.75937	-0.42465	0.42465
8	11.7867	3.51618	86.3966	11.25	0.95447	3.1995	0.29832	0.70168	130.201	51.6526	-0.77284	-10.27	94.3035	10.2991	0.77284	10.27	85.6965	10.2991	-12.281	-0.64595	93.0108	12.2979	12.281	0.64594	86.9892	12.2979	6.71801	-6.71801	-0.4233	0.4233
9	11.7899	3.50728	86.3957	11.2561	0.95473	3.20936	0.29748	0.70252	129.906	51.6466	-0.77338	-10.2808	94.302	10.3099	0.77338	10.2808	85.698	10.3099	-12.2762	-0.64747	93.0191	12.2932	12.2762	0.64747	86.9809	12.2932	6.70883	-6.70883	-0.42296	0.42296
10	11.7877	3.49842	86.3917	11.2566	0.95494	3.21762	0.29679	0.70321	129.554	51.6206	-0.77388	-10.2812	94.3046	10.3103	0.77388	10.2812	85.6954	10.3103	-12.2715	-0.64831	93.0241	12.2886	12.2715	0.64897	86.9728	12.2887	6.69973	-6.69973	-0.4226	0.4226
11	11.7855	3.48954	86.3875	11.2571	0.95516	3.22596	0.29609	0.70391	129.201	51.5945	-0.77441	-10.2921	94.303	10.3212	0.77441	10.2921	85.697	10.3212	-12.2669	-0.64982	93.0323	12.2841	12.2669	0.64982	86.9677	12.2841	6.67443	-6.67443	-0.42124	0.42124
12	11.7832	3.48056	86.3832	11.2574	0.95538	3.23436	0.29538	0.70462	128.843	51.5676	-0.77494	-10.2924	94.3059	10.3215	0.77494	10.2924	85.6941	10.3215	-12.262	-0.65068	93.0376	12.2793	12.262	0.65068	86.9624	12.2793	6.66515	-6.66515	-0.42089	0.42089
13	11.7811	3.47168	86.3792	11.258	0.9556	3.24281	0.29468	0.70532	128.492	51.5419	-0.77545	-10.3033	94.3041	10.3324	0.77545	10.3033	85.6959	10.3324	-12.2575	-0.65152	93.0426	12.2748	12.2575	0.65152	86.9574	12.2748	6.63988	-6.63988	-0.41951	0.41951
14	11.7847	3.46286	86.3786	11.2645	0.95585	3.25294	0.29384	0.70616	128.205	51.5379	-0.77594	-10.3144	94.3022	10.3435	0.77594	10.3144	85.6978	10.3435	-12.2532	-0.653	93.0505	12.2706	12.2532	0.653	86.9495	12.2706	6.61473	-6.61473	-0.41915	0.41915
15	11.782	3.43535	86.3741	11.2701	0.95655	3.28062	0.29158	0.70842	127.157	51.4727	-0.77651	-10.3144	94.3053	10.3435	0.77651	10.3143	85.6947	10.3435	-12.248	-0.65455	93.059	12.2655	12.248	0.65389	86.944	12.2655	6.60519	-6.60519	-0.4188	0.4188
16	11.7801	3.42645	86.3701	11.2707	0.95676	3.28934	0.29087	0.70913	126.807	51.4476	-0.77701	-10.3253	94.3036	10.3545	0.77701	10.3253	85.6964	10.3545	-12.2436	-0.65538	93.064	12.2612	12.2436	0.65538	86.936	12.2612	6.5799	-6.5799	-0.4174	0.4174
17	11.7778	3.4174	86.3659	11.2711	0.95698	3.29815	0.29016	0.70984	126.447	51.4209	-0.77754	-10.3359	94.3021	10.3651	0.77754	10.3359	85.6979	10.3651	-12.2389	-0.65624	93.0692	12.2565	12.2389	0.65624	86.9308	12.2565	6.55435	-6.55435	-0.41704	0.41704
18	11.7756	3.40837	86.3617	11.2716	0.9572	3.30703	0.28944	0.71056	126.09	51.3947	-0.77807	-10.3363	94.3049	10.3655	0.77807	10.3363	85.6951	10.3655	-12.2342	-0.65775	93.0774	12.2519	12.2342	0.65775	86.9226	12.2519	6.54507	-6.54507	-0.41563	0.41563
19	11.7739	3.39947	86.3579	11.2724	0.95741	3.31593	0.28873	0.71127	125.742	51.3704	-0.77855	-10.3473	94.3029	10.3765	0.77855	10.3473	85.6971	10.3765	-12.2301	-0.65856	93.0823	12.2478	12.2301	0.65856	86.9177	12.2478	6.51981	-6.51981	-0.41525	0.41525
20	11.7718	3.39043	86.3537	11.2729	0.95763	3.32493	0.28801	0.71199	125.385	51.3444	-0.77907	-10.3477	94.3056	10.377	0.77907	10.3477	85.6944	10.377	-12.2255	-0.65942	93.0874	12.2432	12.2255	0.65941	86.9126	12.2432	6.51052	-6.51052	-0.41488	0.41488
21	11.7694	3.38127	86.3494	11.2732	0.95784	3.33403	0.28729	0.71271	125.021	51.3174	-0.77962	-10.3581	94.3043	10.3874	0.77962	10.3581	85.6957	10.3874	-12.2206	-0.66094	93.0958	12.2385	12.2206	0.66029	86.9073	12.2385	6.48483	-6.48483	-0.41347	0.41347
22	11.7677	3.37232	86.3456	11.2741	0.95806	3.34314	0.28657	0.71343	124.672	51.2932	-0.78011	-10.3691	94.3025	10.3984	0.78011	10.3691	85.6975	10.3984	-12.2165	-0.66176	93.1006	12.2344	12.2165	0.66241	86.8963	12.2344	6.45948	-6.45948	-0.41309	0.41309
23	11.7655	3.36316	86.3413	11.2746	0.95827	3.35237	0.28585	0.71415	124.31	51.2667	-0.78064	-10.3694	94.3053	10.3987	0.78064	10.3694	85.6947	10.3987	-12.2118	-0.66327	93.1089	12.2298	12.2118	0.66327	86.8911	12.2298	6.45007	-6.45007	-0.41167	0.41167
24	11.7635	3.35407	86.3372	11.2752	0.95849	3.36164	0.28513	0.71487	123.953	51.2411	-0.78116	-10.38	94.3037	10.4094	0.78116	10.38	85.6963	10.4094	-12.2073	-0.66411	93.114	12.2254	12.2073	0.66411	86.886	12.2254	6.42448	-6.42448	-0.41129	0.41129
25	11.7615	3.32664	86.3332	11.2813	0.95917	3.39119	0.28284	0.71716	122.919	51.1797	-0.78167	-10.3907	94.3022	10.42	0.78167	10.3907	85.6978	10.42	-12.2029	-0.6656	93.1221	12.2211	12.2029	0.6656	86.8779	12.2211	6.3989	-6.3989	-0.40986	0.40986
26	11.7648	3.31754	86.3324	11.2874	0.95942	3.40233	0.28199	0.71801	122.617	51.1743	-0.78218	-10.3912	94.3047	10.4206	0.78218	10.3912	85.6953	10.4206	-12.1986	-0.66644	93.1271	12.2168	12.1986	0.66644	86.8729	12.2168	6.38962	-6.38962	-0.40946	0.40946
27	11.7629	3.30841	86.3284	11.288	0.95963	3.41192	0.28126	0.71874	122.259	51.149	-0.7827	-10.4018	94.3032	10.4312	0.7827	10.4018	85.6968	10.4312	-12.1942	-0.66793	93.1352	12.2124	12.1942	0.66793	86.8648	12.2125	6.36398	-6.36398	-0.40907	0.40907
28	11.7609	3.29926	86.3243	11.2887	0.95985	3.42158	0.28053	0.71947	121.901	51.1237	-0.78321	-10.4124	94.3016	10.4418	0.78321	10.4124	85.6984	10.4418	-12.1898	-0.66878	93.1403	12.2081	12.1898	0.66878	86.8597	12.2081	6.33831	-6.33831	-0.40763	0.40763
29	11.759	3.29009	86.3203	11.2894	0.96006	3.43132	0.27979	0.72021	121.543	51.0984	-0.78372	-10.4129	94.3042	10.4424	0.78372	10.4129	85.6958	10.4424	-12.1854	-0.66962	93.1454	12.2038	12.1854	0.66962	86.8546	12.2038	6.32896	-6.32896	-0.40722	0.40722
30	11.7569	3.28083	86.316	11.2899	0.96027	3.44116	0.27906	0.72094	121.179	51.0723	-0.78426	-10.4232	94.3029	10.4527	0.78426	10.4233	85.6971	10.4527	-12.1809	-0.67113	93.1536	12.1993	12.1809	0.67113	86.8464	12.1993	6.30312	-6.30312	-0.40577	0.40577
31	11.7549	3.27158	86.3119	11.2905	0.96049	3.45108	0.27832	0.72168	120.817	51.0468	-0.78479	-10.4238	94.3056	10.4533	0.78479	10.4238	85.6944	10.4533	-12.1764	-0.67198	93.1588	12.195	12.1764	0.67198	86.8412	12.195	6.29371	-6.29371	-0.40538	0.40538
32	11.7531	3.26241	86.308	11.2913	0.9607	3.46102	0.27758	0.72242	120.46	51.0221	-0.78529	-10.4343	94.304	10.4638	0.78529	10.4343	85.696	10.4638	-12.1722	-0.67345	93.1668	12.1908	12.1722	0.67282	86.8362	12.1908	6.268	-6.268	-0.4039	0.4039
33	11.7513	3.25319	86.304	11.292	0.96092	3.47107	0.27684	0.72316	120.101	50.9973	-0.7858	-10.4448	94.3025	10.4743	0.7858	10.4448	85.6975	10.4743	-12.168	-0.6743	93.1718	12.1866	12.168	0.6743	86.8282	12.1866	6.24224	-6.24224	-0.40349	0.40349
34	11.7545	3.24394	86.3031	11.298	0.96117	3.48281	0.27597	0.72403	119.791	50.9913	-0.78631	-10.4454	94.305	10.4749	0.78631	10.4454	85.695	10.4749	-12.1636	-0.67514	93.1769	12.1824	12.1636	0.67514	86.8231	12.1824	6.23282	-6.23282	-0.40202	0.40202
35	11.7475	3.21629	86.2957	11.2986	0.96179	3.51292	0.27379	0.72621	118.7	50.911	-0.78685	-10.4557	94.3037	10.4852	0.78685	10.4557	85.6963	10.4852	-12.1592	-0.67664	93.1851	12.178	12.1592	0.67664	86.8149	12.178	6.20688	-6.20688	-0.4016	0.4016
36	11.7507	3.20705	86.295	11.3046	0.96204	3.52493	0.27292	0.72708	118.392	50.9056	-0.78735	-10.4661	94.3022	10.4957	0.78735	10.4661	85.6978	10.4957	-12.155	-0.67748	93.1901	12.1739	12.155	0.67747	86.8099	12.1739	6.18109	-6.18109	-0.40118	0.40118
37	11.749	3.19778	86.291	11.3054	0.96225	3.53539	0.27218	0.72782	118.032	50.8809	-0.78786	-10.4668	94.3047	10.4964	0.78786	10.4668	85.6953	10.4964	-12.1509	-0.67895	93.1982	12.1698	12.1509	0.67895	86.8018	12.1698	6.17166	-6.17166	-0.3997	0.3997
38	11.747	3.18838	86.2868	11.306	0.96246	3.54599	0.27142	0.72858	117.665	50.8552	-0.7884	-10.4769	94.3034	10.5066	0.7884	10.4769	85.6966	10.5066	-12.1464	-0.67981	93.2034	12.1654	12.1464	0.67981	86.7966	12.1654	6.14564	-6.14564	-0.39928	0.39928
39	11.7451	3.17901	86.2827	11.3067	0.96267	3.55666	0.27067	0.72933	117.3	50.8299	-0.78892	-10.4871	94.3021	10.5168	0.78892	10.4871	85.6979	10.5168	-12.1421	-0.6813	93.2115	12.1612	12.1421	0.6813	86.7885	12.1612	6.11965	-6.11965	-0.39778	0.39778
40	11.7434	3.16972	86.2788	11.3076	0.96288	3.56737	0.26991	0.73009	116.941	50.8058	-0.78943	-10.4879	94.3046	10.5176	0.78943	10.4879	85.6954	10.5176	-12.138	-0.68276	93.2195	12.1572	12.138	0.68214	86.7835	12.1572	6.11023	-6.11023	-0.39735	0.39735
41	11.7416	3.16032	86.2747	11.3083	0.9631	3.57821	0.26916	0.73084	116.575	50.7807	-0.78995	-10.4981	94.3032	10.5278	0.78995	10.4981	85.6968	10.5278	-12.1338	-0.68361	93.2246	12.153	12.1338	0.68299	86.7783	12.153	6.08422	-6.08422	-0.39584	0.39584
42	11.74	3.15101	86.2709	11.3092	0.96331	3.58908	0.2684	0.7316	116.217	50.7569	-0.79044	-10.499	94.3055	10.5382	0.79044	10.5085	85.6984	10.5382	-12.1298	-0.68443	93.2295	12.1491	12.1298	0.68443	86.7705	12.1491	6.07483	-6.07483	-0.3954	0.3954
43	11.738	3.14148	86.2666	11.3098	0.96352	3.60013	0.26763	0.73237	115.845	50.7308	-0.79099	-10.5089	94.3045	10.5386	0.79099	10.5089	85.6955	10.5386	-12.1253	-0.68594	93.2378	12.1447	12.1253	0.68532	86.7651	12.1446	6.04861	-6.04861	-0.39389	0.39389
44	11.7364	3.13213	86.2627	11.3107	0.96373	3.6112	0.26687	0.73313	115.484	50.7069	-0.79149	-10.5192	94.303	10.549	0.79149	10.5192	85.697	10.549	-12.1213	-0.68677	93.2428	12.1408	12.1213	0.68677	86.7572	12.1408	6.02264	-6.02264	-0.39345	0.39345
45	11.7347	3.10441	86.2588	11.3166	0.96437	3.64533	0.26455	0.73545	114.446	50.6476	-0.792	-10.5199	94.3054	10.5497	0.792	10.5199	85.6946	10.5497	-12.1172	-0.68761	93.2479	12.1367	12.1172	0.68761	86.7521	12.1367	6.01311	-6.01311	-0.39301	0.39301
46	11.7377	3.09491	86.2577	11.3223	0.96461	3.65836	0.26367	0.73633	114.125	50.641	-0.79254	-10.53	94.3043	10.5597	0.79254	10.53	85.6957	10.5597	-12.1129	-0.6891	93.256	12.1325	12.1129	0.6891	86.744	12.1325	5.98693	-5.98693	-0.39148	0.39148
47	11.7363	3.08555	86.254	11.3234	0.96482	3.66982	0.26291	0.73709	113.766	50.6178	-0.79302	-10.5403	94.3027	10.5701	0.79302	10.5403	85.6973	10.5701	-12.1091	-0.69053	93.2638	12.1288	12.1092	0.68991	86.7391	12.1288	5.96097	-5.96097	-0.39102	0.39102
48	11.7343	3.07597	86.2498	11.324	0.96503	3.68144	0.26213	0.73787	113.394	50.5923	-0.79357	-10.5409	94.3054	10.5707	0.79357	10.5408	85.6946	10.5707	-12.1048	-0.6914	93.2691	12.1245	12.1048	0.69141	86.7309	12.1245	5.95123	-5.95123	-0.38949	0.38949
49	11.7329	3.06655	86.2459	11.325	0.96524	3.69309	0.26136	0.73864	113.033	50.5688	-0.79407	-10.5511	94.3039	10.5809	0.79407	10.5511	85.6961	10.5809	-12.101	-0.69224	93.2741	12.1208	12.101	0.69224	86.7259	12.1208	5.92519	-5.92519	-0.38904	0.38904
50	11.7314	3.05711	86.2422	11.3261	0.96545	3.70483	0.26059	0.73941	112.671	50.5455	-0.79456	-10.5613	94.3024	10.5912	0.79456	10.5613	85.6976	10.5912	-12.0971	-0.69367	93.2819	12.117	12.0972	0.69367	86.7182	12.117	5.89912	-5.89912	-0.38749	0.38749
51	11.7297	3.04755	86.2381	11.3269	0.96566	3.71673	0.25981	0.74019	112.302	50.5209	-0.79509	-10.562	94.305	10.5919	0.79508	10.562	85.695	10.5919	-12.093	-0.69453	93.287	12.113	12.093	0.69453	86.713	12.113	5.88944	-5.88944	-0.38702	0.38702
52	11.728	3.03794	86.234	11.3277	0.96587	3.72873	0.25903	0.74097	111.931	50.4961	-0.79562	-10.5719	94.3039	10.6018	0.79562	10.5719	85.6961	10.6018	-12.0889	-0.69601	93.2951	12.1089	12.0889	0.69601	86.7049	12.1089	5.86311	-5.86311	-0.38547	0.38547
53	11.7263	3.02836	86.23	11.3285	0.96608	3.7408	0.25825	0.74175	111.563	50.4717	-0.79614	-10.5818	94.3026	10.6117	0.79614	10.5818	85.6974	10.6117	-12.0848	-0.69685	93.3002	12.1049	12.0848	0.69685	86.6998	12.1049	5.83681	-5.83681	-0.38501	0.38501
54	11.725	3.01888	86.2263	11.3297	0.96629	3.75294	0.25747	0.74253	111.201	50.4489	-0.79663	-10.5828	94.3048	10.6128	0.79663	10.5828	85.6952	10.6128	-12.0811	-0.69828	93.308	12.1013	12.0811	0.69828	86.692	12.1013	5.82731	-5.82731	-0.38343	0.38343
55	11.7235	3.00931	86.2224	11.3307	0.96649	3.7652	0.25669	0.74331	110.834	50.4251	-0.79713	-10.5928	94.3035	10.6228	0.79713	10.5928	85.6965	10.6228	-12.0773	-0.69912	93.313	12.0975	12.0773	0.69912	86.687	12.0975	5.80105	-5.80105	-0.38295	0.38295
56	11.7219	2.98142	86.2184	11.3364	0.96711	3.80235	0.25435	0.74565	109.792	50.367	-0.79766	-10.6027	94.3023	10.6327	0.79766	10.6027	85.6977	10.6327	-12.0733	-0.70057	93.321	12.0936	12.0733	0.70057	86.679	12.0936	5.77469	-5.77469	-0.38138	0.38138
57	11.7251	2.97183	86.2175	11.3422	0.96735	3.81657	0.25346	0.74654	109.469	50.3613	-0.79816	-10.6036	94.3047	10.6336	0.79816	10.6036	85.6953	10.6336	-12.0695	-0.70141	93.326	12.0898	12.0695	0.70141	86.674	12.0898	5.76504	-5.76504	-0.3809	0.3809
58	11.7235	2.96217	86.2136	11.3431	0.96755	3.82932	0.25267	0.74733	109.098	50.3373	-0.79868	-10.6135	94.3035	10.6435	0.79868	10.6135	85.6965	10.6435	-12.0655	-0.70227	93.3311	12.086	12.0655	0.70227	86.6689	12.086	5.73865	-5.73865	-0.38042	0.38042
59	11.722	2.95252	86.2096	11.3441	0.96776	3.84218	0.25188	0.74812	108.729	50.3136	-0.7992	-10.6233	94.3023	10.6533	0.7992	10.6233	85.6977	10.6533	-12.0617	-0.70371	93.339	12.0822	12.0617	0.70371	86.661	12.0822	5.71226	-5.71226	-0.37883	0.37883
60	11.7205	2.94282	86.2057	11.345	0.96797	3.85515	0.25108	0.74892	108.357	50.2897	-0.79972	-10.6241	94.3048	10.6542	0.79972	10.6241	85.6953	10.6542	-12.0578	-0.70456	93.3441	12.0783	12.0578	0.70456	86.6559	12.0783	5.70248	-5.70248	-0.37834	0.37834
61	11.7189	2.93312	86.2017	11.3459	0.96817	3.86821	0.25029	0.74971	107.986	50.2659	-0.80024	-10.6339	94.3036	10.664	0.80024	10.6339	85.6964	10.664	-12.0539	-0.706	93.352	12.0746	12.0539	0.706	86.648	12.0746	5.67601	-5.67601	-0.37674	0.37674
62	11.7178	2.92352	86.198	11.3472	0.96838	3.88135	0.24949	0.75051	107.622	50.2437	-0.80073	-10.6439	94.3022	10.674	0.80073	10.6439	85.6978	10.674	-12.0504	-0.70683	93.3569	12.0711	12.0504	0.70742	86.6403	12.0711	5.64972	-5.64972	-0.37624	0.37624
63	11.7163	2.91379	86.1941	11.3482	0.96858	3.89464	0.2487	0.7513	107.25	50.2199	-0.80124	-10.6447	94.3046	10.6748	0.80124	10.6447	85.6954	10.6748	-12.0465	-0.70826	93.3648	12.0673	12.0465	0.70826	86.6352	12.0673	5.63991	-5.63991	-0.37464	0.37464
64	11.7151	2.90414	86.1904	11.3494	0.96879	3.90801	0.2479	0.7521	106.884	50.1975	-0.80174	-10.6547	94.3032	10.6848	0.80174	10.6547	85.6968	10.6848	-12.043	-0.70909	93.3697	12.0639	12.043	0.70909	86.6303	12.0639	5.61355	-5.61355	-0.37413	0.37413
65	11.7135	2.89432	86.1863	11.3503	0.96899	3.92157	0.24709	0.75291	106.508	50.1734	-0.80227	-10.6642	94.3022	10.6944	0.80227	10.6642	85.6978	10.6944	-12.0391	-0.71054	93.3776	12.06	12.0391	0.71054	86.6224	12.06	5.5869	-5.5869	-0.37252	0.37252
66	11.7123	2.86639	86.1826	11.3561	0.96959	3.96183	0.24473	0.75527	105.47	50.1176	-0.80276	-10.6653	94.3044	10.6955	0.80276	10.6653	85.6956	10.6955	-12.0356	-0.71136	93.3825	12.0566	12.0356	0.71136	86.6175	12.0566	5.57721	-5.57721	-0.372	0.372
67	11.7152	2.85654	86.1815	11.3616	0.96982	3.97742	0.24383	0.75617	105.133	50.1108	-0.8033	-10.6749	94.3035	10.705	0.8033	10.6749	85.6965	10.705	-12.0317	-0.71281	93.3905	12.0528	12.0317	0.71281	86.6095	12.0528	5.55053	-5.55053	-0.37038	0.37038
68	11.714	2.84681	86.1778	11.3628	0.97002	3.99143	0.24303	0.75697	104.765	50.0885	-0.80379	-10.6847	94.3022	10.7148	0.80379	10.6846	85.6978	10.7148	-12.0282	-0.71364	93.3954	12.0493	12.0282	0.71364	86.6046	12.0493	5.52404	-5.52404	-0.36986	0.36986
69	11.7126	2.83696	86.1739	11.3638	0.97022	4.00563	0.24221	0.75779	104.389	50.065	-0.80431	-10.6855	94.3046	10.7158	0.80431	10.6855	85.6954	10.7158	-12.0244	-0.71507	93.4033	12.0457	12.0244	0.71507	86.5967	12.0456	5.51414	-5.51414	-0.36823	0.36823
70	11.7112	2.82711	86.1699	11.3649	0.97043	4.01996	0.2414	0.7586	104.014	50.0418	-0.80483	-10.6951	94.3035	10.7253	0.80483	10.6951	85.6965	10.7253	-12.0207	-0.71592	93.4084	12.042	12.0207	0.71592	86.5916	12.042	5.48745	-5.48745	-0.36772	0.36772
71	11.7099	2.81726	86.1661	11.3659	0.97063	4.03439	0.24059	0.75941	103.641	50.0188	-0.80534	-10.7047	94.3024	10.7349	0.80534	10.7047	85.6976	10.7349	-12.0171	-0.71734	93.4161	12.0384	12.0171	0.71734	86.5839	12.0385	5.46078	-5.46078	-0.36606	0.36606
72	11.7088	2.8075	86.1624	11.3672	0.97083	4.04889	0.23978	0.76022	103.272	49.997	-0.80583	-10.7058	94.3046	10.7361	0.80583	10.7058	85.6954	10.7361	-12.0137	-0.71816	93.421	12.0351	12.0137	0.71816	86.579	12.0351	5.45105	-5.45105	-0.36553	0.36553
73	11.7074	2.79756	86.1585	11.3682	0.97103	4.06362	0.23896	0.76104	102.894	49.9736	-0.80636	-10.7153	94.3036	10.7456	0.80636	10.7153	85.6964	10.7456	-12.01	-0.71959	93.4288	12.0315	12.01	0.71959	86.5712	12.0315	5.42424	-5.42424	-0.36388	0.36388
74	11.7064	2.78776	86.1548	11.3696	0.97123	4.0784	0.23814	0.76186	102.524	49.9519	-0.80685	-10.725	94.3023	10.7553	0.80685	10.725	85.6977	10.7553	-12.0066	-0.72041	93.4337	12.0282	12.0066	0.72041	86.5663	12.0282	5.39764	-5.39764	-0.36335	0.36335
75	11.7048	2.77774	86.1507	11.3704	0.97143	4.09342	0.23732	0.76268	102.142	49.9279	-0.8074	-10.7257	94.3049	10.7561	0.8074	10.7257	85.6951	10.7561	-12.0028	-0.72128	93.4389	12.0244	12.0028	0.72185	86.5583	12.0245	5.38755	-5.38755	-0.36168	0.36168
76	11.7039	2.76792	86.1472	11.3719	0.97163	4.10845	0.2365	0.7635	101.773	49.9066	-0.80788	-10.7354	94.3036	10.7658	0.80788	10.7354	85.6964	10.7658	-11.9996	-0.72266	93.4464	12.0213	11.9996	0.72266	86.5536	12.0213	5.36094	-5.36094	-0.36114	0.36114
77	11.7068	2.75793	86.1461	11.3773	0.97185	4.12531	0.23558	0.76442	101.431	49.9	-0.8084	-10.7448	94.3026	10.7752	0.8084	10.7448	85.6974	10.7752	-11.9959	-0.72351	93.4515	12.0177	11.9959	0.72408	86.5458	12.0178	5.33405	-5.33405	-0.35947	0.35947
78	11.706	2.7299	86.1426	11.3832	0.97243	4.16982	0.23321	0.76679	100.393	49.8465	-0.80888	-10.7461	94.3046	10.7765	0.80888	10.7461	85.6954	10.7765	-11.9928	-0.72489	93.459	12.0147	11.9928	0.72489	86.541	12.0147	5.32432	-5.32432	-0.35891	0.35891
79	11.7049	2.71999	86.1389	11.3845	0.97263	4.18549	0.23238	0.76762	100.02	49.8247	-0.80938	-10.7557	94.3035	10.7861	0.80937	10.7557	85.6965	10.7861	-11.9895	-0.72628	93.4665	12.0115	11.9895	0.72628	86.5335	12.0115	5.29756	-5.29756	-0.35723	0.35723
80	11.7037	2.70998	86.1351	11.3856	0.97282	4.20137	0.23155	0.76845	99.6413	49.8023	-0.8099	-10.765	94.3025	10.7954	0.8099	10.765	85.6975	10.7954	-11.986	-0.72713	93.4716	12.0081	11.986	0.72713	86.5284	12.0081	5.27064	-5.27064	-0.35668	0.35668
81	11.7025	2.69994	86.1312	11.3867	0.97302	4.2174	0.23072	0.76928	99.2617	49.7796	-0.81042	-10.766	94.3049	10.7965	0.81042	10.766	85.6951	10.7965	-11.9825	-0.72853	93.4793	12.0046	11.9825	0.72853	86.5207	12.0046	5.2606	-5.2606	-0.35499	0.35499
82	11.7013	2.68993	86.1274	11.3879	0.97322	4.23355	0.22988	0.77012	98.8839	49.7575	-0.81094	-10.7753	94.3039	10.8058	0.81094	10.7753	85.6961	10.8058	-11.9791	-0.72938	93.4843	12.0013	11.9791	0.72938	86.5157	12.0013	5.23367	-5.23367	-0.35443	0.35443
83	11.7001	2.67984	86.1235	11.3891	0.97342	4.2499	0.22904	0.77096	98.5028	49.7349	-0.81146	-10.7845	94.303	10.815	0.81146	10.7845	85.697	10.815	-11.9756	-0.73078	93.492	11.9979	11.9756	0.73078	86.508	11.9979	5.20665	-5.20665	-0.35272	0.35272
84	11.6994	2.66996	86.1201	11.3906	0.97361	4.26623	0.22821	0.77179	98.1334	49.7146	-0.81193	-10.786	94.3049	10.8165	0.81192	10.786	85.6951	10.8165	-11.9727	-0.73158	93.4967	11.995	11.9727	0.73158	86.5033	11.995	5.19687	-5.19687	-0.35215	0.35215
85	11.6982	2.65984	86.1162	11.3918	0.97381	4.28288	0.22737	0.77263	97.7517	49.6922	-0.81246	-10.7951	94.304	10.8257	0.81246	10.7952	85.696	10.8257	-11.9692	-0.73244	93.5018	11.9916	11.9692	0.73299	86.4956	11.9916	5.16981	-5.16981	-0.35044	0.35044
86	11.6973	2.64981	86.1126	11.3932	0.974	4.29962	0.22653	0.77347	97.3751	49.6709	-0.81295	-10.8045	94.3029	10.835	0.81295	10.8045	85.6971	10.835	-11.966	-0.73381	93.5092	11.9885	11.966	0.73381	86.4908	11.9885	5.14287	-5.14287	-0.34987	0.34987
87	11.6962	2.63969	86.1088	11.3944	0.9742	4.31657	0.22569	0.77431	96.9942	49.649	-0.81347	-10.8056	94.3053	10.8362	0.81347	10.8056	85.6947	10.8362	-11.9627	-0.73521	93.5169	11.9853	11.9627	0.73521	86.4831	11.9853	5.13278	-5.13278	-0.34815	0.34815
88	11.6994	2.62963	86.1079	11.4	0.97441	4.33521	0.22477	0.77523	96.651	49.6436	-0.81397	-10.8149	94.3042	10.8455	0.81397	10.8149	85.6958	10.8455	-11.9596	-0.73603	93.5217	11.9822	11.9596	0.73603	86.4783	11.9822	5.10581	-5.10581	-0.34758	0.34758
